# Comparative effects of 18 antipsychotics on metabolic function in patients with schizophrenia, predictors of metabolic dysregulation, and association with psychopathology: a systematic review and network meta-analysis

**DOI:** 10.1016/S2215-0366(19)30416-X

**Published:** 2020-01

**Authors:** Toby Pillinger, Robert A McCutcheon, Luke Vano, Yuya Mizuno, Atheeshaan Arumuham, Guy Hindley, Katherine Beck, Sridhar Natesan, Orestis Efthimiou, Andrea Cipriani, Oliver D Howes

**Affiliations:** a Institute of Psychiatry, Psychology and Neuroscience, King's College London, London, UK; b MRC London Institute of Medical Sciences, Faculty of Medicine, Imperial College London, Hammersmith Hospital Campus, London, UK; c Department of Neuropsychiatry, Keio University School of Medicine, Tokyo, Japan; d Institute of Social and Preventive Medicine, University of Bern, Bern, Switzerland; e Department of Psychiatry, University of Oxford, Oxford, UK; f Oxford health NHS Foundation Trust, Warneford Hospital, Oxford, UK

## Abstract

**Background:**

Antipsychotic treatment is associated with metabolic disturbance. However, the degree to which metabolic alterations occur in treatment with different antipsychotics is unclear. Predictors of metabolic dysregulation are poorly understood and the association between metabolic change and change in psychopathology is uncertain. We aimed to compare and rank antipsychotics on the basis of their metabolic side-effects, identify physiological and demographic predictors of antipsychotic-induced metabolic dysregulation, and investigate the relationship between change in psychotic symptoms and change in metabolic parameters with antipsychotic treatment.

**Methods:**

We searched MEDLINE, EMBASE, and PsycINFO from inception until June 30, 2019. We included blinded, randomised controlled trials comparing 18 antipsychotics and placebo in acute treatment of schizophrenia. We did frequentist random-effects network meta-analyses to investigate treatment-induced changes in body weight, BMI, total cholesterol, LDL cholesterol, HDL cholesterol, triglyceride, and glucose concentrations. We did meta-regressions to examine relationships between metabolic change and age, sex, ethnicity, baseline weight, and baseline metabolic parameter level. We examined the association between metabolic change and psychopathology change by estimating the correlation between symptom severity change and metabolic parameter change.

**Findings:**

Of 6532 citations, we included 100 randomised controlled trials, including 25 952 patients. Median treatment duration was 6 weeks (IQR 6–8). Mean differences for weight gain compared with placebo ranged from −0·23 kg (95% CI −0·83 to 0·36) for haloperidol to 3·01 kg (1·78 to 4·24) for clozapine; for BMI from −0·25 kg/m^2^ (−0·68 to 0·17) for haloperidol to 1·07 kg/m^2^ (0·90 to 1·25) for olanzapine; for total-cholesterol from −0·09 mmol/L (−0·24 to 0·07) for cariprazine to 0·56 mmol/L (0·26–0·86) for clozapine; for LDL cholesterol from −0·13 mmol/L (−0.21 to −0·05) for cariprazine to 0·20 mmol/L (0·14 to 0·26) for olanzapine; for HDL cholesterol from 0·05 mmol/L (0·00 to 0·10) for brexpiprazole to −0·10 mmol/L (−0·33 to 0·14) for amisulpride; for triglycerides from −0·01 mmol/L (−0·10 to 0·08) for brexpiprazole to 0·98 mmol/L (0·48 to 1·49) for clozapine; for glucose from −0·29 mmol/L (−0·55 to −0·03) for lurasidone to 1·05 mmol/L (0·41 to 1·70) for clozapine. Greater increases in glucose were predicted by higher baseline weight (p=0·0015) and male sex (p=0·0082). Non-white ethnicity was associated with greater increases in total cholesterol (p=0·040) compared with white ethnicity. Improvements in symptom severity were associated with increases in weight (*r*=0·36, p=0·0021), BMI (*r*=0·84, p<0·0001), total-cholesterol (*r*=0·31, p=0·047), and LDL cholesterol (*r*=0·42, p=0·013), and decreases in HDL cholesterol (*r*=–0·35, p=0·035).

**Interpretation:**

Marked differences exist between antipsychotics in terms of metabolic side-effects, with olanzapine and clozapine exhibiting the worst profiles and aripiprazole, brexpiprazole, cariprazine, lurasidone, and ziprasidone the most benign profiles. Increased baseline weight, male sex, and non-white ethnicity are predictors of susceptibility to antipsychotic-induced metabolic change, and improvements in psychopathology are associated with metabolic disturbance. Treatment guidelines should be updated to reflect our findings. However, the choice of antipsychotic should be made on an individual basis, considering the clinical circumstances and preferences of patients, carers, and clinicians.

**Funding:**

UK Medical Research Council, Wellcome Trust, National Institute for Health Research Oxford Health Biomedical Research Centre.

**Research in context**

**Evidence before this study**

Antipsychotic drug treatment possibly causes glucose dysregulation and lipid disturbance, thereby contributing to the development of the metabolic syndrome in patients with schizophrenia. However, the degree to which metabolic alterations occur in patients treated with various antipsychotics remains unclear. Furthermore, whether baseline patient characteristics can predict metabolic dysregulation is unknown, and the association between metabolic change and change in psychopathology is uncertain. To address these issues, we searched PubMed using the keywords “schizophrenia AND antipsychotic AND (glucose OR cholesterol OR triglycerides OR metabolic)”, from inception until July 19, 2019, and without language restriction. Selection criteria were network meta-analyses of randomised blinded trials examining antipsychotic treatment of patients with schizophrenia, in which outcomes were change in glucose, cholesterol, or triglyceride concentrations. Of the 664 studies retrieved, 1 network meta-analysis was identified, which examined only a single parameter (glucose concentrations). No studies examined baseline predictors of metabolic change, or the relationship between metabolic change and change in psychopathology.

**Added value of this study**

Our findings show variations in antipsychotics in terms of their metabolic side-effects, and identify increasing age, male sex, and non-white ethnicity as possible risk factors for antipsychotic-induced metabolic dysregulation. Furthermore, we identified strong evidence that antipsychotic-associated improvements in psychopathology are associated with metabolic disturbance.

**Implications of all the available evidence**

Considering the increased prevalence of metabolic syndrome, cardiovascular disease, and cardiovascular mortality in patients with schizophrenia, data from this study might be used to inform antipsychotic prescribing, especially in the at-risk groups we have identified. However, clinical decisions to use preferentially an antipsychotic with fewer metabolic side-effects should consider that clinical improvement appears to be associated with development of these side-effects.

## Introduction

Antipsychotics form the mainstay of treatment for patients with schizophrenia, but many, especially the second-generation antipsychotics, are associated with weight gain, lipid disturbance, and glucose dysregulation, thereby contributing to the development of metabolic syndrome.^1^ Approximately a third of people with schizophrenia have metabolic syndrome, with prevalence as high as 69% in those with chronic illness.^2^ The prevalence of obesity, type 2 diabetes, and hypercholesterolaemia in people with schizophrenia is estimated to be 3–5 times higher than in the general population.^3^ Compared with the general population, people with schizophrenia are twice as likely to have a diagnosis and die as a consequence of cardiovascular disease.^4^ The mortality gap between people with schizophrenia and the general population is growing,^5^ suggesting a need for improved understanding of the factors underlying cardiovascular disease in this group. Although studies have previously examined changes in weight with different antipsychotics,^6^ no study has comprehensively examined antipsychotic-induced metabolic change (ie, glucose, cholesterol, and triglyceride alterations) using network meta-analysis. Thus, the relative degree to which metabolic alterations occur in acute treatment with various antipsychotics is unclear. Furthermore, which physiological or demographic factors predict metabolic dysregulation associated with antipsychotics is unknown. Previous studies assessing comparative efficacies of various antipsychotics have used separate network meta-analyses to examine symptom change and change in weight.^6^ However, to date, no meta-analysis that has synthesised metabolic and symptom-change data has been done. Whether an association exists between antipsychotic-induced metabolic dysregulation and symptom change in patients, as suggested by some, but not all previous longitudinal studies,^7^, ^8^, ^9^ is unclear. We did a network meta-analysis of trials comparing antipsychotics in the treatment of schizophrenia, aiming to investigate the relative effects of various drugs on body weight, body-mass index (BMI), and metabolic measures (fasting glucose, total cholesterol, LDL cholesterol, HDL cholesterol, and triglycerides). We also did bivariate meta-analyses and meta-regression analyses of placebo-controlled data to investigate whether baseline demographic and physiological factors predict the magnitude of antipsychotic-induced metabolic change, and whether a relationship exists between metabolic change and change in severity of psychotic symptoms during antipsychotic treatment.

## Methods

### Search strategy and selection criteria

We followed the PRISMA^10^ extension statement for network meta-analysis (appendix p 2). We searched MEDLINE, EMBASE, and PsycINFO from inception until June 30, 2019. Our search strategy is described fully in the appendix (p 5), but in brief we used the following search terms: (antipsychotic OR [generic/branded antipsychotic names]) AND (schizo* OR psychos*) AND (random* or ‘double blind’). We included randomised double-blind trials comparing antipsychotics licensed for the treatment of schizophrenia in adults with acute exacerbation of schizophrenia or a related disorder (schizoaffective, schizophreniform, and delusional disorders). We defined acute treatment as 6-weeks' duration.^6^ If 6-week data were not available, data closest to 6 weeks were selected. Clinical trials registry data relating to papers identified in the literature review were included.

### Data extraction and processing

Pairs of independent investigators (LV, KB, AA, GH, YM, and TP) screened references and extracted study-level data, with discrepancies adjudicated by TP. We extracted outcome data (expressed as mean and SD, standard error, or CIs) for change in body weight (kg), BMI (kg/m^2^), fasting glucose, total cholesterol, LDL cholesterol, HDL cholesterol, and triglycerides (all mmol/L) from initiation to end of treatment for groups who received drugs and placebo separately. Only continuous data were collected, not binary outcomes. We did not use dose limits because of the scarce evidence that dose influences metabolic dysregulation.^11^ For multi-group studies reporting several doses of an antipsychotic, a summary value for a given metabolic parameter for all doses was calculated using formulae from the Cochrane Handbook (appendix p 5).^12^ Because paliperidone is the active metabolite of risperidone,^13^ data for these drugs were combined as previously described.^14^ We also extracted publication year; total symptom change (mean variance, measured using the Positive and Negative Syndrome Scale [PANSS] or Brief Psychiatric Rating Scale [BPRS]); baseline weight and baseline metabolic parameter level; study duration; antipsychotic drug; study population (first-episode psychosis, multi-episode schizophrenia, treatment-resistant schizophrenia, or older adults); age; gender; and ethnicity. Authors were contacted to request unreported data.

### Data analysis

#### Pairwise meta-analysis

All analyses were done in R (version 3.5.1). For pairwise comparisons informed by ten or more studies, we synthesised data in a meta-analysis using a random-effects model in the metafor package (version 2.1–0). We investigated the heterogeneity of treatment effects visually by inspecting forest plots, alongside monitoring of τ (SD of random effects) and the *I*^2^ statistic. To visualise heterogeneity, prediction intervals were included in forest plots. Small study effects and publication bias were assessed by visual inspection of contour-enhanced funnel plots and using Egger's test.

#### Assessment of the transitivity assumption

Transitivity is the key underlying assumption of network meta-analysis.^16^ To assess this assumption, we examined the distribution of possible effect modifiers across treatment comparisons. Potential effect modifiers included age, sex, ethnicity, and body weight.^17^, ^18^, ^19^

#### Network meta-analysis

We fitted random-effects frequentist network meta-analyses, in which we assumed a common random-effects SD (τ) for all comparisons in the network. We fitted our models in R using netmeta (version 1.0–1).^20^, ^21^ Metabolic change for each parameter and each treatment comparison was estimated as mean difference with 95% CIs. We avoided dichotomising results as statistically significant or not, and instead presented results with CIs to allow clinicians to gauge the range of likely effects.^22^, ^23^ Placebo was used as the reference treatment in all forest plots. We created league tables to display the relative degree of metabolic disturbance for all comparisons among antipsychotics. We used P-scores to rank antipsychotics on the basis of the degree of metabolic dysregulation.^24^ P-scores ranged from 0 to 1, a higher P-score indicating a greater degree of metabolic disturbance. To provide an overview of results, we generated a heatmap summarising ranking of disturbance across all metabolic parameters for all antipsychotics. For most parameters, a higher P-score indicates a greater increase in that parameter; however, because increased HDL cholesterol reduces cardiovascular disease risk,^25^ a higher P-score for this parameter indicates a smaller increase.

#### Assessment of heterogeneity and inconsistency in the network

We assessed network heterogeneity using τ and *I*^2^ statistic. To visualise heterogeneity, we used prediction intervals in all forest plots. We assessed the presence of network consistency using a global (design-by-treatment inconsistency model) and a local method (back calculation).^26^, ^27^

#### Risk of bias in network analysis

We assessed risk of bias of individual studies using the Cochrane Collaboration's Tool for Assessing Risk of Bias,^28^ classifying risk of bias as high, moderate, or low (appendix p 5). We incorporated results into the Confidence in Network Meta-Analysis (CINeMA)^29^, ^30^ application to assess the credibility of findings from each network meta-analysis. CINeMA grades confidence in results of each treatment comparison as high, moderate, low, or very low (appendix p 19).

#### Sensitivity analysis

We hypothesised that the inclusion of various study populations might contribute to heterogeneity and inconsistency. Thus, we assessed the sensitivity of our findings by repeating each network meta-analysis after excluding studies examining first-episode psychosis, treatment-resistant schizophrenia, and older adults (aged 65 years and older).

#### Meta-regression: baseline predictors of antipsychotic-associated metabolic alterations

In the general population, body weight, age, sex, and ethnicity influence metabolic function.^17^, ^18^, ^19^ Therefore, we investigated whether these covariates, as well as treatment factors, were related to change in metabolic parameters. Using the metafor package (version 2.0.0),^31^ we did meta-regressions using placebo-controlled data aiming to examine the relationship between antipsychotic-associated metabolic change and baseline body weight, baseline level of a given parameter (eg, baseline-glucose if examining glucose change), age, sex, and ethnicity. In these meta-regressions, if a study had multiple active groups, estimates for each group were merged.^12^

#### Assessing the relationship between alterations in metabolic parameters and psychopathology

The relationship between metabolic change and psychopathology change is uncertain. To examine whether these two outcomes are associated, we did additional bivariate meta-analyses using placebo-controlled data. We meta-analysed the mean difference for change in weight, BMI, metabolic parameter, and standardised mean difference for change in total symptoms (assessed using PANSS or BPRS). Given that within-study correlations between the outcomes were not reported, we used a model proposed by Riley and colleagues,^32^ which overcomes this problem, using the package metamisc (version 0.2.0).

This study was registered with PROSPERO (CRD42019125322, appendix p 5).

### Role of the funding source

The funders had no role in study design, data collection, data analysis, data interpretation, or writing of the report. The corresponding author had full access to all the data in the study and had final responsibility for the decision to submit for publication.

## Results

Of 6512 citations retrieved, 100 (1·5%) studies met the inclusion criteria, examining the following: amisulpride, aripiprazole, asenapine, brexpiprazole, cariprazine, clozapine, flupenthixol, fluphenazine, haloperidol, iloperidone, lurasidone, olanzapine, quetiapine, risperidone, paliperidone, sertindole, ziprasidone, and zotepine (appendix pp 20–21). The overall sample included 25 952 participants (21 124 who were antipsychotic treated, 4828 who were placebo treated). The mean age was 35·03 years (SD 6·05), 14 922 (57·50%) of 25 952 participants were men, 11 030 (42·5%) were women, 11 537 (63·56%) of 18 151 with reported ethnicity were white, and 6614 (36·44%) were non-white. Treatment duration was 2–13 weeks (median 6 weeks [IQR 6–8]). Risk of bias was high for 16% of datasets (appendix p 34).

**Figure 1:**
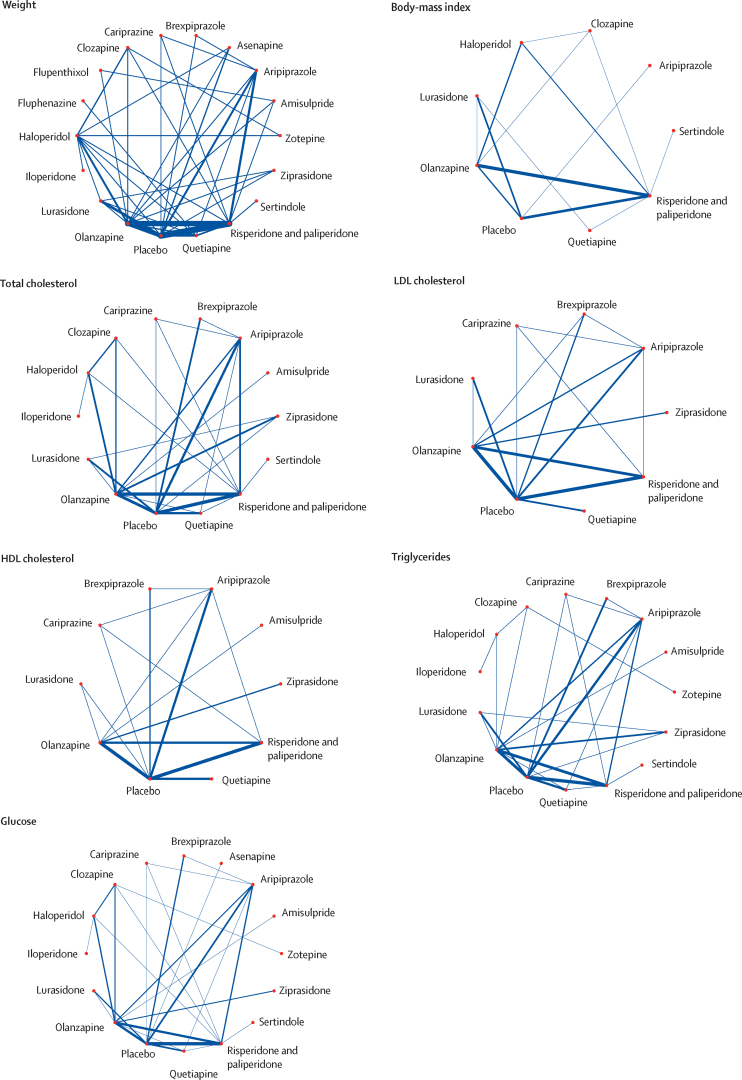
Network graphs for weight, body-mass index, total cholesterol, LDL cholesterol, HDL cholesterol, triglycerides, and glucose Treatments with direct comparisons are linked with a line; the thickness of connecting lines corresponds to the number of trials evaluating the comparison.

**Figure 2:**
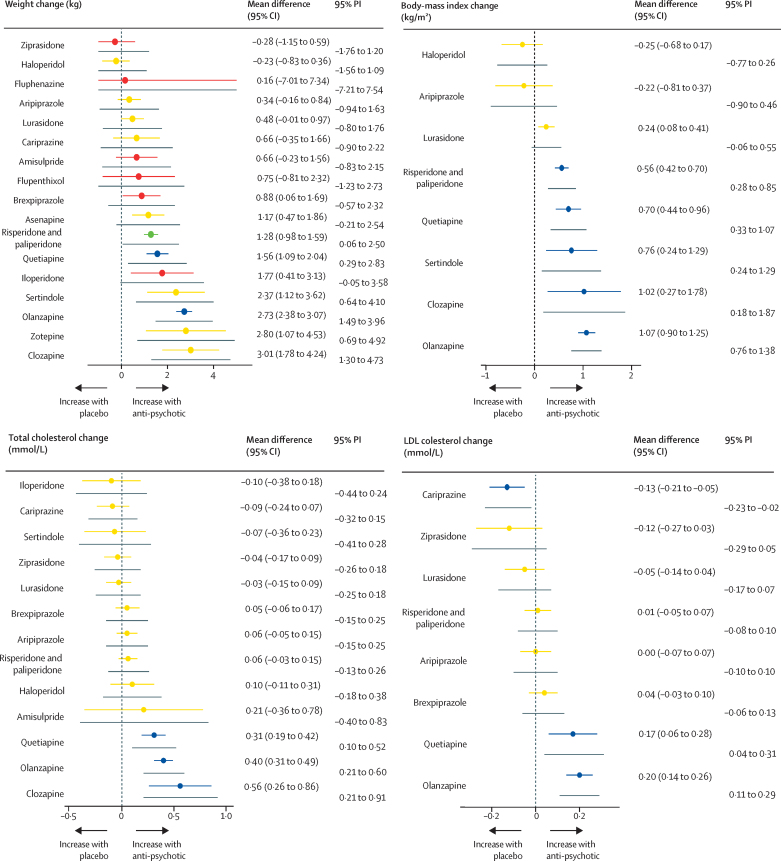

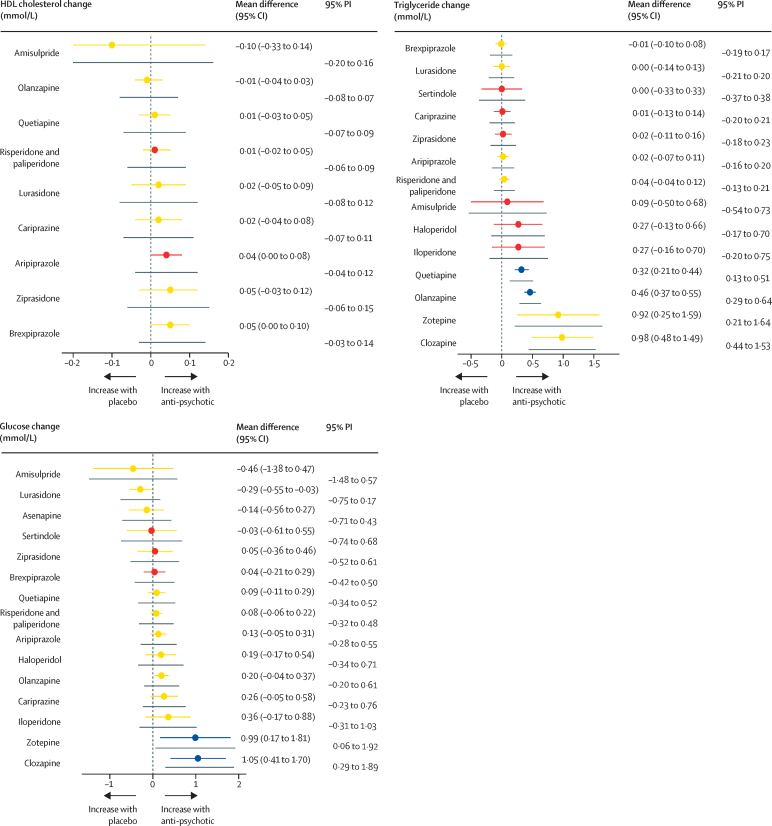
Forest plots for mean differences of antipsychotic drugs compared with placebo Colours indicate the confidence in the evidence for a given comparison: green is high, blue is moderate, yellow is low, and red is very low. Confidence of outcomes was graded using the Confidence in Network Meta-Analysis application. Grey lines immediately below each coloured line indicate the PI corresponding to that antipsychotic–placebo comparison. Full results for all treatment comparisons are shown in the appendix (pp 41-46). PI=prediction interval.

**Figure 3:**
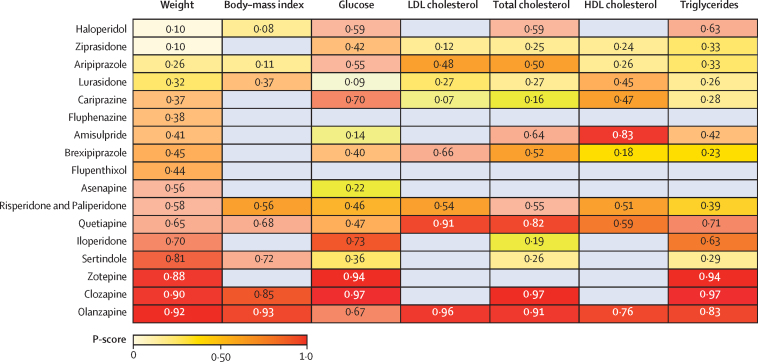
Heat map of antipsychotic drugs ranked according to associated degree of alteration in bodyweight, body-mass index, and metabolic parameters Numbers reflect P-score, which rank antipsychotics on a continuous scale from 0 to 1. A higher P-score indicates a greater increase in the metabolic parameter, with the exception of HDL cholesterol, for which a higher P-score indicates a smaller increase. Grey squares indicate that data were not available.

The age and sex of participants were similarly distributed across treatment comparisons (appendix p 36). Ethnicity and baseline weight differed across treatment comparisons, but overall, we deemed the sample similar enough to synthesise jointly. We did three pairwise comparisons with ten studies or more, all for weight change. The results of the meta-analyses and assessment of between-study heterogeneity and small study effects and publication bias are described in the appendix (pp 37–40). We found evidence of small study effects and publication bias for the comparison of change in body weight with placebo and olanzapine. The corresponding contour-enhanced funnel plot showed an absence of studies published with statistically insignificant (p>0·10) outcomes, and Egger's regression test suggested funnel plot asymmetry (z=2·50, p=0·01). Network graphs are shown in figure 1. Forest plots for the mean change in metabolic parameter for antipsychotics with placebo as the reference treatment are shown in figure 2, and league tables comparing antipsychotics for each parameter in the appendix (pp 41–46). P-value rankings of antipsychotics for all metabolic parameters are shown collectively in a heatmap in figure 3 and individually in the appendix (pp 47–49). Local assessments of inconsistency are shown in the appendix (pp 50–61). CINeMA confidence ratings are shown in the appendix (pp 62–87) and were used to colour code forest plots in figure 2.

For change in weight, 83 studies compared 18 different antipsychotics (18 750 patients) with placebo (4210 patients). We did not find evidence of weight gain with ziprasidone, haloperidol, fluphenazine, aripiprazole, lurasidone, cariprazine, amisulpride, or flupenthixol when compared with placebo. We found evidence of weight gain with brexpiprazole, risperidone and paliperidone, quetiapine, iloperidone, sertindole, olanzapine, zotepine, and clozapine (figure 2; appendix p 41). Ranking on the basis of degree of weight gain identified haloperidol as the best and clozapine the worst (figure 3; appendix p 47). τ was 0·59 kg, considered small in the context of the observed antipsychotic-associated changes, and I^2^ was 71·3% (moderate to substantial). The global Q score for inconsistency was 97·41 (p<0·0001), and significant hotspots of inconsistency were identified in five (3·2%) of 154 treatment comparisons, including some disagreements between direct and indirect evidence (appendix p 50). Certainty of evidence was low or very low in 140 (91%) of 154 comparisons (appendix p 62). A post-hoc analysis excluding studies at high risk of bias showed that estimated treatment effects were broadly similar, and heterogeneity and inconsistency assessments did not materially change (appendix pp 88–92).

For change in BMI, 22 studies compared nine different antipsychotics (4196 patients) with placebo (900 patients). Compared with placebo, no change in BMI was observed with haloperidol or aripiprazole. BMI increased with lurasidone, risperidone and paliperidone, quetiapine, sertindole, clozapine, and olanzapine (figure 2; appendix p 42). Ranking on the basis of degree of associated BMI alteration identified haloperidol as the best and olanzapine the worst (figure 3; appendix p 47). τ was 0·32kg/m^2^, considered moderate in the context of the observed antipsychotic-associated changes, and I^2^ 31·4% (low). Inspection of prediction intervals confirmed that heterogeneity was low, because for most treatment comparisons prediction intervals and CIs led to similar conclusions. The global Q score for inconsistency was 8·93 (p=0·54), and the back-calculation method did not provide evidence of network inconsistency (appendix p 53). Thus, we deemed that no evidence existed for important heterogeneity or inconsistency in this network meta-analysis. Certainty of evidence was low or very low in 18 (50%) of 36 comparisons (appendix p 69).

For change in total cholesterol, 36 studies compared 14 different antipsychotics (11 762 patients) with placebo (2998 patients). Compared with placebo, we found no evidence of change in total cholesterol observed with iloperidone, cariprazine, sertindole, ziprasidone, lurasidone, brexpiprazole, aripiprazole, risperidone and paliperidone, haloperidol, and amisulpride. Total cholesterol increased with quetiapine, olanzapine, and clozapine (figure 2; appendix p 43). Ranking on the basis of degree of associated total cholesterol alteration identified cariprazine as the best and clozapine the worst (figure 3; appendix p 47). τ was 0·08 mmol/L, considered small in the context of the observed antipsychotic-associated changes, and I^2^ was 45·1% (moderate). Conclusions drawn from prediction intervals and CIs agreed. The global Q score for inconsistency was 35·55 (p=0·017). However, out of 91 treatment comparisons, we identified only a single hotspot of inconsistency showing disagreement between indirect and direct evidence (appendix p 54). Thus, overall, we concluded that heterogeneity and inconsistency were not a source of concern in this network meta-analysis. Certainty of evidence was low in 65 (71%) of 91 comparisons (appendix p 71).

For change in LDL cholesterol, 24 studies compared nine different antipsychotics (7439 patients) with placebo (2419 patients). Compared with placebo, we found no strong evidence of change in LDL cholesterol with ziprasidone, lurasidone, risperidone and paliperidone, aripiprazole, and brexpiprazole (figure 2). We did observe a decrease in LDL cholesterol with cariprazine (figure 2). We observed increases in LDL cholesterol with quetiapine and olanzapine (figure 2; appendix p 44). Ranking on the basis of degree of associated LDL cholesterol alteration defined cariprazine as the best and olanzapine the worst (figure 3; appendix p 48). τ was 0·03 mmol/L, considered small in the context of observed antipsychotic-associated changes. The prediction intervals did not change our conclusions when compared with CIs, and I^2^ was 16·2% (low). The global Q score for inconsistency was 4·46 (p=0·92), and although we found some disagreements between direct and indirect evidence (appendix p 56), overall, we found no evidence of important heterogeneity or inconsistency in the network. Certainty of evidence was low in 19 (53%) of 36 comparisons (appendix p 73).

For change in HDL cholesterol, 22 studies compared ten different antipsychotics (7073 patients) with placebo (2189 patients). Compared with placebo, we found no strong evidence of change in HDL cholesterol observed with amisulpride, olanzapine, quetiapine, risperidone and paliperidone, lurasidone, cariprazine, or ziprasidone (figure 2). HDL cholesterol increased with aripiprazole and brexpiprazole (figure 2; appendix p 44). Ranking on the basis of degree of associated HDL cholesterol alteration defined brexpiprazole as the best and amisulpride the worst (figure 3; appendix p 48). τ was 0·03 mmol/L, considered medium to large in the context of the observed antipsychotic-associated changes, and I^2^ was 52·3% (moderate). The global Q score for inconsistency was 18·96 (p=0·025), and out of 45 treatment comparisons, we identified two hotspots of inconsistency, although in both cases, direct and indirect evidence pointed in the same direction (appendix p 57). Certainty of evidence was low or very low in 100% of comparisons (appendix p 75).

For change in triglycerides, 34 studies compared 15 different antipsychotics (10 965 patients) with placebo (3021 patients). Compared with placebo, we found no strong evidence of change in triglyceride concentrations with brexpiprazole, lurasidone, sertindole, cariprazine, ziprasidone, aripiprazole, risperidone and paliperidone, amisulpride, haloperidol, and iloperidone (figure 2). Triglyceride concentrations increased with quetiapine, olanzapine, zotepine, and clozapine (figure 2; appendix p 45). Ranking on the basis of degree of associated triglyceride alteration identified brexpiprazole as the best and clozapine the worst (figure 3; appendix p 48). τ was 0·07 mmol/L, considered small in the context of the observed antipsychotic-associated changes, and I^2^ was 42·6% (moderate). The global Q score for inconsistency was 45·07 (p<0·0001), but out of 105 treatment comparisons, we only identified four hotspots of inconsistency showing disagreement between indirect and direct evidence (appendix p 58). Certainty of evidence was low or very low in 97 (92%) of 105 comparisons (appendix p 77).

For change in fasting-glucose, 37 studies compared 16 different antipsychotics (10 681 patients) with placebo (3032 patients). Compared with placebo, we found no strong evidence change in glucose concentrations with amisulpride, asenapine, sertindole, ziprasidone, brexpiprazole, quetiapine, risperidone and paliperidone, aripiprazole, haloperidol, cariprazine, and iloperidone (figure 2). Glucose concentrations reduced with lurasidone, and increased with olanzapine, zotepine, and clozapine (figure 2; appendix p 46). Ranking on the basis of degree of associated glucose alteration defined lurasidone as the best and clozapine the worst (figure 3; appendix p 48). τ was 0·18 mmol/L, considered moderate in the context of the observed antipsychotic-associated changes, and *I*^2^ was 62·7% (moderate). The global Q score for inconsistency was 55·58 (p<0·0001), but out of 120 treatment comparisons, only one significant hotspot of inconsistency was identified with disagreement between indirect and direct evidence (appendix p 60). Certainty of evidence was low or very low in 103 (86%) of 120 comparisons (appendix p 82).

The sensitivity of our findings for all seven network meta-analysis outcomes was assessed by repeating analyses after the exclusion of studies examining patients with first-episode psychosis (four studies), treatment-resistant schizophrenia (five studies), and older adults (two studies). The findings essentially remained the same in all sensitivity analyses (appendix pp 88–97), indicating that the inclusion of these studies did not have a major influence on results. Assessments of heterogeneity and inconsistency were also broadly similar, except for LDL cholesterol, for which the global test of inconsistency worsened (Q 22·67, p=0·030) and triglycerides, for which the global test of inconsistency improved (Q 4·53, p=0·98), although local tests of inconsistency were materially unchanged (appendix p 93).

**Figure 4:**
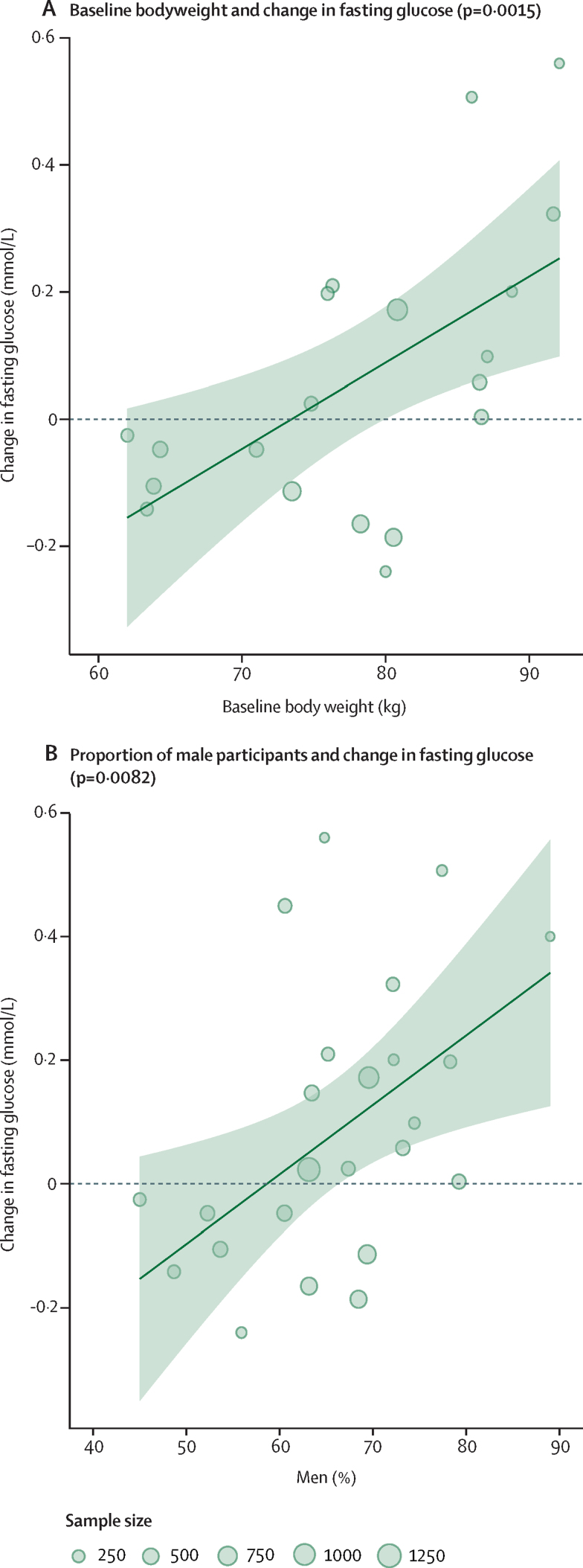
Bubble plots for meta-regressions on the effect of baseline predictors on antipsychotic-induced changes in fasting-glucose Each bubble corresponds to a study. The size of the bubble is proportional to the sample size. The solid line corresponds to the meta-regression estimate, and corresponding 95% CI, indicated by light-green shading.

Greater antipsychotic-induced increases in fasting-glucose levels were associated with higher baseline body-weight (study number [k]=20, z=3·18, estimate=0·01 kg^−1^ [95% CI 0·00–0·02], p=0·0015; figure 4A) and larger proportion of male participants (k=25, z=2·64, estimate=0·01 [0·00–0·02], p=0.0082; figure 4B). Greater antipsychotic-induced increases in total cholesterol were associated with a larger proportion of non-white participants (k=22, z=2·05, estimate=0·003 [mmol/L]^−1^ [0·00–0·01], p=0·040). We did not find strong evidence of an association between change in weight, BMI, LDL cholesterol, HDL cholesterol, and triglycerides with any baseline variables.

**Figure 5:**
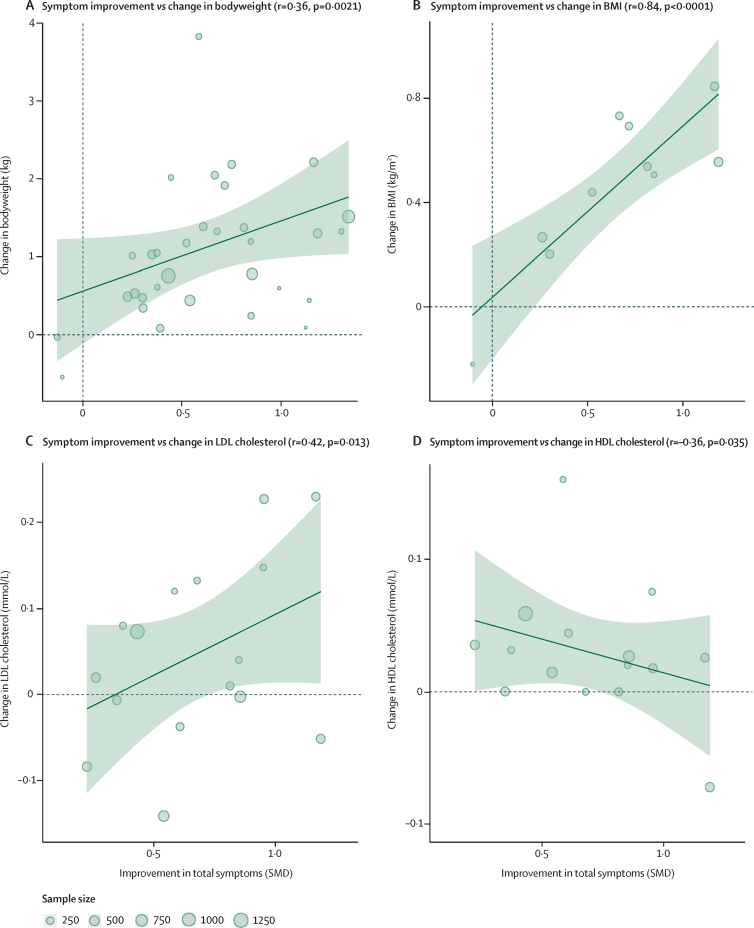
Bubble plots for the associations between change in symptom severity and change in metabolic parameters Each bubble corresponds to a study. The size of the bubble is proportional to the sample size. The solid line corresponds to the meta-regression estimate, and corresponding 95% CI, indicated by light-green shading. SMD=standard mean difference.

Greater improvement in total symptom severity was strongly associated with greater increases in body weight (figure 5A), BMI (figure 5B), total cholesterol (*r*=0·31 [df 29], p=0·047), and LDL cholesterol (figure 5C), and with greater reductions in HDL cholesterol (figure 5D). We did not find evidence of an association between symptom change and changes in triglyceride or glucose concentrations.

## Discussion

We found that antipsychotics vary markedly in their effects on body weight, BMI, total cholesterol, LDL cholesterol, HDL cholesterol, triglycerides, and glucose concentrations. As expected,^6^ clozapine and olanzapine are, across virtually all parameters, associated with the largest degree of metabolic dysregulation. However, for several antipsychotics we did not find evidence of an effect versus placebo in terms of lipid or glucose measures. Interestingly, some of the drugs were shown to perform better than placebo on some metabolic measures: for instance, when compared with placebo, lurasidone led to reductions in glucose, cariprazine to reductions in LDL cholesterol, and aripiprazole and brexpiprazole to increases in HDL cholesterol. Our meta-analysis is the first to examine predictors of antipsychotic-induced metabolic change. We found that increased baseline bodyweight, male sex, and non-white ethnicity predict greater vulnerability to antipsychotic-induced metabolic dysregulation, suggesting an overlap between risk factors for metabolic disease in the general population and in people with antipsychotic-induced metabolic disease. We did not observe a relationship between baseline weight and magnitude of antipsychotic-induced weight gain, as observed in some but not all previous studies.^33^ The discrepancies between our results with some of those previously documented might be a consequence of the large sample size used in our study, and the restriction of our analyses to randomised controlled trials of acute treatment (with previous studies examining weight gain over prolonged time periods of up to 3 years).^33^ We also showed that improvements in total symptom severity are associated with increases in weight, BMI, total cholesterol and LDL cholesterol concentrations, and decreases in HDL cholesterol concentrations, suggesting that the most efficacious antipsychotics are associated with the greatest metabolic disturbance. An alternative explanation is that metabolic side-effects of antipsychotics are similar and that our findings reflect medication compliance, with poor medication concordance in some participants resulting in a reduction in drug efficacy but also fewer metabolic side-effects. If so, perhaps previous trial reports of reduced metabolic side-effects of some antipsychotic treatments such as aripiprazole were not due to the pharmacological properties of the drug, rather to the fact that patients did not take the medication. However, when we examined data for metabolic changes with aripiprazole when concordance was assured via the use of long-acting injectable formulation, glucose and lipid alterations with aripiprazole treatment were no different from placebo treatment, generally in keeping with our findings.^34^ Furthermore, the degree of metabolic dysregulation has been shown to vary markedly between antipsychotics in preclinical studies.^35^ Our results are also in line with the outcomes of previous studies suggesting that more efficacious antipsychotics such as olanzapine and clozapine are generally associated with weight gain,^6^ and for BMI and weight, our findings support the results from previous cohort studies regarding magnitude and direction of association.^7^, ^8^, ^9^ Our findings do not mean that metabolic disturbance is a requirement for efficacy, but do highlight that those drugs that are most efficacious tend to have the broadest pharmacology, and metabolic effects might be due to off-target actions.

We used strict inclusion criteria to obtain a homogenous sample. We found no evidence of inconsistency for network meta-analyses examining change in BMI, LDL cholesterol, and HDL cholesterol, supporting the robustness of these outcomes. However, we had some concerns regarding inconsistency in the network meta-analyses of triglycerides and glucose, and more important concerns for the network meta-analysis of weight. These network meta-analyses showed evidence of global inconsistency, although only a small number of local hotspots of inconsistency. Inconsistency might have been secondary to imbalances in the distribution of some effect modifiers observed across comparisons and small study effects and publication bias that were noted in pairwise meta-analyses. Only a small proportion of studies (16%) showed no evidence of bias, and confidence in the evidence of the comparisons across all parameters was low or very low for 50–100% of treatment comparisons. Notably, the most recent and largest network meta-analysis examining comparative treatment efficacy of various antipsychotics identified the same issue, with confidence of outcomes for 75% of treatment-comparisons regarded as low or very low.^6^ However, our sensitivity analyses excluding patients with first-episode psychosis, treatment-resistant psychosis, older adults, and low-quality studies found similar results to the overall findings, and measures of inconsistency were largely unchanged, supporting the inclusion of these data in primary analyses.

In the general population, for every kg increase in body weight, cardiovascular disease risk increases by 3·1%,^36^ and for every kg/m^2^ increase in BMI, risk of heart failure increases by 5–7%,^37^ and risk of type 2 diabetes increases by 8·4%.^38^ Furthermore, a 1 mmol/L increase in triglyceride concentrations corresponds to a 32–76% increased risk of cardiovascular disease.^39^ Thus, around 6 weeks of treatment with antipsychotics such as olanzapine and clozapine, which increase body weight by approximately 3 kg, BMI by approximately 1 kg/m^2^, and triglycerides by approximately 1 mmol/L, might lead to important increases in cardiovascular disease risk. Hypertriglyceridemia accompanies the development of type 2 diabetes,^40^ and we observed increases in fasting glucose of 1 mmol/L with clozapine. At the onset of psychotic illness and before antipsychotic prescription, patients with schizophrenia have impaired glucose and lipid regulation.^41^, ^42^, ^43^ Thus, certain antipsychotics, within a few weeks, might worsen metabolic homeostasis in an already susceptible cohort, which reinforces international recommendations that metabolic monitoring should accompany antipsychotic prescription.^38^ By contrast, aripiprazole was the only antipsychotic to show across all parameters either no evidence of change or improvement in metabolic parameter levels compared with placebo. Brexpiprazole, cariprazine, and lurasidone showed improvements in some metabolic parameters compared with placebo. The metabolic effects of ziprasidone showed no clear difference compared with placebo for all parameters assessed. Given the risks of cardiovascular and other morbidity associated with metabolic dysregulation, these data should be used by clinicians and patients as one factor in the choice of an antipsychotic, particularly for people with risk factors such as increased body weight, being male, and non-white that we found predicted increased metabolic dysregulation. However, other side-effects should be considered, such as extrapyramidal side-effects, 6, 13 noting that differences exist in efficacy between drugs,^6^ which should also be factored when choosing treatment.

Our findings should also be considered in the context of population-based studies showing that patients with schizophrenia who receive antipsychotic treatment, especially clozapine, have lower all-cause and cardiac mortality rates compared with patients who do not receive antipsychotic treatment.^44^ Our observation that symptomatic improvement accompanies metabolic dysregulation might provide some insight into why, paradoxically, cardiovascular mortality improves with treatments that lead to worse metabolic outcomes. Improvements in mental state might result in improved self-care and engagement with physical health services, which might offset the metabolic risk of a drug. Clozapine and olanzapine are among the most effective antipsychotic drugs and are also the drugs associated with the highest risk of metabolic dysregulation.^6^ Whether the association between symptom improvement and metabolic dysregulation reflects an intrinsic therapeutic link is unclear. One possible explanation is that the antipsychotic receptor binding profiles implicated in metabolic dysregulation, such as serotonin 5-HT_2A_, histamine H_1_, and muscarinic M_3_ receptors,^13^ also play a therapeutic role alongside D_2_ dopamine receptor blockade.^38^ In addition to serotonin, histamine, and muscarinic activity, peripheral dopaminergic signalling might play a role in defining the metabolic profiles associated with different antipsychotics, which could explain the various lipid and glucose outcomes associated with dopamine receptor antagonists compared with partial agonists. However, the central and peripheral mechanisms that underlie the effects of antipsychotic drugs on metabolic parameters are poorly understood. Future pre-clinical work should explore whether peripheral receptor binding profiles of different antipsychotics explain the drugs' respective metabolic signatures, and whether this can be manipulated to mitigate the metabolic side-effects of treatment.

Our analysis had some limitations. Despite attempts made to contact authors, we were unable to obtain metabolic data for several trials, especially if the study was done more than 15 years ago. Thus, our findings are mostly restricted to randomised controlled trials of recently licensed antipsychotics. Further work is required to define the metabolic profiles of older drugs, which will better inform prescribing practice. We restricted our analyses to randomised controlled trials so that biases were controlled for to give the best estimates of drug-specific effects. However, because randomised controlled trials are generally quite short, the duration of treatment in the studies included was in the range 2–13 weeks. Future network meta-analyses should examine antipsychotic-induced metabolic dysregulation in patients receiving long-term maintenance therapy. Studies often did not report on lifestyle and treatment factors that might influence metabolic outcomes, including physical comorbidity, alcohol use, smoking, diet, exercise, and co-prescription of psychiatric (eg, mood stabilisers) or physical health medications (eg, statins or anti-glycaemic drugs) that might have influenced metabolic parameters. However, randomisation of participants should have distributed study participants with these confounders equally between groups. Our meta-regression analyses were based on study-level data and require replication with individual patient data. Studies included in the meta-analysis often did not report the proportions of various non-white ethnic groups; therefore, we were unable to examine in greater detail the influence of various ethnicities on metabolic outcomes.

In conclusion, marked variations exist in the metabolic side-effects of antipsychotics, with olanzapine and clozapine showing the worst side-effect profiles. Aripiprazole, brexpiprazole, cariprazine, lurasidone, and ziprasidone are associated with the best metabolic outcomes and these drugs can be considered the safest options in those at an increased risk of developing metabolic complications. However, clinical decisions to use preferentially antipsychotics with fewer metabolic side-effects should consider that clinical improvement is associated with development of these side-effects. We identified increased baseline weight, being male, and non-white as potential risk factors for antipsychotic-induced metabolic disturbance. Treatment guidelines should be updated to reflect differences in metabolic risk, but the choice of the treatment intervention should be made on an individual patient basis, considering the clinical circumstances and preferences of patients, carers, and clinicians.
