# Supplementary material for: Comparative effects of 18 antipsychotics on metabolic function in patients with schizophrenia, predictors of metabolic dysregulation, and association with psychopathology: a systematic review and network meta-analysis
Source: Lancet Psychiatry. 2020 Jan;7(1):64–77. doi: 10.1016/S2215-0366(19)30416-X (PMC7029416; doi:10.1016/S2215-0366(19)30416-X)
Supplement: Supplementary appendix [file mmc1.pdf]

THE LANCET
Psychiatry

Supplementary appendix

This appendix formed part of the original submission and has been peer reviewed. We post it as supplied by the authors.

Supplement to: Pillinger T, McCutcheon RA, Vano L, et al. Comparative effects of 18 antipsychotics on metabolic function in schizophrenia, predictors of metabolic dysregulation, and association with psychopathology: a systematic review and network meta-analysis. *Lancet Psychiatry* 2019; published online Dec 17. https://doi.org/10.1016/S2215-0366(19)30416-X.

SUPPLEMENTARY INFORMATION

Comparative effects of 18 antipsychotics on metabolic function in schizophrenia, predictors of metabolic dysregulation, and association with psychopathology: a systematic review and network meta-analysis

Pillinger T, McCutcheon RA, Vano, L, Mizuno Y, Beck K, Arumuhan A, Hindley G, Natesan S, Efthimiou O, Cipriani A, Howes OD, 2019

CONTENTS

**Pages 2-4**	**eTable 1.** PRISMA NMA checklist
**Pages 5-18**	**eAppendix 1.** PROSPERO registration and protocol
**Page 19**	**eAppendix 2.** Methods to assess confidence in NMA using CINeMA
**Page 20**	**eFigure 1.** Literature search process
**Pages 21-33**	**eTable 2.** Study characteristics
**Pages 34-35**	**eTable 3.** Cochrane risk of bias assessment
**Page 36**	**eTable 4.** Baseline parameters across treatment-comparisons
**Page 37**	**eAppendix 3.** Pairwise meta-analyses
**Pages 37-38**	**eFigures 5-7.** Pairwise meta-analysis forest plots
**Page 39-40**	**eFigures 8-10.** Pairwise meta-analysis funnel plots
**Pages 41-46**	**eTables 5-11.** League tables comparing metabolic alterations associated with antipsychotic drug treatment.
**Pages 47-49**	**eTables 12-18.** P-score ranking for metabolic alterations associated with antipsychotic drug treatment.
**Pages 50-61**	**eTables 19-25.** Identifying 'hot spots' of inconsistency for the 7 network meta-analyses
**Pages 62-87**	**eTables 26-32.** CINeMA confidence ratings for the drug comparisons making up the 7 network meta-analyses.
**Pages 88-92**	**eAppendix 4.** Post hoc sensitivity analysis for antipsychotic-induced weight gain (low quality studies removed)
**Page 93**	**eAppendix 5.** Sensitivity analyses
**Pages 94-97**	**eFigures 11-17.** Forest plots (compared with placebo) for the 7 sensitivity network meta-analyses.
**Pages 98-105**	**Supplementary References**

eTable 1. PRISMA NMA Checklist

Section/Topic	Item #	Checklist Item	Reported on Page #
**TITLE**			
Title	1	Identify the report as a systematic review *incorporating a network meta-analysis (or related form of meta-analysis).*	**1**
**ABSTRACT**			
Structured summary	2	Provide a structured summary including, as applicable:<br>**Background:** main objectives<br>**Methods:** data sources; study eligibility criteria, participants, and interventions; study appraisal; and *synthesis methods, such as network meta-analysis.*<br>**Results:** number of studies and participants identified; summary estimates with corresponding confidence/credible intervals; *treatment rankings may also be discussed. Authors may choose to summarize pairwise comparisons against a chosen treatment included in their analyses for brevity.*<br>**Discussion/Conclusions:** limitations; conclusions and implications of findings.<br>**Other:** primary source of funding; systematic review registration number with registry name.	**3**
**INTRODUCTION**			
Rationale	3	Describe the rationale for the review in the context of what is already known, *including mention of why a network meta-analysis has been conducted.*	**4**
Objectives	4	Provide an explicit statement of questions being addressed, with reference to participants, interventions, comparisons, outcomes, and study design (PICOS).	**4**
**METHODS**			
Protocol and registration	5	Indicate whether a review protocol exists and if and where it can be accessed (e.g., Web address); and, if available, provide registration information, including registration number.	**5**
Eligibility criteria	6	Specify study characteristics (e.g., PICOS, length of follow-up) and report characteristics (e.g., years considered, language, publication status) used as criteria for eligibility, giving rationale. *Clearly describe eligible treatments included in the treatment network, and note whether any have been clustered or merged into the same node (with justification).*	**5&6**
Information sources	7	Describe all information sources (e.g., databases with dates of coverage, contact with study authors to identify additional studies) in the search and date last searched.	**5**
Search	8	Present full electronic search strategy for at least one database, including any limits used, such that it could be repeated.	**Sup Info**
Study selection	9	State the process for selecting studies (i.e., screening, eligibility, included in systematic review, and, if applicable, included in the meta-analysis).	**5&6**
Data collection process	10	Describe method of data extraction from reports (e.g., piloted forms, independently, in duplicate) and any processes for obtaining and	**5&6**

		confirming data from investigators.	
Data items	11	List and define all variables for which data were sought (e.g., PICOS, funding sources) and any assumptions and simplifications made.	**5&6**
**Geometry of the network**	**S1**	Describe methods used to explore the geometry of the treatment network under study and potential biases related to it. This should include how the evidence base has been graphically summarized for presentation, and what characteristics were compiled and used to describe the evidence base to readers.	**6**
Risk of bias within individual studies	12	Describe methods used for assessing risk of bias of individual studies (including specification of whether this was done at the study or outcome level), and how this information is to be used in any data synthesis.	**7**
Summary measures	13	State the principal summary measures (e.g., risk ratio, difference in means). *Also describe the use of additional summary measures assessed, such as treatment rankings and surface under the cumulative ranking curve (SUCRA) values, as well as modified approaches used to present summary findings from meta-analyses.*	**6&7**
Planned methods of analysis	14	Describe the methods of handling data and combining results of studies for each network meta-analysis. This should include, but not be limited to:<br>• *Handling of multi-arm trials;*<br>• *Selection of variance structure;*<br>• *Selection of prior distributions in Bayesian analyses; and*<br>• *Assessment of model fit.*	**5-7**
**Assessment of Inconsistency**	**S2**	Describe the statistical methods used to evaluate the agreement of direct and indirect evidence in the treatment network(s) studied. Describe efforts taken to address its presence when found.	**7**
Risk of bias across studies	15	Specify any assessment of risk of bias that may affect the cumulative evidence (e.g., publication bias, selective reporting within studies).	**7**
Additional analyses	16	Describe methods of additional analyses if done, indicating which were pre-specified. This may include, but not be limited to, the following:<br>• Sensitivity or subgroup analyses;<br>• Meta-regression analyses;<br>• *Alternative formulations of the treatment network; and*<br>• *Use of alternative prior distributions for Bayesian analyses (if applicable).*	**7&8**
**RESULTS**			
Study selection	17	Give numbers of studies screened, assessed for eligibility, and included in the review, with reasons for exclusions at each stage, ideally with a flow diagram.	**8**
**Presentation of network structure**	**S3**	Provide a network graph of the included studies to enable visualization of the geometry of the treatment network.	**Fig 1**
**Summary of network geometry**	**S4**	Provide a brief overview of characteristics of the treatment network. This may include commentary on the abundance of trials and randomized patients for the different interventions and pairwise comparisons in the network, gaps of evidence in the treatment network, and potential biases reflected by the network structure.	**8**
Study characteristics	18	For each study, present characteristics for which data were extracted (e.g., study size, PICOS, follow-up period) and provide the citations.	**eTable 2**

Risk of bias within studies	19	Present data on risk of bias of each study and, if available, any outcome level assessment.	**eTable 3**
Results of individual studies	20	For all outcomes considered (benefits or harms), present, for each study: 1) simple summary data for each intervention group, and 2) effect estimates and confidence intervals. *Modified approaches may be needed to deal with information from larger networks.*	**eTable 2**
Synthesis of results	21	Present results of each meta-analysis done, including confidence/credible intervals. *In larger networks, authors may focus on comparisons versus a particular comparator (e.g. placebo or standard care), with full findings presented in an appendix. League tables and forest plots may be considered to summarize pairwise comparisons.* If additional summary measures were explored (such as treatment rankings), these should also be presented.	**Fig 2 eTables 4-10**
**Exploration for inconsistency**	**S5**	Describe results from investigations of inconsistency. This may include such information as measures of model fit to compare consistency and inconsistency models, *P* values from statistical tests, or summary of inconsistency estimates from different parts of the treatment network.	**9-12**
Risk of bias across studies	22	Present results of any assessment of risk of bias across studies for the evidence base being studied.	**Figure 2 eTable 3 eTables 25-31**
Results of additional analyses	23	Give results of additional analyses, if done (e.g., sensitivity or subgroup analyses, meta-regression analyses*, alternative network geometries studied, alternative choice of prior distributions for Bayesian analyses,* and so forth).	**12**
**DISCUSSION**			
Summary of evidence	24	Summarize the main findings, including the strength of evidence for each main outcome; consider their relevance to key groups (e.g., healthcare providers, users, and policy-makers).	**13**
Limitations	25	Discuss limitations at study and outcome level (e.g., risk of bias), and at review level (e.g., incomplete retrieval of identified research, reporting bias). *Comment on the validity of the assumptions, such as transitivity and consistency. Comment on any concerns regarding network geometry (e.g., avoidance of certain comparisons).*	**16**
Conclusions	26	Provide a general interpretation of the results in the context of other evidence, and implications for future research.	**16-17**
**FUNDING**			
Funding	27	Describe sources of funding for the systematic review and other support (e.g., supply of data); role of funders for the systematic review. This should also include information regarding whether funding has been received from manufacturers of treatments in the network and/or whether some of the authors are content experts with professional conflicts of interest that could affect use of treatments in the network.	**19**

eAppendix 1. PROSPERO Registration

Registration number: CRD42019125322

Available online: https://www.crd.york.ac.uk/prospero/display_record.php?RecordID=125322

******PROTOCOL******

Review questions

1. What is the magnitude of metabolic dysregulation (defined as alterations in fasting glucose, total cholesterol, low density lipoprotein (LDL) cholesterol, high density lipoprotein (HDL) cholesterol, and triglyceride levels) and alterations in body weight and body mass index associated with short-term ('acute') antipsychotic treatment in individuals with schizophrenia?
2. Does baseline physiology (e.g. body weight) and demographics (e.g. age) of patients predict magnitude of antipsychotic-associated metabolic dysregulation?
3. Are alterations in metabolic parameters over time associated with alterations in degree of psychopathology?

Searches

We plan to search EMBASE, PsycINFO, and MEDLINE from inception using the following terms:

1

(Acepromazine or Acetophenazine or Amisulpride or Aripiprazole or Asenapine or Benperidol or Blonanserin or Bromperidol or Butaperazine or Carpipramine or Chlorproethazine or Chlorpromazine or Chlorprothixene or Clocapramine or

Clopenthixol or Clopentixol or Clothiapine or Clotiapine or Clozapine or Cyamemazine or Cyamepromazine or Dixyrazine or Droperidol or Fluanisone or Flupehenazine or Flupenthixol or Flupentixol or Fluphenazine or Fluspirilen or Fluspirilene or Haloperidol or Iloperidone or Levomepromazine or Levosulpiride or Lithium or Loxapine or Loxapinsuccinate or Lurasidone or Melperone or Mepazine or Mesoridazine or Methotrimeprazine or Molindone or Moperone or Mosapramine or Olanzapine or Oxypertine or Paliperidone or Penfluridol or Perazine or Periciazine or Pericyazine or Perospirone or Perphenazine or Pimozide or Pipamperone or Pipothiazine or Pipotiazine or Prochlorperazine or Promazine or Promethazine or Prothipendyl or Quetiapine or Remoxipiride or Reserpine or Riospirone or Risperdal or Risperidone or Seroquel or Sertindole or Stelazine or Sulpiride or Sultopride or Thiopropazate or Thioproperazine or Thioridazine or Tiospirone or Thiothixene or Tiapride or Tiotixene or Trifluoperazine or Trifluperidol or trifluoperidol or Triflupromazine or trifluperazine or Veralipride or Ziprasidone or Zotepine or Zuclopenthixol).mp. [mp=ti, ab, hw, tn, ot, dm, mf, dv, kw, fx, dq, nm, kf, ox, px, rx, ui, sy, tc, id, tm]

2

(Antipsychoti$ or Anti-psychotic$ or Neurolepic$ or Neurolept$).mp. [mp=ti, ab, hw, tn, ot, dm, mf, dv, kw, fx, dq, nm, kf, ox, px, rx, ui, sy, tc, id, tm]

3

schizo$.mp.

4

psychosis.mp.

5

randomized.mp. [mp=ti, ab, hw, tn, ot, dm, mf, dv, kw, fx, dq, nm, kf, ox, px, rx, an, ui, sy, tc, id, tm]

6

'double blind'.mp. [mp=ti, ab, hw, tn, ot, dm, mf, dv, kw, fx, dq, nm, kf, ox, px, rx, an, ui, sy, tc, id, tm]

7

5 and 6

8

3 or 4

9

1 or 2

10

7 and 8 and 9

Types of study to be included

Double-blind, randomised controlled trials that have been published in the English language. Clinical trials registry data relating to papers identified in the literature review will also be included.

Condition or domain being studied

Metabolic alterations (glucose, total cholesterol, Low Density Lipoprotein (LDL) cholesterol, High Density Lipoprotein (HDL) cholesterol, and triglyceride levels), body weight, body mass index.

Participants/population

Patients with schizophrenia and related psychoses defined according to standard operationalised diagnostic criteria (Feighner criteria, Research Diagnostic Criteria, DSM-III, DSM-III-R, DSM-IV, DSM-V, and ICD-10).

Intervention(s), exposure(s)

Monotherapy with antipsychotic or placebo. We will not employ limits on antipsychotic dose, owing to the lack of clear evidence that antipsychotic treatment dose influences degree of metabolic dysregulation, although we will investigate the potential influence of antipsychotic dose on metabolic change using meta-regression (see 'Meta-regression Analyses' section).[1] Oral or parenteral administration will be accepted.

Comparator(s)/control

Monotherapy with antipsychotic or placebo.

Context

It has long been proposed that some antipsychotic drug treatments cause glucose dysregulation and lipid disturbance thereby contributing to development of the metabolic syndrome in patients with schizophrenia.[2] However, the relative degree to which metabolic alterations occur in acute treatment with different antipsychotics remain unclear. Furthermore, baseline predictors of metabolic dysregulation are poorly defined, and the association between metabolic change and change in psychopathology is uncertain.

Main outcome(s)

For each study, we aim to collect data examining mean and standard deviation of change (i.e. from baseline to study endpoint) in the following outcomes:

1. Glucose (mmol/L)
2. Total cholesterol (mmol/L)
3. Low Density Lipoprotein (LDL) cholesterol (mmol/L)
4. High Density Lipoprotein (HDL) cholesterol (mmol/L)
5. Triglycerides (mmol/L)
6. Body weight (kg)
7. Body Mass Index (BMI, kg/m^2)

All metabolic outcomes will be measured from blood tests taken under fasting conditions. Either plasma or serum samples will be accepted.

Data extraction (selection and coding)

This systematic review and network meta-analysis will adhere to recommendations of the Preferred Reporting Items for Systematic Reviews and Meta-analyses (PRISMA)[3] extension statement for network meta-analysis.

Extracted information will include: name of first author, year of publication, antipsychotic used in study, average dose of antipsychotic used, type of symptom scale used, patient characteristics including age, %male, %Caucasian, duration of drug intervention, mean ± standard deviation (SD) change in symptom scores between baseline and study endpoint, mean ± SD metabolic parameter concentrations (glucose, total/LDL/HDL cholesterol/triglycerides) and body weight/body mass index (BMI) at baseline, active drug number, placebo number.

If applicable, data may be extracted from related publications that refer to the same study. When data required for meta-analysis is unreported, corresponding authors will be contacted to request additional data.

Network meta-analysis requires reasonable homogeneity, and as such we will focus on acute treatment, which we define as 6-weeks duration.[4] If 6-week data are not available, the datapoint closest to 6-weeks will be given preference. Again, to maintain homogeneity in the sample, we will exclude paediatric studies (i.e. studies where participant age <18 years). We will not employ limits on antipsychotic dose, owing to the lack of clear evidence that antipsychotic dose influences degree of metabolic dysregulation, although we will investigate the potential influence of antipsychotic dose on metabolic change using meta-regression (see 'Strategy for Data Synthesis' section).[1] Where multiple doses of a single antipsychotic are reported, to increase statistical power, and again in the absence of clear evidence that antipsychotic dose

influences metabolic outcomes, a single weighted mean and standard deviation for each metabolic parameter pertaining to a given multi-arm study will be calculated, using formulae recommended by the Cochrane collaboration:[5]

Weighted mean of multiple study arms:

$$\bar{x} = \frac{\sum_{i=1}^{N} n_i x_i}{\sum_{i=1}^{N} n_i}$$

Where,

N= number of observations (i.e. number of arms of the study)

n_i = sample size of study arm

x_i = sample mean of study arm

Weighted standard deviation for 2 study arms:

$$s = \sqrt{\frac{(n_1 - 1)sd_1^2 + (n_2 - 1)sd_2^2 + \frac{n_1 n_2}{n_1 + n_2}(x_1^2 + x_2^2 - 2x_1 x_2)}{n_1 + n_2 - 1}}$$

Where,

n_1 and n_2 = sample sizes of study arms 1 and 2

x_1 and x_2 = means of study arms 1 and 2

sd_1 and sd_2 = standard deviations of study arms 1 and 2

As recommended by Cochrane,[5] where there are more than 2 study arms to combine standard deviations, the above formula will be applied sequentially (i.e. combining study arm 1 and 2 to create arm ‘1+2’, then combining group ‘1+2’ and group 3 to create group ‘1+2+3’ and so on).

Since paliperidone represents the active metabolite of risperidone, data pertaining to these 2 antipsychotics will be merged (i.e. considered as the same intervention). Since we set out to examine mean difference between groups (see 'Strategy for Data Synthesis' section), data from certain studies will require conversion from conventional to SI units, which will be performed using standardised conversion factors.[6]

Clinical trials registry data relating to papers identified in the literature review will be included.

5 researchers (YM/LV/KB/AA/GH) will extract data, all studies will be assessed by a minimum of two researchers.

Discrepancies will be decided by TP.

Strategy for data synthesis

Characteristics of included studies

We will describe the study population characteristics across all eligible trials, describing the types of comparisons (i.e. which metabolic parameter is examined), and physiological/demographic variables (including age, gender (%male), ethnicity (%Caucasian), duration of drug intervention, and clinical group (i.e. first episode psychosis, established schizophrenia, treatment resistant schizophrenia, older adults).

Pairwise meta-analyses

For each pairwise comparison with ≥10 studies we will synthesise data to obtain summary mean differences with accompanying 95% confidence interval using a random effects model. Analyses will be carried out in the statistical programming language R (version 3.5.1)[7] using 'metafor' (version 2.1-0). Visual inspection of the forest plots will be used to investigate the degree of statistical heterogeneity, alongside

monitoring of τ (the estimated standard deviation of random effects) and the I^2 statistic. An I^2 of less than 25% was deemed to have low heterogeneity, 25-75% medium heterogeneity, and greater than 75% high heterogeneity. To help visualize the extent of heterogeneity we will also include prediction intervals in all forest-plots.

Small study effects and publication bias will be assessed for each pairwise comparison by visual inspection of the contour-enhanced funnel plot and by performing Egger's test of the intercept.[8]

Assessment of the transitivity assumption

In an attempt to ensure transitivity in the network, we will restrict analyses to patients with schizophrenia and related psychoses (i.e. we will not examine the metabolic effects of antipsychotics in other patient groups), we will exclude studies examining paediatric patients, and we will restrict analyses to studies that only examine acute treatment (aiming for treatment duration of 6-weeks).[9]

Potential effect modifiers include age, gender, and ethnicity, as these parameters are known in the general population to influence metabolic change.[10-12] As such, we will examine if age, gender (% male) and ethnicity (% Caucasian) of participants is similarly distributed across the different treatment comparisons.

Network Analyses

If the collected studies appear to be sufficiently similar with respect to the distribution of age, gender, and ethnicity, we will conduct a random effects network meta-analysis (NMA) to synthesise all evidence for each outcome. We will use a frequentist approach to NMA using 'netmeta' (version 1.0-1).[13,14] Network plots will be generated using the 'netgraph' function. In line with previous meta-analyses in the field[15,16] and to allow

intuitive clinical interpretation of results, metabolic change for each given parameter will be expressed as the mean difference (MD) with 95% confidence intervals. MD preserves original units, thus, for example, when examining difference in weight between 2 groups, a calculated MD of 2 equates to a 2kg difference. Placebo will be selected as the reference 'treatment' and forest plots created using 'ggplot2' (version 2.2.1). League tables will be created to display the relative degree of metabolic disturbance for all antipsychotics using the 'netleague' function. For each metabolic parameter, a frequentist analogue of 'Surface under the Cumulative Ranking Curve' (SUCRA) will be used to rank antipsychotics based on degree of metabolic dysregulation, using the 'netrank' function. This provides P-scores which rank antipsychotics on a continuous 0 to 1 scale: a higher P-score indicates greater degree of metabolic disturbance.

Assessments of heterogeneity and inconsistency

Heterogeneity of each network will be assessed by monitoring of τ and the I^2 statistic. Consistency of each network (i.e. the agreement between direct and indirect evidence) will be evaluated using global (Q statistic) and local methods (identifying 'hot spots' of inconsistency using the 'netsplit' function).[17,18]

Sensitivity analyses

We hypothesize that inclusion of different study populations (i.e. first episode psychosis (FEP), treatment responders, treatment resistant schizophrenia (TRS), and older adults) may contribute to heterogeneity and inconsistency. As such, the sensitivity of our findings for all 7 outcomes will be evaluated by repeating each NMA with the exclusion of studies that examine FEP, TRS, or older adults.

Meta-regression analyses

In the general population it is known that body weight/BMI,[19] age,[10] gender,[11] and ethnicity[12] influence metabolic function. We will investigate if these covariates as well as treatment factors are related to change in metabolic parameters. We will perform a meta-regression of placebo-controlled data to examine the relationship between antipsychotic-associated metabolic changes and baseline body weight, BMI, baseline level of a given parameter (e.g. baseline glucose levels if examining change in glucose), age, gender (% male), ethnicity (% non-Caucasian), and olanzapine-equivalent dose (calculated using previously defined dose equivalents).[20,21] Since any associations between baseline parameters and antipsychotic associated metabolic change may be influenced by the type of antipsychotic a certain group may have been prescribed, we will repeat all meta-regression analyses including each different antipsychotic as a moderator. All meta-regression will be performed using 'metafor' (version 2.0.0) and representative plots generated using 'ggplot2' (version 2.2.1).

Measurement of strength of relationship between alterations in body weight/BMI/metabolic parameter and alterations in psychopathology

The association between metabolic change and change in psychopathology is uncertain. As such, for placebo-controlled data, we will perform a bivariate meta-analysis, aiming to calculate the correlation coefficient between the effect size (standardised mean difference, SMD) for change in a given metabolic parameter/weight/BMI and the effect size (SMD) for change in total symptoms (assessed using the Positive and and Negative Syndrome Scale[17] or Brief Psychiatric Rating Scale scores).[18] This will be performed using the bivariate meta-analysis model

proposed by Riley and colleagues.[22] Analysis will be performed using the package 'metamisc' (version 0.2.0).

Risk of bias (quality) assessment

Risk of bias of individual studies will be assessed using the Cochrane Collaboration's Tool for Assessing Risk of Bias.[23] The following domains will be considered: randomization sequence generation; allocation concealment; blinding of participants/trial personnel/outcome assessment; incomplete outcome data; and selective outcome reporting.

The 'Confidence in Network Meta-Analysis' (CINeMA)[24,25] application will be employed to evaluate the credibility of findings from each network meta-analysis. As part of the CINeMA evaluation process, a risk of bias assessment is required for each study with each study categorised as at low, unclear, or high risk of bias. We will convert our Cochrane Risk of Bias Assessment Tool ratings into CINeMA categories as follows:

**Cochrane Risk of Bias Assessment Tool Rating**	**CINeMA risk of bias category**
All domains categorised as low risk	Low risk (1)
All domains categorised as either low risk or unclear risk	Unclear/moderate risk (2)
Any domain categorised as high risk	High risk (3)

Contact details for further information

Toby Pillinger

toby.pillinger@kcl.ac.uk

Organisational affiliation of the review

King's College London

University of Oxford

University of Bern

Review team members and their organisational affiliations

Dr Toby Pillinger. King's College London

Dr Robert McCutcheon. King's College London

Dr Katherine Beck. King's College London

Dr Guy Hindley. King's College London

Dr Atheeshaan Arumuham. King's College London

Dr Luke Vano. King's College London

Dr Yuya Mizuno. King's College London

Dr Sridhar Natesan. King's College London

Dr Orestis Efthimiou. University of Bern

Professor Andrea Cipriani. University of Oxford

Professor Oliver Howes. King's College London

Type and method of review

Meta-analysis, Systematic review

Funding sources/sponsors

ODH is supported by MC-A656-5QD30 from the Medical Research Council-UK, 666 from the Maudsley Charity, 094849/Z/10/Z from the Brain and Behavior Research Foundation, and Wellcome Trust. RM is supported by the Wellcome Trust (no.

200102/Z/15/Z). AC is supported by the National Institute for Health Research (NIHR) Oxford Cognitive Health Clinical Research Facility, grant RP-2017-08-ST2-006 from NIHR Research Professorship, and grant BRC-1215-20005 from the NIHR Oxford Health Biomedical Research Centre. OE is supported by project grant No. 180083 from the Swiss National Science Foundation (SNSF).

Conflicts of interest

Professor Howes has received investigator-initiated research funding from and/or participated in advisory/speaker meetings organized by AstraZeneca, Autifony, BMS, Eli Lilly, Heptares, Janssen, Lundbeck, Lyden-Delta, Otsuka, Servier, Sunovion, Rand, and Roche. Drs Pillinger, McCutcheon, Vano, Mizuno, Efthimiou, and Cipriani report no conflicts of interest.

Subject index terms status

Subject indexing assigned by CRD

Subject index terms

Antipsychotic Agents; Humans; Network Meta-Analysis

Date of registration in PROSPERO

27 February 2019

eAppendix 2. Methods employed to assess confidence in NMA results using CINeMA

1. **Within-study bias**
   Defined as per Cochrane risk of bias assessment as follows:

Cochrane Risk of Bias Assessment Tool Rating	CINeMA risk of bias category
All domains categorised as low risk	Low risk (1)
Domains categorised as either low risk or unclear risk	Unclear/moderate risk (2)
Any domain categorised as high risk	High risk (3)

2. **Across-studies bias**
   Where pairwise meta-analyses are possible, grade risk of bias based on funnel plot/Egger's regression test. If <10 studies for a given pairwise comparison, downgrade confidence.
3. **Indirectness**
   No indirectness assumed
4. **Imprecision**
   A clinically meaningful threshold was set at a mean difference of higher or lower than 0.
5. **Heterogeneity**
   Use recommendations automatically provided by CINeMA
6. **Incoherence**
   Downgrade evidence if only indirect evidence

eFigure 1. Search process

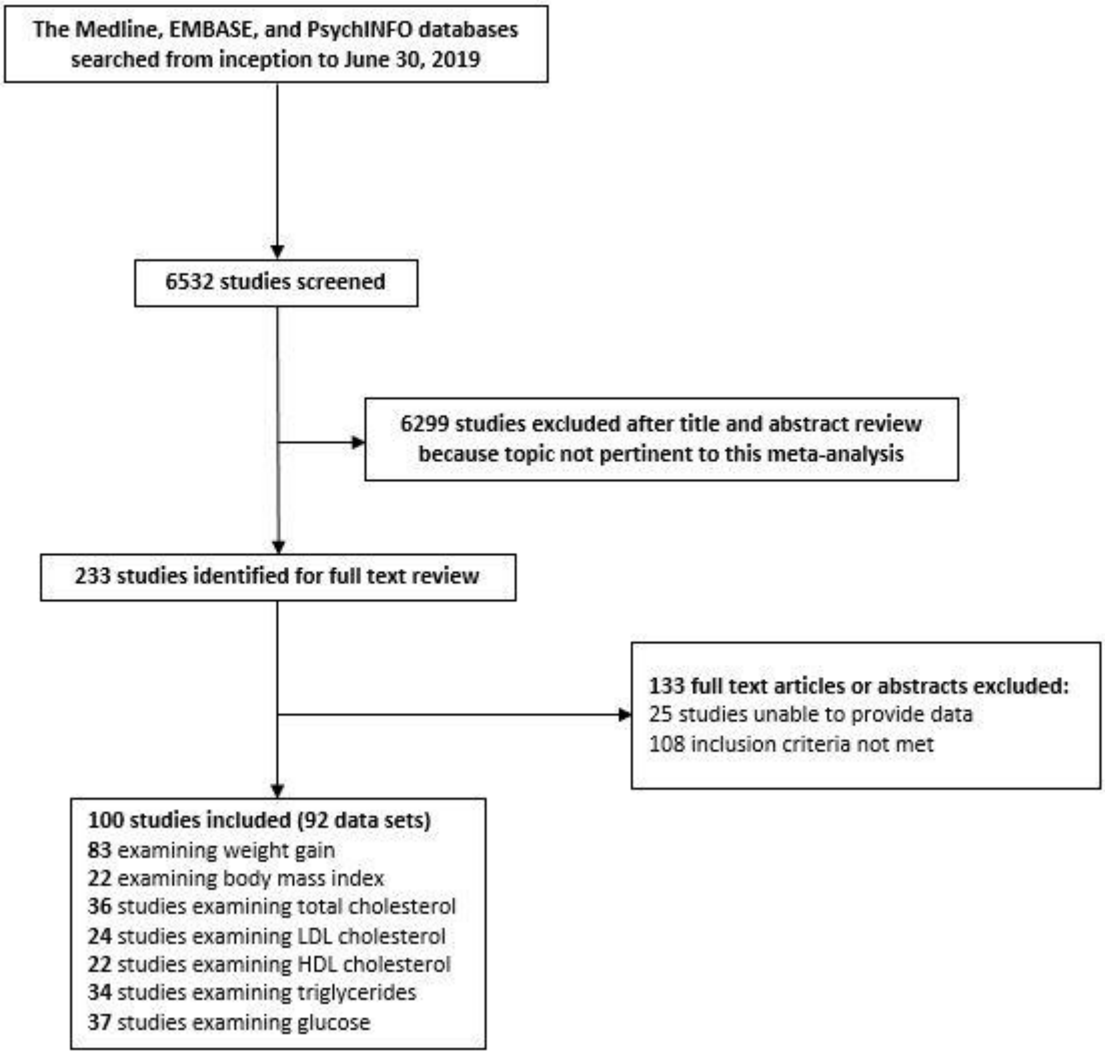

eTable 2. Studies included in meta-analysis.

Each row corresponds to a single data set, for which more than one study may correspond.

Study	Country, patient population	Physical Health Exclusion Criteria	Antipsychotic	Antipsychotic Dose (mg)	Trial Duration (weeks)	Antipsychotic (N)	Placebo (N)	Parameter
**Ascher-Svanum 2005**[26] **Beasley 1996**[27]	Not specifically stated, likely USA Schizophrenia Aged 18-65	'Serious unstable medical illness'	olanzapine	10	6	187	61	Weight
**Azorin 2006**[28]	France Schizophrenia Aged 18-65	QTc over 430 (M) or 450 (F)	risperidone<br>sertindole	6.6<br>16.2	12<br>12	61<br>59	N/A	Weight, BMI
**Baymiller 2002**[29]	Not specifically, stated, likely USA Schizophrenia or Schizoaffective disorder Age not stated, likely 18+	None stated	clozapine<br>haloperidol	200-600<br>10-30	10<br>10	28<br>33	N/A	Weight
**Bhowmick 2010**[30]	India Schizophrenia Aged 18-65	Pregnant, breast-feeding, neurological disease "significant disease of vital organs, adrenal or pituitary glands", receiving medication known to prolong QT or interact with study medication	amisulpride<br>olanzapine	450<br>15	12<br>12	39<br>38	N/A	Weight
**Beasley 1996**[31]	USA and Canada Schizophrenia with acute exacerbation Aged 18-65	Serious, unstable medical illness, neurological disease, use of anticholinergic medication, significantly elevated laboratory test results, active hepatitis B, or jaundice	Olanzapine	10	6	28	10	Weight
**Bossie, 2011**[32]	USA, Europe, and Asia Schizophrenia	'Unstable systemic	paliperidone	7.32	13	252	71	Weight

	Aged 18+<br>Illness for at least 1yr	disease…BMI ≥40kg/m2'						
**Breier 2005**[33]	Europe and North and South America<br>Schizophrenia<br>Aged 18-75	Congenital long QT syndrome, a QTc interval longer than 500, any ECG abnormality that would confound measurement of the QT interval, serious unstable mental illness	olanzapine<br>ziprasidone	15.27<br>115.96	4<br>4	165<br>115	N/A	Weight, FG, TG, HDL, LDL, TC
**Buckley 2004**[34]	Not specifically stated, likely USA and South Africa<br>Schizophrenia<br>Aged 18+	Known sensitivity to drugs used in trial, history of idiopathic or drug-induced agranulocytosis	quetiapine<br>haloperidol	600<br>20	4<br>4	39<br>32	N/A	Weight
**Cantillon 2017**[35]	India, Philippines, Malaysia, Moldova and USA<br>Schizophrenia or schizoaffective disorder<br>Age 18-65,	None stated	aripiprazole	15	4	Weight/BMI: 14<br>FG, TC, TG: 18	38	Weight, BMI, FG, TC, TG
**Canuso 2009**[36]	India, Russia, Ukraine, USA<br>Schizophrenia<br>Aged 18-65	None stated	paliperidone<br>quetiapine	PAL: 10.4<br>QUET: 690.9	6	124,106	51	Weight
**Canuso 2010a**[37]	India, Russia, Ukraine, USA<br>Schizophrenia<br>Schizoaffective disorder<br>Aged 18-65	None stated	paliperidone	12, 6	6	98, 108	107	Weight, FG, LDL, HDL, TG
**Canuso 2010b**[38]	United States, Romania, India, Philippines, Malaysia, and South Korea<br>Schizoaffective disorder<br>Aged 18-65	None stated	paliperidone	8.6	6	134	53	Weight, FG, TC, LDL, HDL, TG
**Canuso 2010**[39]	Not stated<br>Schizophrenia<br>Aged 18+	Poor general health or chronic health conditions	paliperidone	8.1	6	116	37	Weight
**Conley 2001**[40]	USA<br>Schizophrenia or schizoaffective disorder<br>Aged 18-64	Neurological disease, use of disallowed concomitant therapy (e.g.	risperidone<br>olanzapine	4.8<br>12.4	8<br>8	146<br>135	N/A	Weight, BMI

		mood stabilisers or antidepressants)						
**Conley 2005**[41]	Not specifically stated, likely USA Treatment resistant schizophrenia Aged 18-65	Use of routine concomitant medications with CNS activity (e.g. antidepressants, mood stabilisers)	risperidone<br>quetiapine	4<br>400	12<br>12	13<br>12	N/A	Weight
**Coppola 2011**[42]	United states, India, Taiwan Schizophrenia Aged 18+	Any medical condition that could potentially alter the absorption, metabolism, or excretion of the study drug (e.g., Crohn's, liver, or renal disease)	paliperidone	1.5, 6	6	Weight: 58,64<br>FG: 55, 60	56	Weight, FG
**Correll 2016**[43] **(Correll 2015**[44] **NCT00905307 NCT01396421 NCT01393613)**	United States, Ukraine, Romania, Serbia, Latvia, Malaysia, Japan, Poland, South Korea, Canada Schizophrenia Aged 18-65	'A clinically significant medical condition'	brexpiprazole, aripiprazole	BREX: 0.25, 1, 2, 4<br>ARI: 15	6	41, 88, 90, 92, 50	93	Weight, FG, TC, LDL, HDL, TG
			brexpiprazole	BREX: 0.25, 1, 2, 4	6	87, 117,359, 359	358	
**Cutler 2011**[45]	United States Schizophrenia Aged 18-65	'Any significant diseases (e.g. renal and hepatic impairment, CVD, cancer)	quetiapine	400, 600, 800, 800	6	74, 61, 68, 62	66	Weight, FG, Insulin, TC, LDL, HDL, TG
**Chan 2007**[46]	Taiwan Schizophrenia or schizoaffective disorder and were hospitalised with an acute relapse Aged 18-65	Pregnant, lactating, neurological disorder, any acute or unstable medical condition	Aripiprazole<br>risperidone	15<br>6	4	38<br>24	N/A	Weight, FG
**Chan 2010**[47]	Taiwan Schizophrenia neuroleptic-induced acute dystonia or parkinsonism research criteria Aged 18-65	Unstable major systemic diseases, neurological disorder that would influence the EPS assessment	Risperidone<br>olanzapine	3.45<br>11.72	8	28<br>29	N/A	Weight
**Chen 2011**[48]	Taiwan Schizophrenia with inadequate response to 1 FGA	None stated	Risperidone<br>olanzapine	4.3<br>17.8	8	13<br>13	N/A	Weight

**Czobor 2002[49]**	USA<br>Schizophrenia or schizoaffective disorder<br>Age not stated, likely 18+	None stated	Clozapine<br>Olanzapine<br>Risperidone<br>haloperidol	401.6<br>19.6<br>7.9<br>18.9	14	38<br>38<br>39<br>36	N/A	Weight, BMI
**Davidson 2007[50]**	North America, Canada, Eastern Europe, Asia, Israel, Mexico, South Africa<br>Schizophrenia<br>Aged 18+	'a medical condition that could affect absorption, metabolism or excretion of the study drug'	olanzapine, paliperidone	OLA: 10<br>PAL: 3, 15, 9	6	88, 70, 78, 82	47	Weight, BMI, FG, Insulin, TC, LDL, HDL, TG
**Del Valle 2006[51]**	Not specifically stated, likely USA<br>Schizophrenia or schizoaffective disorder<br>Aged 18+	Abnormal laboratory findings, abnormal ECG, evidence of T1DM	Ziprasidone<br><br>olanzapine	129.9<br><br>11.3	6	TG (76)<br>HDL (74)<br>LDL (70)<br>TC (76)<br>TG (78)<br>HDL (76)<br>LDL (64)<br>TC (78)	N/A	TG, HDL, LDL, TC
**Downing 2014[52]**	Not specifically stated<br>Schizophrenia<br>Aged 18-65	Not specified	risperidone	4	6	96	172	Weight, BMI
**Durgam 2014[53]**	United States, India, Russia, Ukraine, Malaysia<br>Schizophrenia<br>Aged 18-60	'Typical…medical/physical exclusions were applied'	cariprazine, risperidone	CAR: 4.5, 1.5, 3<br>RIS: 4	6	147, 145, 146, 140	151	Weight, FG, TC, LDL, HDL, TG
**Durgam 2015[54]**	United States, Romania, Russia, Ukraine<br>Schizophrenia<br>Aged 18-60	'BMI <18 or >40'	aripiprazole, cariprazine	ARI: 10<br>CAR: 6, 3	6	152, 157, 155	153	Weight, FG, TC, LDL, HDL, TG
**Durgam 2016[55]**	United States<br>Schizophrenia<br>Aged 18-65	Uncontrolled medical illness<br>BMI<18 or >40	cariprazine	3.83, 8.7	6	127, 133	129	Weight, FG, TC, LDL, HDL, TG
**Earley 2017[56] (Durgam 2014[53]; 2015[54]; 2016[55] and Kane 2015[57])**	United States, India, Russia, Ukraine, Malaysia<br>Schizophrenia<br>Aged 18-65	Uncontrolled medical illness<br>BMI<18 or >40	cariprazine<br>risperidone<br>aripiprazole	CAR: 2.25, 5.25 10.5<br>RIS: 4<br>ARI: 10	6	539, 575, 203, 140, 152	584	Weight, FG, TC, LDL, HDL, TG
**Fu 2014[58]**	Not specifically stated, likely USA<br>Schizophrenia<br>Aged not stated but likely 18+	None stated	Paliperidone<br>Risperidone	11.9<br>4.7	13	104<br>99	N/A	Weight
**Gattaz 2004[59]**	Germany<br>Schizophrenia<br>Aged 18+	None stated	Olanzapine<br>flupenthixol	15<br>10	4	15<br>13	N/A	Weight
**Gopal 2010[60]**	United States, Malaysia, the Republic of Korea,	Required BMI >17<br>'In general good health'	Paliperidone	3.43<br>6.86<br>10.29	13	47<br>53<br>12	51	Weight

	Taiwan, and Ukraine<br>Age 18+							
**Grootens 2011[61]**<br>**FEP**	Netherlands and Belgium<br>Schizophrenia schizoaffective disorder, or schizophreniform disorder<br>Aged 18-40	Epilepsy, organic mental disease, history of psychosurgery, significant medical illness, abnormal laboratory values, ECG abnormalities, use of medication prolonging QTc, pregnant, breast-feeding.	Olanzapine<br>ziprasidone	14<br>104	8	30<br>31	N/A	Weight, FG, TG, TC
**Hardy 2011[62]**	USA<br>Schizophrenia or schizoaffective disorder<br>Aged 18-65	BMI >40, DM, fasting hypertriglyceridaemia, use of medications known to affect insulin	Olanzapine<br>risperidone	12.9<br>4.3	12	50<br>46	N/A	Weight, FG, Insulin, TG, HDL, LDL, TC
**Hatta 2009[63]**	Japan<br>Schizophrenia, acute schizophrenia-like psychotic disorder, or schizoaffective disorder<br>Aged 18-64	Liver or renal dysfunction, heart or respiratory failure, DM, pregnant	Risperidone<br>Olanzapine<br>Quetiapine<br>Aripiprazole	7.2<br>17.4<br>579<br>23.6	8	20<br>17<br>20<br>21	N/A	Weight, FG, TG, TC
**Higuchi 2019[64]**	Japan, South Korea, Malaysia, Taiwan	Not specified	Lurasidone	40, 80	6	106, 116	110	Weight, BMI, FG, TC, LDL, TG
**Hwang 2001[65]**	Taiwan<br>Schizophrenia<br>Aged 16-65	Hypersensitivity to study medications, neurological diseases, abnormal EEG, severe medical disease, pregnant, lactating	Zotepine<br>Haloperidol	150<br>9	6	25<br>28	N/A	Weight
**Hwang 2003[66]**	Taiwan<br>Schizophrenia<br>Aged 18-65	Hypersensitivity to study medications, neurological diseases, severe medical diseases	Amisulpride<br>Risperidone	630<br>6.88	6	22<br>25	N/A	Weight
**Ishigooka 2018[67]**	Japan<br>Schizophrenia<br>Aged 18-65	Patients with concurrent diabetes or	brexpiprazole	1, 2, 4	6	72, 81, 68	70	FG, TC, LDL, HDL, TG

		history of diabetes were also excluded.						
**Jeste 2003**[68]	USA, Israel, Poland, Norway, Netherlands, Austria Schizophrenia or schizoaffective disorder Aged 60+	Major neurological disorder, any acute, unstable, significant, or untreated medical illness, any disease that could impair absorption, excretion, or metabolism of trial medication, history of NMS, QTc >500	risperidone olanzapine	2 10	8	63 71	N/A	Weight, BMI
**Kahn 2007**[69]	Bulgaria, Greece, India, Indonesia, the Philippines, Russia, Romania, South Africa Schizophrenia Aged 18-65	Not specified	quetiapine	400, 400, 600, 800	6	96, 83, 92, 90	85	Weight, FG, Insulin, TC, LDL, HDL, TG
**Kane 2007**[70]	Europe and India Schizophrenia Aged 18+	Not specified	olanzapine, paliperidone	OLA: 10 PAL: 9, 6, 12	6	90, 86, 80, 101	58	Weight, BMI, FG, Insulin, TC, LDL, HDL
**Kane 2008**[71]	Worldwide (not specifically stated) Schizophrenia or schizoaffective disorder Aged 18-65	Recent significant physical illness, a clinically significant abnormal laboratory finding, axis 1 disorder	iloperidone haloperidol	11.8 13.2	6	1231 403	N/A	Weight, FG, TG, TC
**Kane 2010**[72]	United States, Russia, Romania, Canada Schizophrenia Aged 18+	'A clinically significant medical condition or abnormal laboratory or physical examination findings'	asenapine, haloperidol	ASN: 10, 20 HAL: 8	6	68, 70, 65	69	Weight
**Kane 2011**[73]	USA and Canada Schizophrenia and had failed 1 adequate antipsychotic treatment in the previous 6 months Aged 18-55	QTc >500, significant somatic of medical disorder requiring frequent changes in medication, previous psychosurgery, Neurological abnormalities, clinically	sertindole risperidone	18.1 9	12	116 60	n/a	Weight, FG, TG, TC

		significant laboratory finding						
**Kane 2014[74] NCT01663532**	United States, Croatia, Latvia Schizophrenia Aged 18-65	Not specified	aripiprazole	14.3	12	108	85	Weight, FG, TC, LDL, HDL, TG
**Kelly 2008[75]**	Not specified Adults	Not specified	Olanzapine Risperidone	5-20 2-6	8	135 146	N/A	BMI, TC, LDL, TG
**Kinon 2011[76]**	Site not specified Schizophrenia Aged 18-65	Not specified	olanzapine	15	4	62	122	Weight
**Kinoshita 2016[77]**	Japan, Korea, Taiwan Schizophrenia Aged 20-65	BMI <16.0 or >35.0	asenapine	10, 20	6	114, 109	80	Weight, FG, FI,
**Kramer 2010[78]**	USA, Russia, Bulgaria, Poland, Ukraine, and India Schizophrenia Aged 18-65	medical conditions that could potentially alter the absorption, metabolism, or excretion of the study medication; relevant history of significant or unstable medical illness (other than schizophrenia)	paliperidone	7.43, 3.71	9.14	51, 47	27	Weight, BMI
**Krakowski 2009[79]**	USA Schizophrenia and schizoaffective disorder Age not stated, likely 18+	Substantial medical illness	Clozapine Olanzapine haloperidol	200-800 10-30 10-30	12	34 31 28	N/A	Weight, FG, TG, TC
**Landbloom 2017[80] NCT01617187**	United States, Bulgaria, Romania, Russia, Croatia Schizophrenia Aged 18+	Patients with uncontrolled, unstable, clinically significant medical conditions… body mass index <18 or >40.0 kg/m^2	asenapine, olanzapine	ASN: 5, 10 OLA: 15	6	52, 67, 35	60	FG, insulin, TC, TG
**Lauriello 2008[81]**	United States, Russia, Croatia Schizophrenia Aged 18-75	Unstable medical conditions	olanzapine	15, 21.43, 14.4	8	72, 67, 72	56	Weight, FG, TC, LDL, HDL, TG
**Lasser 2004[82]**	Not specifically stated, likely USA Schizophrenia or schizoaffective disorder Aged 18+	None stated	Olanzapine (smokers) Risperidone (smokers) Olanzapine (non-smoker) Risperidone (non-smoker)	Not stated	8	109 102 90 74	N/A	Weight, BMI

**Lawson 2009**[83]	USA Schizophrenia or schizoaffective disorder Aged 18+	None stated	ziprasidone	105	6	637	260	Weight, TG, TC
**Li 2014**[84]	China Schizophrenia Aged 18-65	Pregnant, lactating, neurological disorder, severe head trauma, unstable medical condition, clinically significant abnormal vital signs or laboratory values	Risperidone<br>aripiprazole	4.1<br>23	6	139<br>115	N/A	Weight
**Lin 2013**[85]	Taiwan Schizophrenia, on clozapine for at least 6 months Aged 20-65	Significant medical illness, ECG or clinically significant lab abnormalities	Zotepine<br>clozapine	397.1<br>377.1	12	28<br>24	N/A	Weight, TG, FG
**Lieberman 2003**[86]	North America and Western Europe schizophrenia, schizophreniform disorder or schizoaffective disorder Aged 16-40	Pregnant, breast feeding, serious unstable medical illness	olanzapine<br>haloperidol	12.5<br>11	12	89<br>71	N/A	Weight, BMI, TC, FG
**Lieberman 2016**[87]	United States Schizophrenia Aged 18-55	Diagnosis of various medical conditions	risperidone	4	4	63	61	Weight, FG, insulin, TC, TG
**Lindenmayer 2003**[88]	USA Schizophrenia and schizoaffective disorder Age not stated, likely 18+	Substantial medical illness	Clozapine<br>haloperidol<br>olanzapine<br>risperidone	443.7<br>20<br>20.1<br>8.5	8	28<br>25<br>26<br>22	N/A	FG, TC
**Lindenmayer 2008**[89] **5077IL/0041**	United States and Canada Schizophrenia Aged 18-65	'any clinically relevant other diseases (such as renal or hepatic impairment, significant coronary artery disease)'	quetiapine	QUET: 300, 600, 800, 300, 600	6	35, 40, 41, 41, 34	29	Weight, FG, TC, LDL, HDL, TG
**Litman 2014**[90]	United States Schizophrenia Aged 18-65	Not specified	olanzapine	15	4	18	33	Weight

**Litman 2016**[91]	United States Schizophrenia Aged 18-65	Participants were 'medically stable'	risperidone	4	4	26	40	Weight
**Loebel 2013**[92]	United States, Russia, India, Ukraine, Romania, Colombia Schizophrenia Aged 18-75	Not specified	lurasidone, quetiapine	LUR: 160, 80 QUET: 600	6	93, 89, 97	74	Weight, BMI, FG, Insulin, TC, LDL, HDL, TG
**Loebel 2016**[93]	United States Schizophrenia Aged 18-75	Not specified	lurasidone	20, 80, 160	6	74, 45, 36	70	Weight, FG, TC, LDL, TG Symptoms
**Marder 2007**[94]	United States Schizophrenia Aged 18+	medical conditions affecting absorption, metabolism, or excre-tion of the study drug	paliperidone olanzapine	PAL: 6, 12 OLA: 10	6	51, 54, 50	37	Weight, FG, Insulin, TC, LDL, HDL, TG
**Martin 2002**[95]	Belgium, Switzerland, Denmark, France, Great Britain, Czech Republic, Tunisia, Hungary, Morocco, and Portugal Schizophrenia or schizophreno form disorder Aged 18-65	Pregnant or lactating	olanzapine amisulpride	12 489	8	146 150	N/A	Weight, BMI
**Meltzer 2008**[96] NCT00077714 NCT00083668 NCT00078039	'23 different countries on 5 continents' Schizophrenia Aged 18+	Not specified	paliperidone olanzapine	PAL: 3,6,9,12,15 OLA: 10	6	70, 131, 164, 155, 82, 228	142	Weight
**Meltzer 2011**[97]	United States, Colombia, Lithuania, Colombia, Asia, the Philippines Schizophrenia Aged 18-65	Not specified	lurasidone, olanzapine	LUR: 40, 120 OLA: 15	6	77, 66, 84	71	Weight, BMI, FG, Insulin, HOMA-IR, TC, LDL, HDL, TG
**Meulien 2010**[98]	Eastern Europe, Asia, India, South Africa, Canada Schizophrenia Aged 18-65	'evidence of other clinically relevant conditions (e.g. renal or hepatic impairment, significant coronary artery disease)'	quetiapine	QUE: 591.48, 530.92	6	555, 215	225	Weight, FG, Insulin, TC, LDL, HDL, TG
**Nasrallah 2010**[99]	Schizophrenia Age not specified (mean 41) United States, South Africa, Bulgaria,	BMI <15, abnormal screening bloods	paliperidone	1.71, 3.43, 6.86	13	70, 70, 75	48	Weight, BMI

	Romania, Russia, Bulgaria							
**Nasrallah 2013**[100]	United States, Russia, India, Ukraine, Romania, Malaysia, and France Schizophrenia Aged 18-75	acute or unstable medical condition	lurasidone	80, 120, 40	6	86, 85, 84	73	Weight, BMI
**Nasrallah 2016**[101]	‘8 countries’ Schizophrenia Aged 18-70	(BMI) of 18.5 to 40 kg/m^2	aripiprazole	15,75, 31.5	12	207,208	207	Weight, FG, TC, LDL, HDL, TG
**Nasser 2016**[102]	Sites not specified Schizophrenia Aged 18-55	Not specified	risperidone	4.2, 2.1	8.14	85, 90	84	Weight
**Newcomer 2008**[103,104]	International study with countries not specified, likely USA Schizophrenia or schizoaffective disorder must have received olanzapine monotherapy at a dose of 10 to 20 mg/day for 1 to 24 months immediately prior to screening Aged 18-65	Pregnant, breast feeding, BMI <27, DM, significant neurological disease, clinically significant vital sign, electrocardiogram (ECG), or lab- oratory test abnormalities	olanzapine aripiprazole	15.9 16	16	63 56	n/a	Weight, FG, Insulin, TG, HDL, LDL, TC
**Ogasa 2013**[105]	United States Schizophrenia Aged 18-64	excluded if they had an acute or unstable medical condition	lurasidone	120, 40	6	20, 16	15	Weight
**Patil 2007**[106]	Russia Schizophrenia Aged 18-65	Not specified	Olanzapine	15	4	27	26	Weight
**Patel 2009**[107]	USA and Canada schizophrenia, schizophreniform, or schizoaffective disorder Aged 16-40	Pregnant, breast feeding, history of mental retardation	olanzapine quetiapine risperidone	2.5-20 100-800 0.5-4	12	133 134 144	N/A	Weight, BMI, FG, HDL
**Potkin 2003**[108]	United States Schizophrenia and schizoaffective disorder Aged 18-65	‘Any acute or unstable medical condition’	aripiprazole risperidone	ARI: 20, 30 RIS: 6	4	61, 67, 62	52	Weight
**Porkin 2006**[109]	United States, India, Romania Schizophrenia Aged 18-64	Evidence of a clinically significant or unstable disease	risperidone quetiapine	RIS: 5 QUET: 700	2	139, 132	60	Weight

**Potkin 2011**[110]	USA Schizophrenia or schizoaffective disorder Aged 18-70	Pregnant, breast feeding, clinically significant medical illness, abnormal laboratory or ECG parameters	lurasidone<br>ziprasidone	120<br>160	3	104<br>106	N/A	Weight, TG, TC
**Potkin 2015**[111]	United States Schizophrenia Aged 18-64	Clinically significant cardiovascular disease	lurasidone<br>haloperidol	LUR: 20, 40, 80<br>HAL: 10	6	27, 28, 31, 29	36	Weight
**Riedel 2005**[112]	Not stated, likely Germany Schizophrenia Age not stated, likely 18+	Pregnant, lactating, significant medical history, laboratory, ECG, or EEG abnormalities	quetiapine<br>risperidone	589.7<br>4.9	12	13<br>12	N/A	Weight
**Riedel 2007**[113]	Not stated, likely Germany Schizophrenia Aged 18-65	Significant medical history, ECG or EEG abnormalities, abnormal laboratory tests, pregnant, lactating	quetiapine<br>olanzapine	586.86<br>15.82	8	16<br>17	N/A	Weight
**Robinson 2015**[114]	USA and Canada schizophrenia, schizophreniform, schizoaffective disorder, or psychotic disorder Not Otherwise Specified Aged 15-40	Serious neurological or endocrine disorder or medical condition/treatment known to affect the brain; medical conditions requiring treatment with a medication with psychotropic effects, DM, metabolic syndrome	aripiprazole<br>risperidone	14.8<br>3.2	12	102<br>96	N/A	Weight
**Saddichha 2007**[115]	India Schizophrenia Likely 18+	History of severe psychical illness, history of hypertension or DM or family history of either	olanzapine<br>risperidone<br>haloperidol		6	29<br>22<br>15	N/A	Weight, BMI
**Schmidt 2012**[116]	Bulgaria, Estonia, Korea, Lithuania, Malaysia, Romania,	BMI<40, significant or unstable disease, NMS,	olanzapine	15	6	71	49	Weight, BMI

	Russia, South Africa, Taiwan, and Ukraine Schizophrenia Aged 18-65							
**Shen 2014**[117]	United States, India Schizophrenia Aged 20-63	Not specified	olanzapine	15	6	71	71	Weight, FG, TC, LDL, HDL, TG
**Sliwa 2011**[118]	Study sites not specified Schizophrenia Age 18+	Not specified	Paliperidone	3.43, 6, 7.71	13	24, 31, 29	22	Weight
**Tollefson 1997**[119]	USA and Europe schizophrenia, schizophreniform disorder, or schizoaffective disorder Aged 18+	Not stated	olanzapine<br>haloperidol	13.2<br>11.8	6	888<br>309	N/A	Weight
**Tzimos 2008**[120]	Study sites not specified Schizophrenia Age 65+	Not specified	paliperidone	7.5	6	64	26	Weight
**Vanelle 2006**[121]	France, Italy, Tunisia Schizophrenia and major or minor depressive episode Aged 18-65	Pregnant, lactating, severe somatic disease, Parkinson's disease, a history of seizures, prostatic hypertrophy, paralytic ileus, phaeochromocytoma, < 55 bpm, known hyperkalaemia, congenital prolongation of the QT interval and known risk for narrow-angle glaucoma.	olanzapine<br>amisulpride	11.4<br>471	8	36<br>35	N/A	Weight, FG, TG, HDL, TC
**Wang 2006**[122]	USA Schizophrenia spectrum disorder and stable on conventional antipsychotic medication for at least 2 years Not stated, likely 18+	Unstable metabolic, hematologic, cardiovascular, hepatic or renal function were excluded from the study	risperidone<br>olanzapine	5.3<br>13.8	7	14<br>13	N/A	Weight
**Wetzel 1998**[123]	Germany Schizophrenia, paranoid type,	Axis I disorder, Parkinson's disease,	amisulpride<br>flupenthixol	956<br>22.6	6	51<br>37	N/A	Weight

	or undifferentiated type<br>Aged 18-65	epilepsy, narrow-angle glaucoma, prostate hypertrophy, urinary retention, severe hypotonia or arteriosclerosis, severe liver or kidney dysfunction, other serious somatic co-morbid disorders, significant laboratory, ECG or EEG abnormality, pregnancy or lactation					
**Zhong 2006[124]**	USA<br>Schizophrenia<br>Aged 18-6	Pregnant, lactating, psychotic disorder due to general medical condition, mental retardation	quetiapine<br>risperidone	8	154<br>168	N/A	BMI

eTable 3. Cochrane risk of bias assessment (low, high, unclear risk of bias)

Study	Random sequence generation	Allocation concealment	Blinding of participants and personnel	Blinding of outcome assessment	Incomplete outcome data addressed	Selective reporting
Ascher-Svanum 2005[26] Beasley 1996[31]	Low risk	Unclear risk	Low risk	Low risk	Low risk	High Risk
Azorin 2006[28]	Unclear risk	Unclear risk	Low risk	Low risk	Unclear risk	Low risk
Baymiller 2002[29]	High risk	Unclear risk	Low risk	Low risk	Unclear risk	Low risk
Bhowmick 2010[30]	Low risk	Low risk	Low risk	Low risk	Low risk	Unclear risk
Beasley 1996[31]	Unclear risk	Unclear risk	Low risk	Low risk	Unclear risk	Unclear risk
**Bossie, 2011[32]**	**Low risk**	**Low risk**	**Low risk**	**Low risk**	**Low risk**	**Low risk**
Breier 2005[33]	Unclear risk	Unclear risk	Low risk	Low risk	Low risk	Low risk
Buckley 2004[34]	Unclear risk	Unclear risk	Low risk	Low risk	Unclear risk	Unclear risk
Cantillon 2017[35]	Low risk	Unclear risk	Low risk	Low risk	Low risk	Low risk
**Canuso 2009[36]**	**Low risk**	**Low risk**	**Low risk**	**Low risk**	**Low risk**	**Low risk**
Canuso 2010a[37]	Low risk	Unclear risk	Low risk	Low risk	Low risk	Low risk
**Canuso 2010b[38]**	**Low risk**	**Low risk**	**Low risk**	**Low risk**	**Low risk**	**Low risk**
Canuso 2010[39]	Low risk	Unclear risk	Low risk	Low risk	Low risk	Low risk
Conley 2001[40]	Unclear risk	Unclear risk	Low risk	Low risk	Low risk	Low risk
Conley 2005[41]	Unclear risk	Unclear risk	Unclear risk	Low risk	Unclear risk	Unclear risk
Coppola 2011[42]	Unclear risk	Low risk	Low risk	Low risk	Low risk	Low risk
Correll 2016[43] (Correll 2015[44] NCT00905307 NCT01396421 NCT01393613)	Low risk	Unclear risk	Low risk	Low risk	Low risk	High risk
Cutler 2011[45]	Low risk	Unclear risk	Low risk	Low risk	Low risk	Low risk
Chan 2007[46]	Unclear risk	Unclear risk	Unclear risk	Low risk	Unclear risk	Unclear risk
Chan 2010[47]	Unclear risk	Unclear risk	Low risk	Low risk	Low risk	Unclear risk
Chen 2011[48]	Unclear risk	Unclear risk	Low risk	Low risk	Unclear risk	Unclear risk
Czobor 2002[49]	Unclear risk	Unclear risk	Low risk	Low risk	Unclear risk	Unclear risk
Lawson 2009[83] Containing: Daniel 1999[125] Keck 1998[126]	Low risk	Unclear risk	Low risk	Low risk	Low risk	Unclear risk
Davidson 2007[50]	Unclear risk	Low risk	Low risk	Low risk	Low risk	Low risk
Del Valle 2006[51]	Unclear risk	Unclear risk	Unclear risk	Low risk	Unclear risk	Unclear risk
Downing 2014[52]	Low risk	Unclear risk	Low risk	Low risk	Low risk	High risk
Durgam 2014[53]*	Low risk	Unclear risk	Low risk	Low risk	Low risk	Low risk
Durgam 2015[54]*	Low risk	Unclear risk	Low risk	Low risk	Low risk	Low risk
Durgam 2016[55]*	Low risk	Unclear risk	Low risk	Low risk	Low risk	Low risk
Earley 2017[56]	Low risk	Unclear risk	Low risk	Low risk	Low risk	Low risk
Fu 2014[58]	Unclear risk	Unclear risk	Low risk	Low risk	Unclear risk	Unclear risk
Gattaz 2004[59]	Unclear risk	Unclear risk	Unclear risk	Low risk	Unclear risk	Unclear risk
Gopal 2010[60]	Unclear risk	Unclear risk	Unclear risk	Low risk	Unclear risk	Unclear risk
Grootens 2011[61]	Unclear risk	Unclear risk	Low risk	Low risk	Low risk	Low risk
Hardy 2011[62]	Unclear risk	Unclear risk	Low risk	Low risk	Low risk	Low risk
Hatta 2009[63]	Unclear risk	Unclear risk	Low risk	Low risk	Low risk	Low risk
**Higuchi 2019[64]**	**Low risk**	**Low risk**	**Low risk**	**Low risk**	**Low risk**	**Low risk**
Hwang 2001[65]	Unclear risk	Low risk	Low risk	Low risk	Low risk	Low risk
Hwang 2003[66]	Unclear risk	Unclear risk	Unclear risk	Low risk	Unclear risk	Unclear risk
**Ishigooka 2018[67]**	**Low risk**	**Low risk**	**Low risk**	**Low risk**	**Low risk**	**Low risk**
Jeste 2003[68]	Unclear risk	Unclear risk	Unclear risk	Low risk	Unclear risk	Unclear risk
Kahn 2007[69]	Low risk	Unclear risk	Low risk	Low risk	Low risk	Low risk
Kane 2007[70]	Unclear	Low risk	Low risk	Low risk	Low risk	Low risk
Kane 2008[71]	Unclear risk	Unclear risk	Low risk	Low risk	Low risk	Low risk
Kane 2010[72]	Low risk	Unclear risk	Low risk	Low risk	Low risk	High risk
Kane 2011[73]	Unclear risk	Unclear risk	Unclear risk	Low risk	Unclear risk	Unclear risk
**Kane 2014[74]**	**Low risk**	**Low risk**	**Low risk**	**Low risk**	**Low risk**	**Low risk**
Kelly 2008[75]	Unclear risk	Unclear risk	Unclear risk	Low risk	Unclear risk	Unclear risk
Kane 2015[57]	Low risk	Unclear risk	Low risk	Low risk	Low risk	Low risk
Kinon 2011[76]	Low risk	Unclear risk	Low risk	Low risk	Low risk	Low risk
**Kinoshita 2016[77]**	**Low risk**	**Low risk**	**Low risk**	**Low risk**	**Low risk**	**Low risk**
Kramer 2010[78]	Low risk	Low risk	Low risk	Low risk	Low risk	High risk
Krakowski 2009[79]	Unclear risk	Unclear risk	Low risk	Low risk	Low risk	Low risk
Landbloom 2017[80]	Low risk	Unclear risk	Low risk	Low risk	Low risk	Low risk
Lauriello 2008[81]	Low risk	Unclear risk	Low risk	Low risk	Low risk	Low risk

Lasser 2004[82]	Unclear risk	Unclear risk	Unclear risk	Low risk	Low risk	Low risk
Lawson 2009[83]	Unclear risk	Unclear risk	Unclear risk	Low risk	Unclear risk	High risk
Li 2014[84]	Unclear risk	Low risk	Low risk	Low risk	Unclear risk	High risk
Lin 2003[85]	Unclear risk	Unclear risk	Unclear risk	Low risk	Low risk	Low risk
Lieberman 2003[86]	Unclear risk	Unclear risk	Low risk	Low risk	Low risk	Low risk
**Lieberman 2016[87]**	**Low risk**	**Low risk**	**Low risk**	**Low risk**	**Low risk**	**Low risk**
Lindenmayer 2003[88]	Unclear risk	Unclear risk	Low risk	Unclear risk	Low risk	Low risk
Lindenmayer 2008[89]	Low risk	Unclear risk	Low risk	Low risk	Low risk	Low risk
**Litman 2014[90]**	**Low risk**	**Low risk**	**Low risk**	**Low risk**	**Low risk**	**Low risk**
**Litman 2016[91]**	**Low risk**	**Low risk**	**Low risk**	**Low risk**	**Low risk**	**Low risk**
**Loebel 2013[92]**	**Low risk**	**Low risk**	**Low risk**	**Low risk**	**Low risk**	**Low risk**
**Loebel 2016[93]**	**Low risk**	**Low risk**	**Low risk**	**Low risk**	**Low risk**	**Low risk**
**Marder 2007[94]**	**Low risk**	**Low risk**	**Low risk**	**Low risk**	**Low risk**	**Low risk**
Martin 2002[95]	Unclear risk	Unclear risk	Low risk	Low risk	Unclear risk	Unclear risk
Meltzer 2008[96]	Low risk	Unclear risk	Low risk	Low risk	Low risk	Low risk
**Meltzer 2011[97]**	**Low risk**	**Low risk**	**Low risk**	**Low risk**	**Low risk**	**Low risk**
Meulien 2010[98]	Unclear risk	Low risk	Low risk	Low risk	Low risk	Low risk
Nasrallah 2010[99]	Low risk	Unclear risk	Low risk	Low risk	Low risk	Low risk
Nasrallah 2013[100]	Low risk	Unclear risk	Low risk	Low risk	Low risk	High risk
Nasrallah 2016[101]	Low risk	Unclear risk	Low risk	Low risk	Low risk	High risk
Nasser 2016[102]	Low risk	Low risk	Low risk	Low risk	Low risk	High risk
Newcomer 2008[103,104]	Unclear risk	Unclear risk	Low risk	Low risk	Low risk	Low risk
Ogasa 2013[105]	Low risk	Unclear risk	Low risk	Low risk	Low risk	High risk
Patil 2007[106]	Unclear risk	Low risk	Low risk	Low risk	Low risk	Low risk
Patel 2009[107]	Unclear risk	Unclear risk	Unclear risk	Low risk	Low risk	Low risk
Potkin 2003[108]	Low risk	Unclear risk	Low risk	Low risk	Low risk	High risk
Porkin 2006[109]	Low risk	Unclear risk	Low risk	Low risk	Low risk	High risk
**Potkin 2011[110]**	**Low risk**	**Low risk**	**Low risk**	**Low risk**	**Low risk**	**Low risk**
Potkin 2015[111]	Low risk	Low risk	Low risk	Low risk	Low risk	High risk
Riedel 2005[112]	Unclear risk	Unclear risk	Unclear risk	Low risk	Unclear risk	Unclear risk
Riedel 2007[113]	Unclear risk	Unclear risk	Unclear risk	Low risk	Unclear risk	Unclear risk
Robinson 2015[114]	Unclear risk	Unclear risk	Low risk	Low risk	Low risk	Low risk
Saddichha 2007[115]	Unclear risk	Unclear risk	Unclear risk	Low risk	Unclear risk	Unclear risk
**Schmidt 2012[116]**	**Low risk**	**Low risk**	**Low risk**	**Low risk**	**Low risk**	**Low risk**
**Shen 2014[117]**	**Low risk**	**Low risk**	**Low risk**	**Low risk**	**Low risk**	**Low risk**
Sliwa 2011[118]<br>Pandina 2010[127]	Unclear	Low risk	Low risk	Low risk	Low risk	Low risk
Tollefson 1997[119]	Unclear risk	Unclear risk	Unclear risk	Low risk	Unclear risk	Unclear risk
Tzimos 2008[120]	Low risk	Unclear risk	Low risk	Low risk	Low risk	High risk
**Vanelle 2006[121]**	**Low risk**	**Low risk**	**Low risk**	**Low risk**	**Low risk**	**Low risk**
Wang 2006[122]	Unclear risk	Unclear risk	Unclear risk	Low risk	Unclear risk	Unclear risk
Wetzel 1998[123]	Unclear risk	Unclear risk	Low risk	Low risk	Unclear risk	Unclear risk
Zhong 2006[124]	Unclear risk	Unclear risk	Unclear risk	Low risk	Unclear risk	Unclear risk

eTable 4. Baseline parameters across treatment-comparisons

Treatment Comparison	Mean Age (number of studies)	Mean % male (number of studies)	Mean % non-Caucasian (number of studies)	Mean Baseline body weight (number of studies)
amisulpride:flupenthixol	34.00 (1)	56.00 (1)	.	71.10 (1)
amisulpride:olanzapine	34.60 (3)	59.12 (2)	.	73.00 (2)
amisulpride:risp_pali	35.20 (2)	50.00 (1)	.	64.50 (1)
aripiprazole:brexpiprazole	39.42 (1)	63.96 (1)	35.40 (1)	.
aripiprazole:cariprazine	38 .02 (1)	70.23 (1)	55.14 (1)	76.64 (1)
aripiprazole:olanzapine	39.27 (3)	52.77 (3)	30.00 (2)	81.47 (2)
aripiprazole:placebo	38.91 (4)	72.77 (4)	57.51 (4)	81.47 (2)
aripiprazole:risp_pali	34.56 (6)	60.46 (6)	51.79 (4)	71.73 (4)
asenapine:placebo	41.41 (1)	48.07 (1)	.	63.25 (1)
brexpiprazole:placebo	41.85 (2)	55.73 (2)	35.40 (1)	.
cariprazine:placebo	38.03 (1)	70.23 (1)	55.14 (1)	76.64 (1)
cariprazine:risp_pali	38.03 (1)	70.23 (1)	55.14 (1)	76.64 (1)
clozapine:haloperidol	36.22 (3)	78.02 (3)	54.23 (2)	84.75 (2)
clozapine:olanzapine	37.46 (2)	83.78 (2)	82.06 (1)	87.60 (1)
clozapine:risp_pali	40.95 (1)	84.75 (1)	.	.
flupenthixol:olanzapine	32.40 (1)	48.00 (2)	.	.
fluphenazine:quetiapine	44.73 (1)	79.00 (1)	52.67 (1)	88.53 (1)
fluphenazine:risp_pali	44.73 (1)	79.00 (1)	52.67 (1)	88.53 (1)
haloperidol:lurasidone	41.23 (1)	73.83 (1)	52.25 (1)	89.04 (1)
haloperidol:olanzapine	32.89 (3)	83.02 (3)	64.54 (2)	81.60 (2)
haloperidol:placebo	38.67 (1)	72.67 (1)	38.33 (1)	77.25 (1)
haloperidol:quetiapine	39.80 (1)	64.45 (1)	.	.
haloperidol:risp_pali	40.96 (1)	84.75 (1)	.	.
haloperidol:ziprasidone	38.67 (1)	72.67 (1)	38.33 (1)	77.25 (1)
haloperidol:zotepine	34.3 (1)	54.00 (1)	.	61.5 (1)
lurasidone:olanzapine	37.63 (1)	78.00 (1)	64.00 (1)	.
lurasidone:placebo	39.56 (6)	69.01 (6)	57.46 (6)	72.44 (3)
lurasidone:ziprasidone	42.90 (1)	70.45 (1)	75.5 (1)	.
olanzapine:placebo	38.47 (7)	74.98 (7)	41.76 (7)	80.39 (5)
olanzapine:quetiapine	33.61 (3)	60.83 (3)	27.33 (2)	.
olanzapine:risp_pali	38.94 (10)	60.97 (10)	57.37 (8)	81.13 (3)
olanzapine:ziprasidone	34.98 (3)	64.25 (1)	56.35 (1)	80.91 (2)
placebo:quetiapine	39.69 (4)	65.64 (4)	53.60 (4)	78.59 (2)
placebo:risp_pali	38.04 (16)	65.37 (16)	50.81 (13)	78.81 (13)
placebo:sertindole	36.23 (1)	.	.	.
placebo:ziprasidone	38.02 (2)	74.83 (2)	31.33 (1)	75.43 (1)
quetiapine:risp_pali	36.84 (6)	66.44 (6)	44.90 (5)	87.55 (2)
risp_pali:sertindole	36.47 (3)	67.22 (3)	29.58 (2)	73.49 (3)
ziprasidone:haloperidol	38.67 (1)	72.67 (1)	38.33 (1)	77.25 (1)

eAppendix 3. Pairwise meta-analyses

There were 3 pairwise comparisons for antipsychotic-induced weight gain with ≥10 studies allowing meta-analysis: olanzapine vs. placebo, risperidone/paliperidone vs. placebo, and olanzapine vs. risperidone/paliperidone (forest plots recording in eFigures 2-4). Olanzapine was associated increased weight gain compared with placebo (MD: +2.62kg 95%CI: 1.95-3.29, p<0.0001), risperidone/paliperidone with increased weight gain compared with placebo (MD: +1.29kg 95%CI: 0.99-1.59, p<0.0001), and olanzapine with increased weight gain compared with risperidone/paliperidone (MD: +1.29kg 95% CI: 0.99-1.59, p<0.0001).

eFigure 5. Pairwise meta-analysis forest plot: placebo vs olanzapine

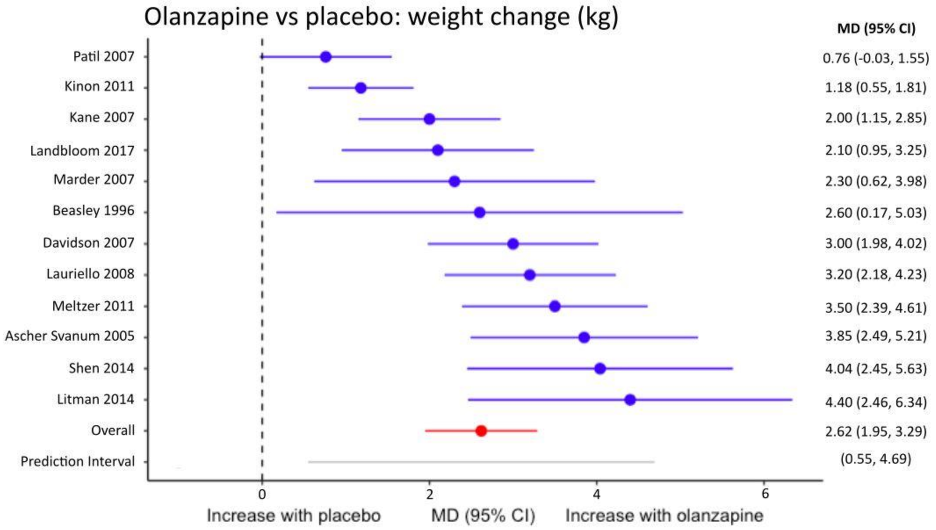

eFigure 6. Pairwise meta-analysis forest plot: placebo vs risperidone/paliperidone

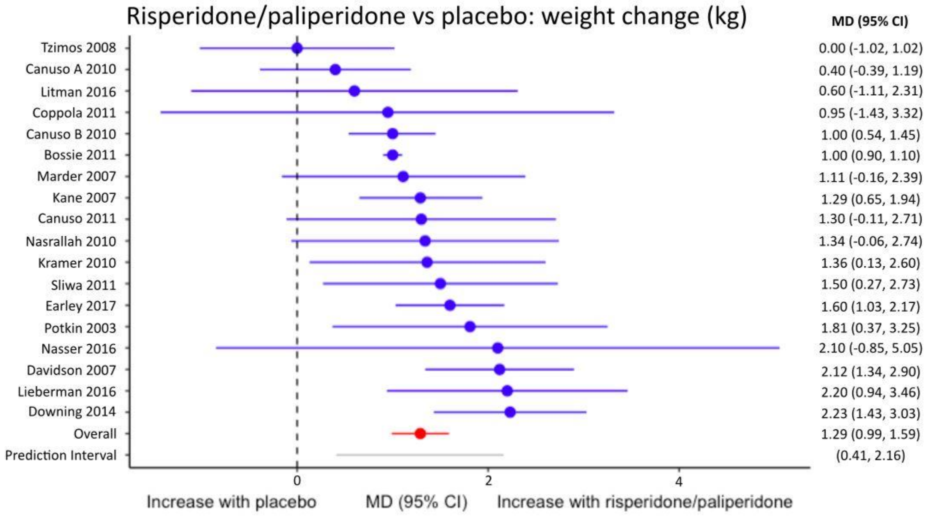

eFigure 7. Pairwise meta-analysis forest plot: risperidone/paliperidone versus olanzapine

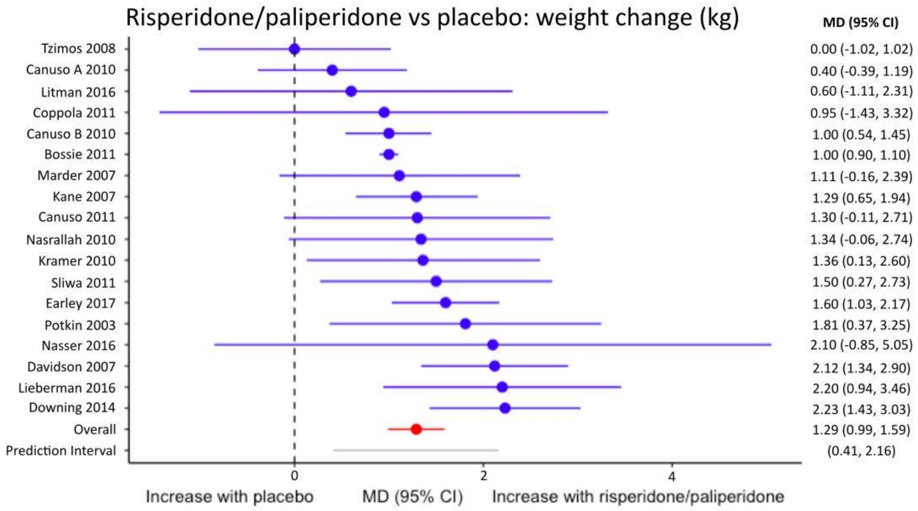

Heterogeneity was medium to large (I^2 values: 59.85-76.51%), with τ ranging from 0.42-1.00kg (see below). Visual inspection of funnel plots (eFigures 8-10) suggested symmetry for comparisons except for olanzapine vs. risperidone/paliperidone (where an absence in studies reporting increases in weight with risperidone/paliperidone compared with olanzapine was noted). By contrast, Egger's regression tests only demonstrated significance for the comparison of olanzapine vs placebo (p=0.01).

Pairwise meta-analysis: Assessment of heterogeneity

Tau

Placebo vs olanzapine: 1.00kg

Placebo vs risperidone/paliperidone: 0.42kg

Risperidone/paliperidone vs olanzapine: 0.54kg

I^2 statistic

Placebo vs olanzapine: 76.51%

Placebo vs risperidone/paliperidone: 59.85%

Risperidone/paliperidone vs olanzapine: 64.41%

Pairwise meta-analysis: Egger's Regression test

Placebo vs olanzapine: z = 2.50, p = 0.01

Placebo vs risperidone/paliperidone: z = 0.49, p = 0.63

Risperidone/paliperidone vs olanzapine: -1.55, p = 0.12

eFigure 8. Pairwise meta-analysis contour-enhanced funnel plot: placebo vs olanzapine

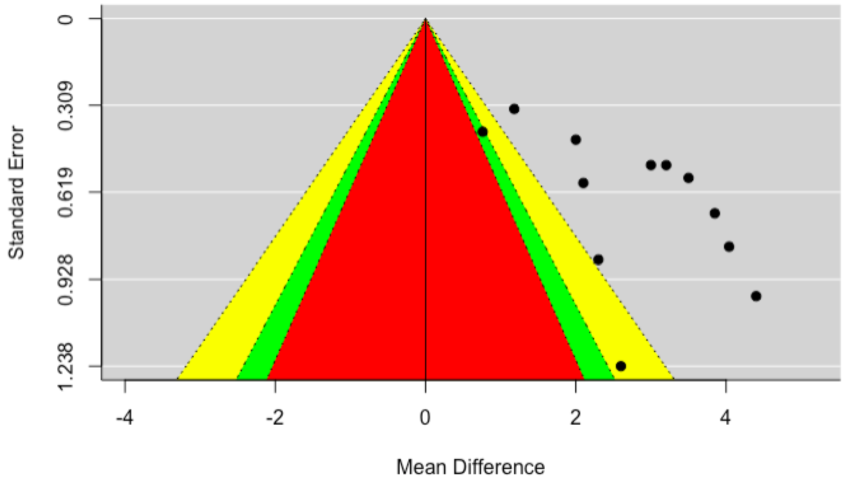

Key: Red: 0.10 < p ≤ 1.00

Green: 0.05 < p ≤ 0.10

Yellow: 0.01 < p ≤ 0.05

Grey: 0.00 < p ≤ 0.01

eFigure 9. Pairwise meta-analysis contour-enhanced funnel plot: placebo vs risperidone/paliperidone

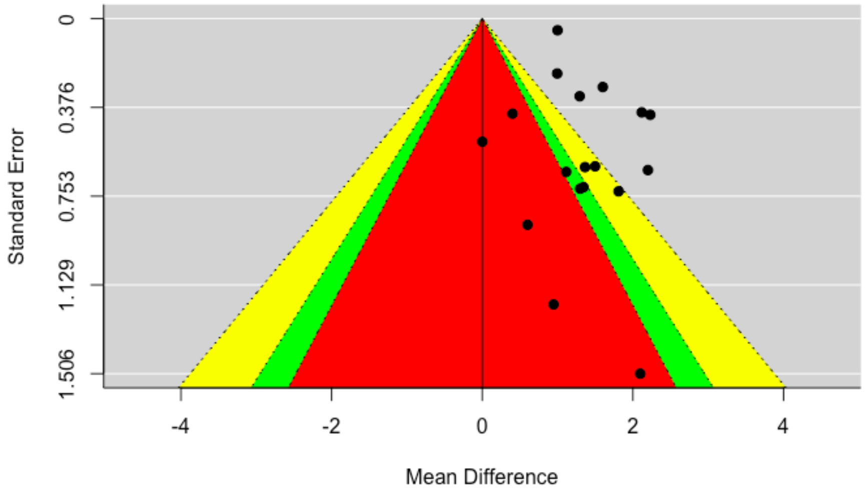

Key:

Red: 0.10 < p ≤ 1.00

Green: 0.05 < p ≤ 0.10

Yellow: 0.01 < p ≤ 0.05

Grey: 0.00 < p ≤ 0.01

eFigure 10. Pairwise meta-analysis contour-enhanced funnel plot: risperidone/paliperidone versus olanzapine

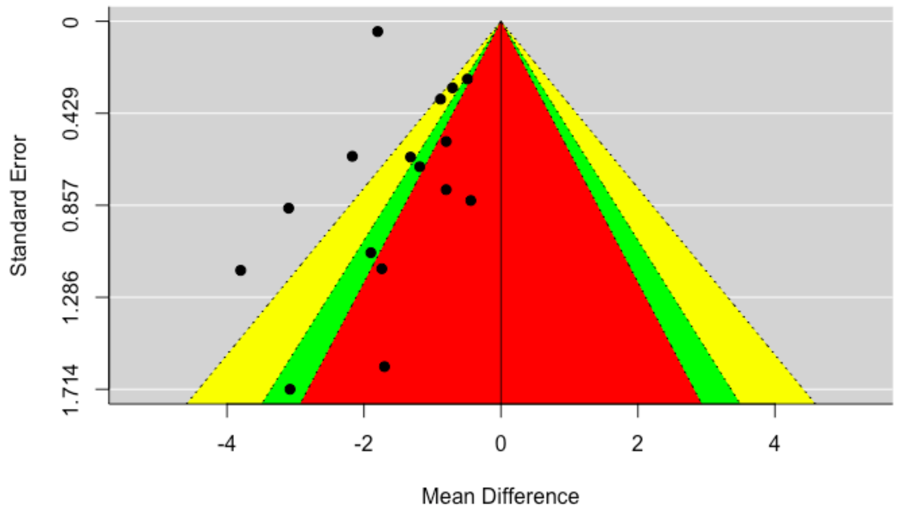

Key:

Red: 0.10 < p ≤ 1.00

Green: 0.05 < p ≤ 0.10

Yellow: 0.01 < p ≤ 0.05

Grey: 0.00 < p ≤ 0.01

eTable 5. League table for changes in body weight (kg) associated with antipsychotic drug treatment. For interpretation, a number larger than zero favours the column-defining treatment of a cell, i.e. this treatment leads to an increase in weight. Values depicted are mean differences with associated 95% confidence intervals.

**Amisulpride**																	
0.32 (-0.68 to 1.31)	**Aripiprazole**																
-0.50 (-1.61 to 0.61)	-0.82 (-1.67 to 0.03)	**Asenapine**															
-0.21 (-1.42 to 0.99)	-0.53 (-1.43 to 0.37)	0.29 (-0.78 to 1.36)	**Brexpiprazole**														
0.00 (-1.32 to 1.33)	-0.32 (-1.35 to 0.72)	0.51 (-0.71 to 1.72)	0.22 (-1.06 to 1.50)	**Cariprazine**													
-2.35 (-3.83 to -0.87)	-2.67 (-3.98 to -1.36)	-1.85 (-3.22 to -0.48)	-2.14 (-3.61 to -0.66)	-2.35 (-3.93 to -0.78)	**Clozapine**												
-0.09 (-1.59 to 1.41)	-0.41 (-2.03 to 1.22)	0.41 (-1.28 to 2.11)	0.12 (-1.64 to 1.89)	-0.09 (-1.94 to 1.75)	2.26 (0.31 to 4.22)	**Flupenthixol**											
0.50 (-6.73 to 7.73)	0.18 (-7.01 to 7.37)	1.00 (-6.21 to 8.21)	0.71 (-6.51 to 7.93)	0.50 (-6.75 to 7.74)	2.85 (-4.42 to 10.13)	0.59 (-6.75 to 7.93)	**Fluphenazine**										
0.90 (-0.12 to 1.91)	0.58 (-0.17 to 1.32)	1.40 (0.58 to 2.22)	1.11 (0.10 to 2.11)	0.89 (-0.26 to 2.04)	3.25 (2.10 to 4.39)	0.99 (-0.65 to 2.62)	0.40 (-6.80 to 7.59)	**Haloperidol**									
-1.10 (-2.69 to 0.49)	-1.42 (-2.86 to 0.01)	-0.60 (-2.08 to 0.88)	-0.89 (-2.48 to 0.69)	-1.11 (-2.79 to 0.57)	1.25 (-0.43 to 2.93)	-1.01 (-3.06 to 1.03)	-1.60 (-8.90 to 5.70)	-2.00 (-3.23 to -0.77)	**Iloperidone**								
0.18 (-0.82 to 1.18)	-0.14 (-0.83 to 0.55)	0.68 (-0.16 to 1.52)	0.39 (-0.56 to 1.34)	0.18 (-0.94 to 1.29)	2.53 (1.23 to 3.83)	0.27 (-1.36 to 1.90)	-0.32 (-7.51 to 6.87)	-0.72 (-1.44 to 0.01)	1.28 (-0.14 to 2.71)	**Lurasidone**							
-2.06 (-2.90 to -1.22)	-2.38 (-2.94 to -1.83)	-1.56 (-2.30 to -0.82)	-1.85 (-2.73 to -0.97)	-2.07 (-3.11 to -1.03)	0.29 (-0.93 to 1.51)	-1.97 (-3.51 to -0.44)	-2.56 (-9.74 to 4.62)	-2.96 (-3.54 to -2.38)	-0.96 (-2.32 to 0.40)	-2.24 (-2.81 to -1.67)	**Olanzapine**						
0.66 (-0.23 to 1.56)	0.34 (-0.16 to 0.84)	1.17 (0.47 to 1.86)	0.88 (0.06 to 1.69)	0.66 (-0.35 to 1.66)	3.01 (1.78 to 4.24)	0.75 (-0.81 to 2.32)	0.16 (-7.01 to 7.34)	-0.23 (-0.83 to 0.36)	1.77 (0.41 to 3.13)	0.48 (-0.01 to 0.97)	2.73 (2.38 to 3.07)	**Placebo**					
-0.90 (-1.88 to 0.08)	-1.22 (-1.88 to -0.56)	-0.40 (-1.23 to 0.43)	-0.69 (-1.63 to 0.25)	-0.90 (-2.00 to 0.19)	1.45 (0.16 to 2.74)	-0.81 (-2.43 to 0.80)	-1.40 (-8.56 to 5.76)	-1.80 (-2.51 to -1.08)	0.20 (-1.22 to 1.62)	-1.08 (-1.73 to -0.44)	1.16 (0.63 to 1.69)	-1.56 (-2.04 to -1.09)	**Quetiapine**				
-0.62 (-1.51 to 0.27)	-0.94 (-1.45 to -0.43)	-0.12 (-0.86 to 0.62)	-0.41 (-1.27 to 0.45)	-0.62 (-1.63 to 0.39)	1.73 (0.50 to 2.96)	-0.53 (-2.09 to 1.03)	-1.12 (-8.30 to 6.06)	-1.52 (-2.12 to -0.91)	0.48 (-0.88 to 1.85)	-0.80 (-1.36 to -0.24)	1.44 (1.10 to 1.79)	-1.28 (-1.59 to -0.98)	0.28 (-0.22 to 0.78)	**Risp & Pali**			
-1.71 (-3.21 to -0.21)	-2.03 (-3.34 to -0.72)	-1.21 (-2.63 to 0.21)	-1.50 (-2.98 to -0.01)	-1.71 (-3.29 to -0.14)	0.64 (-1.08 to 2.37)	-1.62 (-3.60 to 0.36)	-2.21 (-9.49 to 5.07)	-2.61 (-3.96 to -1.25)	-0.61 (-2.43 to 1.22)	-1.89 (-3.22 to -0.56)	0.35 (-0.91 to 1.61)	-2.37 (-3.62 to -1.12)	-0.81 (-2.12 to 0.50)	-1.09 (-2.30 to 0.12)	**Sertindole**		
0.94 (-0.27 to 2.16)	0.62 (-0.37 to 1.62)	1.45 (0.34 to 2.55)	1.16 (-0.04 to 2.35)	0.94 (-0.38 to 2.26)	3.29 (1.81 to 4.78)	1.03 (-0.73 to 2.80)	0.44 (-6.78 to 7.67)	0.05 (-0.98 to 1.07)	2.05 (0.45 to 3.64)	0.76 (-0.18 to 1.71)	3.01 (2.12 to 3.89)	0.28 (-0.59 to 1.15)	1.84 (0.87 to 2.82)	1.56 (0.66 to 2.46)	2.65 (1.14 to 4.16)	**Ziprasidone**	
-2.14 (-4.05 to -0.23)	-2.46 (-4.25 to -0.67)	-1.64 (-3.46 to 0.19)	-1.93 (-3.84 to -0.02)	-2.14 (-4.13 to -0.15)	0.21 (-1.45 to 1.88)	-2.05 (-4.35 to 0.25)	-2.64 (-10.02 to 4.74)	-3.04 (-4.68 to -1.39)	-1.04 (-3.09 to 1.02)	-2.32 (-4.10 to -0.54)	-0.08 (-1.80 to 1.65)	-2.80 (-4.53 to -1.07)	-1.24 (-3.01 to 0.54)	-1.52 (-3.25 to 0.21)	-0.43 (-2.54 to 1.68)	-3.08 (-5.00 to -1.16)	**Zotepine**

eTable 6. League table for changes in body mass index (BMI, kg/m^2) associated with antipsychotic drug treatment. For interpretation, a number larger than zero favours the column-defining treatment of a cell, i.e. this treatment leads to an increase in BMI. Values depicted are mean differences with associated 95% confidence intervals.

**Aripiprazole**								
-1.24 (-2.21 to -0.28)	**Clozapine**							
0.03 (-0.70 to 0.77)	1.28 (0.50 to 2.06)	**Haloperidol**						
-0.46 (-1.08 to 0.15)	0.78 (0.01 to 1.55)	-0.50 (-0.95 to -0.05)	**Lurasidone**					
-1.29 (-1.91 to -0.67)	-0.05 (-0.80 to 0.70)	-1.33 (-1.73 to -0.93)	-0.83 (-1.05 to -0.61)	**Olanzapine**				
-0.22 (-0.81 to 0.37)	1.02 (0.27 to 1.78)	-0.25 (-0.68 to 0.17)	0.24 (0.08 to 0.41)	1.07 (0.90 to 1.25)	**Placebo**			
-0.92 (-1.57 to -0.27)	0.33 (-0.46 to 1.12)	-0.95 (-1.44 to -0.47)	-0.45 (-0.72 to -0.19)	0.38 (0.09 to 0.66)	-0.70 (-0.96 to -0.44)	**Quetiapine**		
-0.78 (-1.39 to -0.17)	0.46 (-0.29 to 1.21)	-0.82 (-1.23 to -0.41)	-0.32 (-0.52 to -0.12)	0.51 (0.37 to 0.65)	-0.56 (-0.70 to -0.42)	0.13 (-0.13 to 0.40)	**Risp & Pali**	
-0.98 (-1.78 to -0.19)	0.26 (-0.64 to 1.16)	-1.02 (-1.67 to -0.37)	-0.52 (-1.06 to 0.02)	0.31 (-0.21 to 0.83)	-0.76 (-1.29 to -0.24)	-0.07 (-0.63 to 0.50)	-0.20 (-0.70 to 0.30)	**Sertindole**

eTable 7. League table for changes in total cholesterol associated with antipsychotic drug treatment (mmol/L). For interpretation, a number larger than zero favours the column-defining treatment of a cell, i.e. this treatment leads to an increase in total cholesterol. Values depicted are mean differences with associated 95% confidence intervals.

**Amisulpride**													
0.16 (-0.41 to 0.74)	**Aripiprazole**												
0.16 (-0.42 to 0.74)	-0.01 (-0.15 to 0.14)	**Brexpiprazole**											
0.30 (-0.29 to 0.89)	0.14 (-0.03 to 0.30)	0.14 (-0.05 to 0.33)	**Cariprazine**										
-0.35 (-0.98 to 0.28)	-0.51 (-0.82 to -0.21)	-0.51 (-0.83 to -0.19)	-0.65 (-0.98 to -0.32)	**Clozapine**									
0.11 (-0.48 to 0.71)	-0.05 (-0.27 to 0.17)	-0.05 (-0.28 to 0.19)	-0.19 (-0.44 to 0.07)	0.46 (0.18 to 0.74)	**Haloperidol**								
0.31 (-0.31 to 0.94)	0.15 (-0.14 to 0.44)	0.15 (-0.15 to 0.46)	0.01 (-0.30 to 0.33)	0.66 (0.33 to 1.00)	0.20 (0.01 to 0.39)	**Iloperidone**							
0.25 (-0.33 to 0.82)	0.08 (-0.07 to 0.24)	0.09 (-0.08 to 0.25)	-0.05 (-0.25 to 0.14)	0.60 (0.28 to 0.91)	0.13 (-0.10 to 0.37)	-0.07 (-0.37 to 0.23)	**Lurasidone**						
-0.19 (-0.75 to 0.37)	-0.35 (-0.47 to -0.24)	-0.35 (-0.49 to -0.21)	-0.49 (-0.66 to -0.32)	0.16 (-0.13 to 0.45)	-0.30 (-0.50 to-0.11)	-0.50 (-0.77 to -0.23)	-0.44 (-0.57 to -0.30)	**Olanzapine**					
0.21 (-0.36 to 0.78)	0.05 (-0.05 to 0.15)	0.05 (-0.06 to 0.17)	-0.09 (-0.24 to 0.07)	0.56 (0.26 to 0.86)	0.10 (-0.11 to 0.31)	-0.10 (-0.38 to 0.18)	-0.03 (-0.15 to 0.09)	0.40 (0.31 to 0.49)	**Placebo**				
-0.09 (-0.67 to 0.49)	-0.26 (-0.41 to -0.10)	-0.25 (-0.41 to -0.09)	-0.39 (-0.59 to -0.20)	0.26 (-0.06 to 0.58)	-0.21 (-0.45 to 0.03)	-0.41 (-0.71 to -0.10)	-0.34 (-0.51 to -0.17)	0.10 (-0.05 to 0.24)	-0.31 (-0.42 to -0.19)	**Quetiapine**			
0.15 (-0.42 to 0.72)	-0.01 (-0.13 to 0.11)	-0.01 (-0.15 to 0.13)	-0.15 (-0.31 to 0.02)	0.50 (0.21 to 0.80)	0.04 (-0.17 to 0.24)	-0.16 (-0.44 to 0.12)	-0.09 (-0.24 to 0.05)	0.34 (0.25 to 0.43)	-0.06 (-0.15 to 0.03)	0.24 (0.10 to 0.39)	**Risp / Pali**		
0.28 (-0.35 to 0.91)	0.11 (-0.19 to 0.42)	0.12 (-0.19 to 0.43)	-0.02 (-0.34 to 0.30)	0.63 (0.22 to 1.03)	0.17 (-0.18 to 0.51)	-0.03 (-0.43 to 0.36)	0.03 (-0.28 to 0.34)	0.47 (0.18 to 0.76)	0.07 (-0.23 to 0.36)	0.37 (0.06 to 0.68)	0.13 (-0.15 to 0.40)	**Sertindole**	
0.25 (-0.32 to 0.83)	0.09 (-0.07 to 0.25)	0.10 (-0.08 to 0.27)	-0.05 (-0.25 to 0.16)	0.60 (0.29 to 0.92)	0.14 (-0.09 to 0.37)	-0.06 (-0.36 to 0.24)	0.01 (-0.14 to 0.16)	0.44 (0.31 to 0.58)	0.04 (-0.09 to 0.17)	0.35 (0.17 to 0.52)	0.10 (-0.04 to 0.25)	-0.02 (-0.34 to 0.29)	**Ziprasidone**

eTable 8. League table for changes in Low Density Lipoprotein (LDL) cholesterol associated with antipsychotic drug treatment (mmol/L). For interpretation, a number larger than zero favours the column-defining treatment of a cell, i.e. this treatment leads to an increase in LDL cholesterol. Values depicted are mean differences with associated 95% confidence intervals.

**Aripiprazole**								
-0.04 (-0.13 to 0.05)	**Brexpiprazole**							
0.13 (0.03 to 0.22)	0.16 (0.06 to 0.26)	**Cariprazine**						
0.05 (-0.06 to 0.16)	0.09 (-0.02 to 0.20)	-0.08 (-0.19 to 0.04)	**Lurasidone**					
-0.20 (-0.29 to -0.11)	-0.16 (-0.25 to -0.08)	-0.33 (-0.42 to -0.23)	-0.25 (-0.35 to -0.15)	**Olanzapine**				
0.00 (-0.07 to 0.07)	0.04 (-0.03 to 0.10)	-0.13 (-0.21 to -0.05)	-0.05 (-0.14 to 0.04)	0.20 (0.14 to 0.26)	**Placebo**			
-0.17 (-0.30 to -0.04)	-0.13 (-0.26 to -0.01)	-0.30 (-0.43 to -0.16)	-0.22 (-0.36 to -0.08)	0.03 (-0.10 to 0.15)	-0.17 (-0.28 to -0.06)	**Quetiapine**		
-0.01 (-0.10 to 0.08)	0.03 (-0.06 to 0.11)	-0.14 (-0.23 to -0.04)	-0.06 (-0.16 to 0.04)	0.19 (0.14 to 0.24)	-0.01 (-0.07 to 0.05)	0.16 (0.04 to 0.29)	**Risp / Pali**	
0.12 (-0.04 to 0.28)	0.16 (0.00 to 0.32)	-0.00 (-0.17 to 0.16)	0.07 (-0.10 to 0.24)	0.32 (0.18 to 0.46)	0.12 (-0.03 to 0.27)	0.29 (0.10 to 0.48)	0.13 (-0.02 to 0.28)	**Ziprasidone**

eFigure 9. League table for changes in High Density Lipoprotein (HDL) cholesterol associated with antipsychotic drug treatment (mmol/L). For interpretation, a number larger than zero favours the column-defining treatment of a cell, i.e. this treatment leads to an increase in HDL cholesterol. Values depicted are mean differences with associated 95% confidence intervals.

**Amisulpride**									
-0.14 (-0.37 to 0.10)	**Aripiprazole**								
-0.15 (-0.39 to 0.09)	-0.01 (-0.07 to 0.05)	**Brexpiprazole**							
-0.12 (-0.36 to 0.13)	0.02 (-0.04 to 0.08)	0.03 (-0.04 to 0.11)	**Cariprazine**						
-0.12 (-0.36 to 0.12)	0.02 (-0.06 to 0.10)	0.03 (-0.06 to 0.12)	-0.00 (-0.09 to 0.09)	**Lurasidone**					
-0.09 (-0.32 to 0.14)	0.05 (0.00 to 0.10)	0.06 (0.00 to 0.12)	0.03 (-0.04 to 0.09)	0.03 (-0.04 to 0.10)	**Olanzapine**				
-0.10 (-0.33 to 0.14)	0.04 (0.00 to 0.08)	0.05 (0.00 to 0.10)	0.02 (-0.04 to 0.08)	0.02 (-0.05 to 0.09)	-0.01 (-0.04 to 0.03)	**Placebo**			
-0.10 (-0.34 to 0.13)	0.03 (-0.03 to 0.09)	0.04 (-0.02 to 0.11)	0.01 (-0.06 to 0.08)	0.01 (-0.07 to 0.10)	-0.01 (-0.07 to 0.04)	-0.01 (-0.05 to 0.03)	**Quetiapine**		
-0.11 (-0.35 to 0.12)	0.03 (-0.02 to 0.07)	0.04 (-0.02 to 0.10)	0.01 (-0.05 to 0.07)	0.01 (-0.07 to 0.08)	-0.02 (-0.06 to 0.02)	-0.01 (-0.05 to 0.02)	-0.01 (-0.06 to 0.05)	**Risp / Pali**	
-0.14 (-0.39 to 0.10)	-0.01 (-0.09 to 0.07)	0.00 (-0.08 to 0.09)	-0.03 (-0.12 to 0.06)	-0.03 (-0.12 to 0.07)	-0.05 (-0.12 to 0.01)	-0.05 (-0.12 to 0.03)	-0.04 (-0.12 to 0.04)	-0.03 (-0.11 to 0.04)	**Ziprasidone**

eTable 10. League table for changes in triglycerides associated with antipsychotic drug treatment (mmol/L). For interpretation, a number larger than zero favours the column-defining treatment of a cell, i.e. this treatment leads to an increase in triglycerides. Values depicted are mean differences with associated 95% confidence intervals.

**Amisulpride**														
0.07 (-0.52 to 0.67)	**Aripiprazole**													
0.11 (-0.49 to 0.70)	0.03 (-0.09 to 0.16)	**Brexpiprazole**												
0.09 (-0.52 to 0.69)	0.02 (-0.13 to 0.16)	-0.02 (-0.18 to 0.15)	**Cariprazine**											
-0.89 (-1.66 to -0.12)	-0.96 (-1.47 to -0.45)	-1.00 (-1.51 to -0.48)	-0.98 (-1.50 to -0.46)	**Clozapine**										
-0.17 (-0.87 to 0.53)	-0.24 (-0.64 to 0.15)	-0.28 (-0.68 to 0.12)	-0.26 (-0.67 to 0.15)	0.72 (0.19 to 1.24)	**Haloperidol**									
-0.18 (-0.90 to 0.54)	-0.25 (-0.69 to 0.19)	-0.28 (-0.72 to 0.16)	-0.27 (-0.72 to 0.18)	0.71 (0.16 to 1.27)	-0.00 (-0.19 to 0.18)	**Iloperidone**								
0.10 (-0.51 to 0.70)	0.02 (-0.14 to 0.19)	-0.01 (-0.17 to 0.15)	0.01 (-0.18 to 0.20)	0.99 (0.47 to 1.51)	0.27 (-0.14 to 0.68)	0.27 (-0.18 to 0.72)	**Lurasidone**							
-0.37 (-0.95 to 0.21)	-0.44 (-0.55 to -0.33)	-0.48 (-0.60 to -0.35)	-0.46 (-0.61 to -0.30)	0.52 (0.02 to 1.02)	-0.20 (-0.58 to 0.18)	-0.19 (-0.62 to 0.23)	-0.47 (-0.62 to -0.31)	**Olanzapine**						
0.09 (-0.50 to 0.68)	0.02 (-0.07 to 0.11)	-0.01 (-0.10 to 0.08)	0.01 (-0.13 to 0.14)	0.98 (0.48 to 1.49)	0.27 (-0.13 to 0.66)	0.27 (-0.16 to 0.70)	-0.00 (-0.14 to 0.13)	0.46 (0.37 to 0.55)	**Placebo**					
-0.23 (-0.83 to 0.37)	-0.30 (-0.45 to -0.16)	-0.33 (-0.48 to -0.19)	-0.32 (-0.49 to -0.14)	0.66 (0.14 to 1.18)	-0.06 (-0.46 to 0.35)	-0.05 (-0.50 to 0.39)	-0.33 (-0.50 to -0.15)	0.14 (0.00 to 0.28)	-0.32 (-0.44 to -0.21)	**Quetiapine**				
0.05 (-0.54 to 0.65)	-0.02 (-0.13 to 0.09)	-0.05 (-0.17 to 0.07)	-0.03 (-0.18 to 0.11)	0.94 (0.44 to 1.45)	0.23 (-0.16 to 0.62)	0.23 (-0.20 to 0.66)	-0.04 (-0.20 to 0.11)	0.42 (0.34 to 0.51)	-0.04 (-0.12 to 0.04)	0.28 (0.14 to 0.42)	**Risp / Pali**			
0.09 (-0.58 to 0.76)	0.02 (-0.32 to 0.36)	-0.01 (-0.36 to 0.33)	0.00 (-0.35 to 0.35)	0.98 (0.38 to 1.58)	0.26 (-0.24 to 0.77)	0.27 (-0.27 to 0.80)	-0.01 (-0.36 to 0.35)	0.46 (0.13 to 0.79)	-0.00 (-0.33 to 0.33)	0.32 (-0.03 to 0.67)	0.04 (-0.28 to 0.36)	**Sertindole**		
0.07 (-0.53 to 0.67)	-0.00 (-0.16 to 0.16)	-0.04 (-0.20 to 0.13)	-0.02 (-0.21 to 0.17)	0.96 (0.44 to 1.48)	0.24 (-0.17 to 0.65)	0.25 (-0.20 to 0.69)	-0.03 (-0.21 to 0.16)	0.44 (0.29 to 0.59)	-0.02 (-0.16 to 0.11)	0.30 (0.12 to 0.48)	0.02 (-0.14 to 0.17)	-0.02 (-0.37 to 0.33)	**Ziprasidone**	
-0.83 (-1.71 to 0.06)	-0.90 (-1.58 to -0.23)	-0.93 (-1.61 to -0.26)	-0.92 (-1.60 to -0.23)	0.06 (-0.38 to 0.50)	-0.66 (-1.34 to 0.03)	-0.65 (-1.36 to 0.06)	-0.93 (-1.61 to -0.24)	-0.46(-1.12 to 0.21)	-0.92 (-1.59 to -0.25)	-0.60 (-1.28 to 0.08)	-0.88 (-1.55 to -0.21)	-0.92 (-1.66 to -0.18)	-0.90 (-1.58 to -0.22)	**Zotepine**

eTable 11. League table for changes in glucose associated with antipsychotic drug treatment (mmol/L). For interpretation, a number larger than zero favours the column-defining treatment of a cell, i.e. this treatment leads to an increase in weight. Values depicted are mean differences with associated 95% confidence intervals.

**Amisulpride**															
-0.59 (-1.52 to 0.34)	**Aripiprazole**														
-0.31 (-1.33 to 0.70)	0.27 (-0.18 to 0.73)	**Asenapine**													
-0.50 (-1.45 to 0.46)	0.09 (-0.20 to 0.38)	-0.18 (-0.67 to 0.31)	**Brexpiprazole**												
-0.72 (-1.69 to 0.25)	-0.13 (-0.45 to 0.20)	-0.40 (-0.92 to 0.12)	-0.22 (-0.62 to 0.18)	**Cariprazine**											
-1.51 (-2.62 to -0.40)	-0.92 (-1.58 to -0.26)	-1.20 (-1.96 to -0.43)	-1.01 (-1.70 to -0.32)	-0.79 (-1.50 to -0.09)	**Clozapine**										
-0.64 (-1.61 to 0.32)	-0.05 (-0.43 to 0.33)	-0.33 (-0.87 to 0.22)	-0.14 (-0.58 to 0.29)	0.08 (-0.38 to 0.54)	0.87 (0.23 to 1.51)	**Haloperidol**									
-0.81 (-1.85 to 0.23)	-0.22 (-0.76 to 0.32)	-0.50 (-1.17 to 0.17)	-0.32 (-0.90 to 0.27)	-0.09 (-0.70 to 0.51)	0.70 (-0.05 to 1.45)	-0.17 (-0.56 to 0.22)	**Iloperidone**								
-0.17 (-1.12 to 0.79)	0.42 (0.11 to 0.73)	0.15 (-0.34 to 0.64)	0.33 (-0.03 to 0.69)	0.55 (0.14 to 0.96)	1.34 (0.65 to 2.03)	0.47 (0.04 to 0.90)	0.64 (0.06 to 1.22)	**Lurasidone**							
-0.66 (-1.57 to 0.25)	-0.07 (-0.28 to 0.14)	-0.35 (-0.79 to 0.10)	-0.16 (-0.46 to 0.13)	0.06 (-0.28 to 0.40)	0.85 (0.22 to 1.48)	-0.02 (-0.34 to 0.31)	0.15 (-0.35 to 0.66)	**-0.49 (-0.79 to -0.20)**	**Olanzapine**						
-0.46 (-1.38 to 0.47)	0.13 (-0.05 to 0.31)	-0.14 (-0.56 to 0.27)	0.04 (-0.21 to 0.29)	0.26 (-0.05 to 0.58)	1.05 (0.41 to 1.70)	0.19 (-0.17 to 0.54)	0.36 (-0.17 to 0.88)	**-0.29 (-0.55 to -0.03)**	**0.20 (0.04 to 0.37)**	**Placebo**					
-0.54 (-1.49 to 0.40)	0.04 (-0.22 to 0.30)	-0.23 (-0.69 to 0.23)	-0.05 (-0.37 to 0.27)	0.17 (-0.20 to 0.54)	0.96 (0.29 to 1.64)	0.10 (-0.31 to 0.50)	0.27 (-0.29 to 0.83)	**-0.38 (-0.71 to -0.05)**	0.11 (-0.14 to 0.36)	-0.09 (-0.29 to 0.11)	**Quetiapine**				
-0.54 (-1.46 to 0.39)	0.05 (-0.15 to 0.25)	-0.22 (-0.66 to 0.22)	-0.04 (-0.33 to 0.24)	0.18 (-0.14 to 0.50)	0.97 (0.33 to 1.61)	0.10 (-0.25 to 0.45)	0.27 (-0.25 to 0.80)	-0.37 (-0.66 to -0.08)	0.12 (-0.04 to 0.29)	-0.08 (-0.22 to 0.06)	0.01 (-0.23 to 0.25)	**Risp / Pali**			
-0.43 (-1.51 to 0.66)	0.16 (-0.44 to 0.76)	-0.11 (-0.83 to 0.60)	0.07 (-0.56 to 0.70)	0.29 (-0.36 to 0.94)	1.08 (0.23 to 1.93)	0.21 (-0.45 to 0.88)	0.39 (-0.38 to 1.15)	-0.26 (-0.89 to 0.38)	0.23 (-0.35 to 0.82)	0.03 (-0.55 to 0.61)	0.12 (-0.49 to 0.73)	0.11 (-0.45 to 0.67)	**Sertindole**		
-0.50 (-1.49 to 0.48)	0.09 (-0.35 to 0.52)	-0.19 (-0.77 to 0.40)	-0.01 (-0.49 to 0.47)	0.21 (-0.29 to 0.72)	1.01 (0.27 to 1.74)	0.14 (-0.36 to 0.64)	0.31 (-0.32 to 0.94)	-0.34 (-0.81 to 0.14)	0.16 (-0.22 to 0.53)	-0.05 (-0.46 to 0.36)	0.04 (-0.41 to 0.50)	0.03 (-0.38 to 0.45)	-0.08 (-0.78 to 0.62)	**Ziprasidone**	
-1.44 (-2.66 to -0.22)	-0.85 (-1.68 to -0.02)	-1.13 (-2.05 to -0.21)	-0.95 (-1.80 to -0.09)	-0.73 (-1.60 to 0.15)	0.07 (-0.44 to 0.58)	-0.80 (-1.62 to 0.02)	-0.63 (-1.54 to 0.28)	-1.27 (-2.13 to -0.42)	-0.78 (-1.59 to 0.03)	-0.99 (-1.81 to -0.17)	-0.90 (-1.74 to -0.06)	-0.91 (-1.72 to -0.09)	-1.02 (-2.01 to -0.02)	-0.94 (-1.83 to -0.04)	**Zotepine**

eTable 12. P-score ranking: weight

	P-score (random)
clozapine	0.9242
olanzapine	0.8921
zotepine	0.8773
sertindole	0.8249
iloperidone	0.6960
quetiapine	0.6831
risp/pali	0.5929
asenapine	0.5507
brexpiprazole	0.4552
flupenthixol	0.4118
fluphenazine	0.3968
amisulpride	0.3819
cariprazine	0.3819
lurasidone	0.3240
aripiprazole	0.2727
placebo	0.1462
ziprasidone	0.0977
haloperidol	0.0907

eTable 13. P-score ranking: BMI

	P-score (random)
olanzapine	0.9279
clozapine	0.8509
sertindole	0.7174
quetiapine	0.6829
risp/pali	0.5603
lurasidone	0.3708
placebo	0.2065
aripiprazole	0.1080
haloperidol	0.0752

eTable 14. P-score ranking: total cholesterol

	P-score (random)
clozapine	0.9738
olanzapine	0.9066
quetiapine	0.8242
amisulpride	0.6451
haloperidol	0.5906
risp/pali	0.5509
brexpiprazole	0.5165
aripiprazole	0.5063
placebo	0.3517
lurasidone	0.2723
sertindole	0.2560
ziprasidone	0.2519
iloperidone	0.1899
cariprazine	0.1641

eTable 15. P-score ranking: LDL cholesterol

	P-score (random)
olanzapine	0.9587
quetiapine	0.9121
brexpiprazole	0.6653
risp/pali	0.5388
aripiprazole	0.4836
placebo	0.4766
lurasidone	0.2735
ziprasidone	0.1173
cariprazine	0.0740

eTable 16. P-score ranking: HDL cholesterol (reversed for clinical interpretation)

	P-score (random)
amisulpride	0.8316
olanzapine	0.7642
placebo	0.7024
quetiapine	0.5900
risp/pali	0.5124
cariprazine	0.4678
lurasidone	0.4490
aripiprazole	0.2592
ziprasidone	0.2413
brexpiprazole	0.1821

eTable 17. P-score ranking: triglycerides

	P-score (random)
clozapine	0.9685
zotepine	0.9375
olanzapine	0.8309
quetiapine	0.7148
haloperidol	0.6317
iloperidone	0.6290
amisulpride	0.4189
risp/pali	0.3912
aripiprazole	0.3324
ziprasidone	0.3313
sertindole	0.2950
cariprazine	0.2811
lurasidone	0.2597
placebo	0.2523
brexpiprazole	0.2257

eTable 18: P-score ranking: glucose

	P-score (random)
clozapine	0.9680
zotepine	0.9375
iloperidone	0.7339
cariprazine	0.6961
olanzapine	0.6668
haloperidol	0.5907
aripiprazole	0.5477
quetiapine	0.4745
risp/pali	0.4557
ziprasidone	0.4198
brexpiprazole	0.4000

sertindole	0.3556
placebo	0.3068
asenapine	0.2234
amisulpride	0.1351
lurasidone	0.0883

eTable 19. ‘Hot spots’ of inconsistency: weight

```
Random effects model
k          - Number of studies providing direct evidence
prop       - Direct evidence proportion
NMA        - Estimated treatment effect (MD) in network meta-analysis
direct     - Estimated treatment effect (MD) derived from direct evidence
indir.     - Estimated treatment effect (MD) derived from indirect evidence
Diff       - Difference between direct and indirect treatment estimates
z          - z-value of test for disagreement (direct versus indirect)
p-value    - p-value of test for disagreement (direct versus indirect)
```

Treatment Comparison	k	prop	NMA	Direct	Indir	Diff	z	p-value
amisulpride:aripiprazole	0	0	0.3192	.	0.3192	.	.	.
amisulpride:asenapine	0	0	-0.5025	.	-0.5025	.	.	.
amisulpride:brexpiprazole	0	0	-0.2128	.	-0.2128	.	.	.
amisulpride:cariprazine	0	0	0.0032	.	0.0032	.	.	.
amisulpride:clozapine	0	0	-2.3510	.	-2.3510	.	.	.
amisulpride:flupenthixol	1	0.60	-0.0891	-1.2000	1.5633	-2.7633	-1.77	0.0771
amisulpride:fluphenazine	0	0	0.4994	.	0.4994	.	.	.
amisulpride:haloperidol	0	0	0.8967	.	0.8967	.	.	.
amisulpride:iloperidone	0	0	-1.1033	.	-1.1033	.	.	.
amisulpride:lurasidone	0	0	0.1800	.	0.1800	.	.	.
amisulpride:olanzapine	2	0.76	-2.0634	-1.6085	-3.5338	1.9252	1.91	0.0566
amisulpride:placebo	0	0	0.6626	.	0.6626	.	.	.
amisulpride:quetiapine	0	0	-0.9006	.	-0.9006	.	.	.
amisulpride:risp_pali	1	0.18	-0.6208	-1.5000	-0.4321	-1.0679	-0.90	0.3686
amisulpride:sertindole	0	0	-1.7093	.	-1.7093	.	.	.
amisulpride:ziprasidone	0	0	0.9425	.	0.9425	.	.	.
amisulpride:zotepine	0	0	-2.1398	.	-2.1398	.	.	.
aripiprazole:asenapine	0	0	-0.8218	.	-0.8218	.	.	.
aripiprazole:brexpiprazole	1	0.42	-0.5320	-0.6379	-0.4560	-0.1819	-0.20	0.8447
aripiprazole:cariprazine	1	0.68	-0.3160	-0.3723	-0.1952	-0.1771	-0.16	0.8761
aripiprazole:clozapine	0	0	-2.6702	.	-2.6702	.	.	.
aripiprazole:flupenthixol	0	0	-0.4084	.	-0.4084	.	.	.
aripiprazole:fluphenazine	0	0	0.1801	.	0.1801	.	.	.
aripiprazole:haloperidol	0	0	0.5774	.	0.5774	.	.	.
aripiprazole:iloperidone	0	0	-1.4226	.	-1.4226	.	.	.
aripiprazole:lurasidone	0	0	-0.1393	.	-0.1393	.	.	.
aripiprazole:olanzapine	2	0.14	-2.3826	-2.6374	-2.3414	-0.2960	-0.36	0.7181
aripiprazole:placebo	5	0.57	0.3434	0.3123	0.3851	-0.0728	-0.14	0.8877
aripiprazole:quetiapine	1	0.09	-1.2199	-1.7000	-1.1720	-0.5280	-0.45	0.6509
aripiprazole:risp_pali	6	0.57	-0.9400	-0.7095	-1.2456	0.5360	1.03	0.3039
aripiprazole:sertindole	0	0	-2.0285	.	-2.0285	.	.	.
aripiprazole:ziprasidone	0	0	0.6232	.	0.6232	.	.	.
aripiprazole:zotepine	0	0	-2.4590	.	-2.4590	.	.	.
asenapine:brexpiprazole	0	0	0.2897	.	0.2897	.	.	.
asenapine:cariprazine	0	0	0.5058	.	0.5058	.	.	.
asenapine:clozapine	0	0	-1.8484	.	-1.8484	.	.	.
asenapine:flupenthixol	0	0	0.4134	.	0.4134	.	.	.
asenapine:fluphenazine	0	0	1.0019	.	1.0019	.	.	.
asenapine:haloperidol	1	0.42	1.3992	0.3509	2.1635	-1.8126	-2.13	0.0332
asenapine:iloperidone	0	0	-0.6008	.	-0.6008	.	.	.
asenapine:lurasidone	0	0	0.6825	.	0.6825	.	.	.
asenapine:olanzapine	1	0.24	-1.5608	-1.1000	-1.7025	0.6025	0.68	0.4980
asenapine:placebo	3	0.85	1.1652	1.2392	0.7587	0.4804	0.49	0.6256
asenapine:quetiapine	0	0	-0.3981	.	-0.3981	.	.	.
asenapine:risp_pali	0	0	-0.1182	.	-0.1182	.	.	.
asenapine:sertindole	0	0	-1.2067	.	-1.2067	.	.	.
asenapine:ziprasidone	0	0	1.4450	.	1.4450	.	.	.
asenapine:zotepine	0	0	-1.6372	.	-1.6372	.	.	.
brexpiprazole:cariprazine	0	0	0.2160	.	0.2160	.	.	.
brexpiprazole:clozapine	0	0	-2.1382	.	-2.1382	.	.	.
brexpiprazole:flupenthixol	0	0	0.1237	.	0.1237	.	.	.
brexpiprazole:fluphenazine	0	0	0.7122	.	0.7122	.	.	.
brexpiprazole:haloperidol	0	0	1.1094	.	1.1094	.	.	.

brexpiprazole:iloperidone	0	0	-0.8906	.	-0.8906	.	.	.
brexpiprazole:lurasidone	0	0	0.3928	.	0.3928	.	.	.
brexpiprazole:olanzapine	0	0	-1.8506	.	-1.8506	.	.	.
brexpiprazole:placebo	2	0.89	0.8754	0.8321	1.2214	-0.3893	-0.29	0.7694
brexpiprazole:quetiapine	0	0	-0.6878	.	-0.6878	.	.	.
brexpiprazole:risp_pali	0	0	-0.4080	.	-0.4080	.	.	.
brexpiprazole:sertindole	0	0	-1.4965	.	-1.4965	.	.	.
brexpiprazole:ziprasidone	0	0	1.1553	.	1.1553	.	.	.
brexpiprazole:zotepine	0	0	-1.9270	.	-1.9270	.	.	.
cariprazine:clozapine	0	0	-2.3542	.	-2.3542	.	.	.
cariprazine:flupenthixol	0	0	-0.0924	.	-0.0924	.	.	.
cariprazine:fluphenazine	0	0	0.4961	.	0.4961	.	.	.
cariprazine:haloperidol	0	0	0.8934	.	0.8934	.	.	.
cariprazine:iloperidone	0	0	-1.1066	.	-1.1066	.	.	.
cariprazine:lurasidone	0	0	0.1767	.	0.1767	.	.	.
cariprazine:olanzapine	0	0	-2.0666	.	-2.0666	.	.	.
cariprazine:placebo	1	0.71	0.6594	0.7723	0.3836	0.3887	0.34	0.7307
cariprazine:quetiapine	0	0	-0.9039	.	-0.9039	.	.	.
cariprazine:risp_pali	1	0.63	-0.6240	-0.8277	-0.2827	-0.5450	-0.51	0.6086
cariprazine:sertindole	0	0	-1.7125	.	-1.7125	.	.	.
cariprazine:ziprasidone	0	0	0.9393	.	0.9393	.	.	.
cariprazine:zotepine	0	0	-2.1430	.	-2.1430	.	.	.
clozapine:flupenthixol	0	0	2.2618	.	2.2618	.	.	.
clozapine:fluphenazine	0	0	2.8503	.	2.8503	.	.	.
clozapine:haloperidol	3	0.81	3.2476	3.9795	0.0352	3.9443	2.62	0.0088
clozapine:iloperidone	0	0	1.2476	.	1.2476	.	.	.
clozapine:lurasidone	0	0	2.5309	.	2.5309	.	.	.
clozapine:olanzapine	2	0.42	0.2876	-1.2115	1.3815	-2.5930	-2.06	0.0399
clozapine:placebo	0	0	3.0136	.	3.0136	.	.	.
clozapine:quetiapine	0	0	1.4503	.	1.4503	.	.	.
clozapine:risp_pali	1	0.35	1.7302	1.9000	1.6398	0.2602	0.20	0.8436
clozapine:sertindole	0	0	0.6417	.	0.6417	.	.	.
clozapine:ziprasidone	0	0	3.2935	.	3.2935	.	.	.
clozapine:zotepine	1	0.55	0.2112	-0.6500	1.2570	-1.9070	-1.12	0.2638
flupenthixol:fluphenazine	0	0	0.5885	.	0.5885	.	.	.
flupenthixol:haloperidol	0	0	0.9858	.	0.9858	.	.	.
flupenthixol:iloperidone	0	0	-1.0142	.	-1.0142	.	.	.
flupenthixol:lurasidone	0	0	0.2691	.	0.2691	.	.	.
flupenthixol:olanzapine	1	0.48	-1.9742	-3.4000	-0.6367	-2.7633	-1.77	0.0771
flupenthixol:placebo	0	0	0.7518	.	0.7518	.	.	.
flupenthixol:quetiapine	0	0	-0.8115	.	-0.8115	.	.	.
flupenthixol:risp_pali	0	0	-0.5316	.	-0.5316	.	.	.
flupenthixol:sertindole	0	0	-1.6201	.	-1.6201	.	.	.
flupenthixol:ziprasidone	0	0	1.0316	.	1.0316	.	.	.
flupenthixol:zotepine	0	0	-2.0506	.	-2.0506	.	.	.
fluphenazine:haloperidol	0	0	0.3973	.	0.3973	.	.	.
fluphenazine:iloperidone	0	0	-1.6027	.	-1.6027	.	.	.
fluphenazine:lurasidone	0	0	-0.3194	.	-0.3194	.	.	.
fluphenazine:olanzapine	0	0	-2.5627	.	-2.5627	.	.	.
fluphenazine:placebo	0	0	0.1633	.	0.1633	.	.	.
fluphenazine:quetiapine	1	1.00	-1.4000	-1.4000	.	.	.	.
fluphenazine:risp_pali	0	0	-1.1201	.	-1.1201	.	.	.
fluphenazine:sertindole	0	0	-2.2087	.	-2.2087	.	.	.
fluphenazine:ziprasidone	0	0	0.4431	.	0.4431	.	.	.
fluphenazine:zotepine	0	0	-2.6391	.	-2.6391	.	.	.
haloperidol:iloperidone	1	1.00	-2.0000	-2.0000	.	.	.	.
haloperidol:lurasidone	1	0.20	-0.7167	-0.1616	-0.8539	0.6923	0.75	0.4555
haloperidol:olanzapine	5	0.52	-2.9600	-3.3484	-2.5365	-0.8119	-1.37	0.1721
haloperidol:placebo	2	0.30	-0.2340	0.0000	-0.3349	0.3349	0.51	0.6105
haloperidol:quetiapine	1	0.08	-1.7973	-1.9100	-1.7875	-0.1225	-0.09	0.9274
haloperidol:risp_pali	2	0.25	-1.5174	-1.9161	-1.3875	-0.5286	-0.74	0.4593
haloperidol:sertindole	0	0	-2.6059	.	-2.6059	.	.	.
haloperidol:ziprasidone	0	0	0.0458	.	0.0458	.	.	.
haloperidol:zotepine	1	0.59	-3.0364	-2.2500	-4.1570	1.9070	1.12	0.2638
iloperidone:lurasidone	0	0	1.2833	.	1.2833	.	.	.
iloperidone:olanzapine	0	0	-0.9600	.	-0.9600	.	.	.
iloperidone:placebo	0	0	1.7660	.	1.7660	.	.	.

iloperidone:quetiapine	0	0	0.2027	.	0.2027	.	.	.
iloperidone:risp_pali	0	0	0.4826	.	0.4826	.	.	.
iloperidone:sertindole	0	0	-0.6059	.	-0.6059	.	.	.
iloperidone:ziprasidone	0	0	2.0458	.	2.0458	.	.	.
iloperidone:zotepine	0	0	-1.0364	.	-1.0364	.	.	.
lurasidone:olanzapine	1	0.14	-2.2433	-3.1000	-2.1048	-0.9952	-1.19	0.2350
lurasidone:placebo	7	0.84	0.4827	0.6081	-0.1653	0.7734	1.14	0.2535
lurasidone:quetiapine	1	0.22	-1.0806	-0.4854	-1.2487	0.7632	0.96	0.3366
lurasidone:risp_pali	0	0	-0.8007	.	-0.8007	.	.	.
lurasidone:sertindole	0	0	-1.8892	.	-1.8892	.	.	.
lurasidone:ziprasidone	1	0.24	0.7625	0.3000	0.9108	-0.6108	-0.54	0.5863
lurasidone:zotepine	0	0	-2.3197	.	-2.3197	.	.	.
olanzapine:placebo	12	0.51	2.7260	2.5045	2.9582	-0.4537	-1.29	0.1981
olanzapine:quetiapine	3	0.25	1.1627	0.3738	1.4233	-1.0495	-1.67	0.0942
olanzapine:risp_pali	16	0.63	1.4426	1.3350	1.6231	-0.2881	-0.79	0.4283
olanzapine:sertindole	0	0	0.3541	.	0.3541	.	.	.
olanzapine:ziprasidone	2	0.40	3.0059	5.1485	1.5890	3.5595	3.86	0.0001
olanzapine:zotepine	0	0	-0.0764	.	-0.0764	.	.	.
placebo:quetiapine	5	0.56	-1.5633	-1.3576	-1.8250	0.4673	0.96	0.3376
placebo:risp_pali	18	0.66	-1.2834	-1.3015	-1.2480	-0.0535	-0.16	0.8700
placebo:sertindole	0	0	-2.3719	.	-2.3719	.	.	.
placebo:ziprasidone	1	0.45	0.2798	-1.3600	1.6002	-2.9602	-3.31	0.0009
placebo:zotepine	0	0	-2.8024	.	-2.8024	.	.	.
quetiapine:risp_pali	4	0.39	0.2799	0.5469	0.1088	0.4381	0.84	0.4029
quetiapine:sertindole	0	0	-0.8087	.	-0.8087	.	.	.
quetiapine:ziprasidone	0	0	1.8431	.	1.8431	.	.	.
quetiapine:zotepine	0	0	-1.2391	.	-1.2391	.	.	.
risp_pali:sertindole	2	1.00	-1.0885	-1.0885	.	.	.	.
risp_pali:ziprasidone	0	0	1.5633	.	1.5633	.	.	.
risp_pali:zotepine	0	0	-1.5190	.	-1.5190	.	.	.
sertindole:ziprasidone	0	0	2.6518	.	2.6518	.	.	.
sertindole:zotepine	0	0	-0.4305	.	-0.4305	.	.	.
ziprasidone:zotepine	0	0	-3.0822	.	-3.0822	.	.	.

eTable 20. ‘Hot spots’ of inconsistency: BMI

```
Random effects model
k          - Number of studies providing direct evidence
prop       - Direct evidence proportion
NMA        - Estimated treatment effect (MD) in network meta-analysis
direct     - Estimated treatment effect (MD) derived from direct evidence
indir.     - Estimated treatment effect (MD) derived from indirect evidence
Diff       - Difference between direct and indirect treatment estimates
z          - z-value of test for disagreement (direct versus indirect)
p-value    - p-value of test for disagreement (direct versus indirect)
```

Comparison	k	Prop	NMA	Direct	Indir	Diff	z	p-value
aripiprazole:clozapine	0	0	-1.2441	.	-1.2441	.	.	.
aripiprazole:haloperidol	0	0	0.0346	.	0.0346	.	.	.
aripiprazole:lurasidone	0	0	-0.4625	.	-0.4625	.	.	.
aripiprazole:olanzapine	0	0	-1.2936	.	-1.2936	.	.	.
aripiprazole:placebo	1	1.00	-0.2200	-0.2200	.	.	.	.
aripiprazole:quetiapine	0	0	-0.9173	.	-0.9173	.	.	.
aripiprazole:risp_pali	0	0	-0.7832	.	-0.7832	.	.	.
aripiprazole:sertindole	0	0	-0.9832	.	-0.9832	.	.	.
clozapine:haloperidol	1	0.73	1.2787	1.3000	1.2226	0.0774	0.09	0.9311
clozapine:lurasidone	0	0	0.7816	.	0.7816	.	.	.
clozapine:olanzapine	1	0.51	-0.0495	-0.4000	0.3151	-0.7151	-0.93	0.3498
clozapine:placebo	0	0	1.0241	.	1.0241	.	.	.
clozapine:quetiapine	0	0	0.3269	.	0.3269	.	.	.
clozapine:risp_pali	1	0.79	0.4609	0.6000	-0.0770	0.6770	0.72	0.4732
clozapine:sertindole	0	0	0.2609	.	0.2609	.	.	.
haloperidol:lurasidone	0	0	-0.4971	.	-0.4971	.	.	.
haloperidol:olanzapine	3	0.83	-1.3282	-1.4003	-0.9655	-0.4348	-0.79	0.4319
haloperidol:placebo	0	0	-0.2546	.	-0.2546	.	.	.
haloperidol:quetiapine	0	0	-0.9519	.	-0.9519	.	.	.
haloperidol:risp_pali	2	0.48	-0.8178	-0.6461	-0.9765	0.3303	0.79	0.4321
haloperidol:sertindole	0	0	-1.0178	.	-1.0178	.	.	.
lurasidone:olanzapine	1	0.31	-0.8311	-1.0538	-0.7290	-0.3248	-1.33	0.1839
lurasidone:placebo	4	0.91	0.2425	0.2734	-0.0647	0.3380	1.15	0.2509
lurasidone:quetiapine	1	0.64	-0.4547	-0.5000	-0.3753	-0.1247	-0.44	0.6598
lurasidone:risp_pali	0	0	-0.3207	.	-0.3207	.	.	.
lurasidone:sertindole	0	0	-0.5207	.	-0.5207	.	.	.
olanzapine:placebo	3	0.56	1.0736	1.0178	1.1449	-0.1270	-0.70	0.4853
olanzapine:quetiapine	0	0	0.3764	.	0.3764	.	.	.
olanzapine:risp_pali	9	0.87	0.5104	0.4778	0.7355	-0.2578	-1.17	0.2422
olanzapine:sertindole	0	0	0.3104	.	0.3104	.	.	.
placebo:quetiapine	1	0.49	-0.6973	-0.7000	-0.6946	-0.0054	-0.02	0.9836
placebo:risp_pali	7	0.83	-0.5632	-0.5451	-0.6523	0.1071	0.56	0.5752
placebo:sertindole	0	0	-0.7632	.	-0.7632	.	.	.
quetiapine:risp_pali	1	0.46	0.1341	0.0900	0.1718	-0.0818	-0.31	0.7600
quetiapine:sertindole	0	0	-0.0659	.	-0.0659	.	.	.
risp_pali:sertindole	1	1.00	-0.2000	-0.2000	.	.	.	.

eTable 21. 'Hot spots' of inconsistency: total cholesterol

```
Random effects model
k          - Number of studies providing direct evidence
prop       - Direct evidence proportion
NMA        - Estimated treatment effect (MD) in network meta-analysis
direct     - Estimated treatment effect (MD) derived from direct evidence
indir.     - Estimated treatment effect (MD) derived from indirect evidence
Diff       - Difference between direct and indirect treatment estimates
z          - z-value of test for disagreement (direct versus indirect)
p-value    - p-value of test for disagreement (direct versus indirect)
```

Comparison	k	prop	NMA	direct	indir	Diff	z	p-value
amisulpride:aripiprazole	0	0	0.1646	.	0.1646	.	.	.
amisulpride:brexpiprazole	0	0	0.1589	.	0.1589	.	.	.
amisulpride:cariprazine	0	0	0.3002	.	0.3002	.	.	.
amisulpride:clozapine	0	0	-0.3498	.	-0.3498	.	.	.
amisulpride:haloperidol	0	0	0.1129	.	0.1129	.	.	.
amisulpride:iloperidone	0	0	0.3123	.	0.3123	.	.	.
amisulpride:lurasidone	0	0	0.2456	.	0.2456	.	.	.
amisulpride:olanzapine	1	1.00	-0.1900	-0.1900	.	.	.	.
amisulpride:placebo	0	0	0.2126	.	0.2126	.	.	.
amisulpride:quetiapine	0	0	-0.0928	.	-0.0928	.	.	.
amisulpride:risp_pali	0	0	0.1514	.	0.1514	.	.	.
amisulpride:sertindole	0	0	0.2783	.	0.2783	.	.	.
amisulpride:ziprasidone	0	0	0.2543	.	0.2543	.	.	.
aripiprazole:brexpiprazole	1	0.27	-0.0057	-0.0723	0.0183	-0.0907	-0.55	0.5835
aripiprazole:cariprazine	1	0.61	0.1356	0.0558	0.2613	-0.2055	-1.17	0.2430
aripiprazole:clozapine	0	0	-0.5144	.	-0.5144	.	.	.
aripiprazole:haloperidol	0	0	-0.0518	.	-0.0518	.	.	.
aripiprazole:iloperidone	0	0	0.1477	.	0.1477	.	.	.
aripiprazole:lurasidone	0	0	0.0809	.	0.0809	.	.	.
aripiprazole:olanzapine	2	0.38	-0.3546	-0.1651	-0.4698	0.3047	2.50	0.0126
aripiprazole:placebo	4	0.69	0.0480	0.0006	0.1534	-0.1528	-1.35	0.1755
aripiprazole:quetiapine	1	0.08	-0.2575	-0.2124	-0.2612	0.0489	0.17	0.8681
aripiprazole:risp_pali	3	0.29	-0.0132	-0.1947	0.0610	-0.2556	-1.89	0.0586
aripiprazole:sertindole	0	0	0.1137	.	0.1137	.	.	.
aripiprazole:ziprasidone	0	0	0.0897	.	0.0897	.	.	.
brexpiprazole:cariprazine	0	0	0.1414	.	0.1414	.	.	.
brexpiprazole:clozapine	0	0	-0.5087	.	-0.5087	.	.	.
brexpiprazole:haloperidol	0	0	-0.0460	.	-0.0460	.	.	.
brexpiprazole:iloperidone	0	0	0.1534	.	0.1534	.	.	.
brexpiprazole:lurasidone	0	0	0.0867	.	0.0867	.	.	.
brexpiprazole:olanzapine	0	0	-0.3489	.	-0.3489	.	.	.
brexpiprazole:placebo	3	0.92	0.0538	0.0420	0.1890	-0.1470	-0.69	0.4875
brexpiprazole:quetiapine	0	0	-0.2517	.	-0.2517	.	.	.
brexpiprazole:risp_pali	0	0	-0.0075	.	-0.0075	.	.	.
brexpiprazole:sertindole	0	0	0.1195	.	0.1195	.	.	.
brexpiprazole:ziprasidone	0	0	0.0954	.	0.0954	.	.	.
cariprazine:clozapine	0	0	-0.6501	.	-0.6501	.	.	.
cariprazine:haloperidol	0	0	-0.1874	.	-0.1874	.	.	.
cariprazine:iloperidone	0	0	0.0120	.	0.0120	.	.	.
cariprazine:lurasidone	0	0	-0.0547	.	-0.0547	.	.	.
cariprazine:olanzapine	0	0	-0.4902	.	-0.4902	.	.	.
cariprazine:placebo	1	0.76	-0.0876	-0.1154	-0.0011	-0.1143	-0.61	0.5388
cariprazine:quetiapine	0	0	-0.3931	.	-0.3931	.	.	.
cariprazine:risp_pali	1	0.55	-0.1488	-0.1828	-0.1071	-0.0757	-0.45	0.6558
cariprazine:sertindole	0	0	-0.0219	.	-0.0219	.	.	.
cariprazine:ziprasidone	0	0	-0.0459	.	-0.0459	.	.	.
clozapine:haloperidol	2	0.94	0.4627	0.4910	0.0128	0.4782	0.79	0.4278
clozapine:iloperidone	0	0	0.6621	.	0.6621	.	.	.
clozapine:lurasidone	0	0	0.5954	.	0.5954	.	.	.
clozapine:olanzapine	2	0.79	0.1598	0.1800	0.0849	0.0950	0.26	0.7919
clozapine:placebo	0	0	0.5625	.	0.5625	.	.	.

clozapine:quetiapine	0	0	0.2570	.	0.2570	.	.	.
clozapine:risp_pali	1	0.41	0.5013	0.2720	0.6583	-0.3864	-1.26	0.2091
clozapine:sertindole	0	0	0.6282	.	0.6282	.	.	.
clozapine:ziprasidone	0	0	0.6041	.	0.6041	.	.	.
haloperidol:iloperidone	1	1.00	0.1994	0.1994	.	.	.	.
haloperidol:lurasidone	0	0	0.1327	.	0.1327	.	.	.
haloperidol:olanzapine	3	0.89	-0.3029	-0.2584	-0.6713	0.4130	1.30	0.1938
haloperidol:placebo	0	0	0.0998	.	0.0998	.	.	.
haloperidol:quetiapine	0	0	-0.2057	.	-0.2057	.	.	.
haloperidol:risp_pali	1	0.26	0.0386	-0.2357	0.1356	-0.3713	-1.55	0.1207
haloperidol:sertindole	0	0	0.1655	.	0.1655	.	.	.
haloperidol:ziprasidone	0	0	0.1415	.	0.1415	.	.	.
iloperidone:lurasidone	0	0	-0.0667	.	-0.0667	.	.	.
iloperidone:olanzapine	0	0	-0.5023	.	-0.5023	.	.	.
iloperidone:placebo	0	0	-0.0996	.	-0.0996	.	.	.
iloperidone:quetiapine	0	0	-0.4051	.	-0.4051	.	.	.
iloperidone:risp_pali	0	0	-0.1609	.	-0.1609	.	.	.
iloperidone:sertindole	0	0	-0.0339	.	-0.0339	.	.	.
iloperidone:ziprasidone	0	0	-0.0580	.	-0.0580	.	.	.
lurasidone:olanzapine	1	0.28	-0.4356	-0.4558	-0.4275	-0.0284	-0.18	0.8539
lurasidone:placebo	3	0.75	-0.0329	-0.0093	-0.1031	0.0938	0.66	0.5121
lurasidone:quetiapine	0	0	-0.3384	.	-0.3384	.	.	.
lurasidone:risp_pali	0	0	-0.0941	.	-0.0941	.	.	.
lurasidone:sertindole	0	0	0.0328	.	0.0328	.	.	.
lurasidone:ziprasidone	1	0.37	0.0088	-0.0518	0.0447	-0.0965	-0.59	0.5522
olanzapine:placebo	5	0.46	0.4026	0.4246	0.3836	0.0410	0.45	0.6557
olanzapine:quetiapine	1	0.06	0.0972	-0.0026	0.1038	-0.1064	-0.34	0.7303
olanzapine:risp_pali	6	0.70	0.3414	0.3788	0.2539	0.1250	1.27	0.2050
olanzapine:sertindole	0	0	0.4683	.	0.4683	.	.	.
olanzapine:ziprasidone	3	0.54	0.4443	0.4997	0.3798	0.1199	0.89	0.3742
placebo:quetiapine	4	0.93	-0.3055	-0.3054	-0.3069	0.0015	0.01	0.9951
placebo:risp_pali	6	0.71	-0.0612	-0.0521	-0.0831	0.0309	0.31	0.7552
placebo:sertindole	0	0	0.0657	.	0.0657	.	.	.
placebo:ziprasidone	1	0.37	0.0417	0.0057	0.0632	-0.0575	-0.41	0.6820
quetiapine:risp_pali	1	0.04	0.2443	0.1735	0.2475	-0.0740	-0.20	0.8398
quetiapine:sertindole	0	0	0.3712	.	0.3712	.	.	.
quetiapine:ziprasidone	0	0	0.3472	.	0.3472	.	.	.
risp_pali:sertindole	1	1.00	0.1269	0.1269	.	.	.	.
risp_pali:ziprasidone	0	0	0.1029	.	0.1029	.	.	.
sertindole:ziprasidone	0	0	-0.0240	.	-0.0240	.	.	.

eTable 22. 'Hot spots' of inconsistency: LDL cholesterol

```
Random effects model
k          - Number of studies providing direct evidence
prop       - Direct evidence proportion
NMA        - Estimated treatment effect (MD) in network meta-analysis
direct     - Estimated treatment effect (MD) derived from direct evidence
indir.     - Estimated treatment effect (MD) derived from indirect evidence
Diff       - Difference between direct and indirect treatment estimates
z          - z-value of test for disagreement (direct versus indirect)
p-value    - p-value of test for disagreement (direct versus indirect)
```

Comparison	k	prop	NMA	direct	indir	Diff	z	p-value
aripiprazole:brexpiprazole	1	0.21	-0.0369	-0.0324	-0.0382	0.0058	0.05	0.9598
aripiprazole:cariprazine	1	0.50	0.1259	0.1062	0.1460	-0.0397	-0.41	0.6835
aripiprazole:lurasidone	0	0	0.0508	.	0.0508	.	.	.
aripiprazole:olanzapine	2	0.32	-0.1996	-0.1461	-0.2251	0.0790	0.83	0.4084
aripiprazole:placebo	3	0.76	0.0003	-0.0058	0.0191	-0.0249	-0.29	0.7745
aripiprazole:quetiapine	0	0	-0.1713	.	-0.1713	.	.	.
aripiprazole:risp_pali	1	0.22	-0.0096	-0.0492	0.0018	-0.0510	-0.47	0.6366
aripiprazole:ziprasidone	0	0	0.1210	.	0.1210	.	.	.
brexpiprazole:cariprazine	0	0	0.1629	.	0.1629	.	.	.
brexpiprazole:lurasidone	0	0	0.0878	.	0.0878	.	.	.
brexpiprazole:olanzapine	1	0.26	-0.1626	-0.0790	-0.1926	0.1136	1.18	0.2381
brexpiprazole:placebo	2	0.79	0.0372	0.0213	0.0954	-0.0740	-0.91	0.3642
brexpiprazole:quetiapine	0	0	-0.1344	.	-0.1344	.	.	.
brexpiprazole:risp_pali	0	0	0.0274	.	0.0274	.	.	.
brexpiprazole:ziprasidone	0	0	0.1579	.	0.1579	.	.	.
cariprazine:lurasidone	0	0	-0.0751	.	-0.0751	.	.	.
cariprazine:olanzapine	0	0	-0.3255	.	-0.3255	.	.	.
cariprazine:placebo	1	0.78	-0.1257	-0.1269	-0.1211	-0.0059	-0.06	0.9526
cariprazine:quetiapine	0	0	-0.2973	.	-0.2973	.	.	.
cariprazine:risp_pali	1	0.39	-0.1355	-0.1554	-0.1229	-0.0325	-0.34	0.7352
cariprazine:ziprasidone	0	0	-0.0050	.	-0.0050	.	.	.
lurasidone:olanzapine	1	0.34	-0.2504	-0.2347	-0.2586	0.0239	0.22	0.8258
lurasidone:placebo	3	0.86	-0.0506	-0.0561	-0.0180	-0.0381	-0.30	0.7679
lurasidone:quetiapine	0	0	-0.2222	.	-0.2222	.	.	.
lurasidone:risp_pali	0	0	-0.0604	.	-0.0604	.	.	.
lurasidone:ziprasidone	0	0	0.0701	.	0.0701	.	.	.
olanzapine:placebo	6	0.47	0.1998	0.2249	0.1780	0.0469	0.73	0.4648
olanzapine:quetiapine	0	0	0.0282	.	0.0282	.	.	.
olanzapine:risp_pali	5	0.85	0.1900	0.2003	0.1335	0.0668	0.97	0.3312
olanzapine:ziprasidone	2	1.00	0.3205	0.3205	.	.	.	.
placebo:quetiapine	3	1.00	-0.1716	-0.1716	.	.	.	.
placebo:risp_pali	6	0.69	-0.0098	-0.0329	0.0411	-0.0740	-1.11	0.2681
placebo:ziprasidone	0	0	0.1207	.	0.1207	.	.	.
quetiapine:risp_pali	0	0	0.1618	.	0.1618	.	.	.
quetiapine:ziprasidone	0	0	0.2923	.	0.2923	.	.	.
risp_pali:ziprasidone	0	0	0.1305	.	0.1305	.	.	.

eTable 23. 'Hot spots' of inconsistency: HDL cholesterol

```
Random effects model
k          - Number of studies providing direct evidence
prop       - Direct evidence proportion
NMA        - Estimated treatment effect (MD) in network meta-analysis
direct     - Estimated treatment effect (MD) derived from direct evidence
indir.     - Estimated treatment effect (MD) derived from indirect evidence
Diff       - Difference between direct and indirect treatment estimates
z          - z-value of test for disagreement (direct versus indirect)
p-value    - p-value of test for disagreement (direct versus indirect)
```

comparison	k	prop	NMA	direct	indir	Diff	z	p-value
amisulpride:aripiprazole	0	0	-0.1359	.	-0.1359	.	.	.
amisulpride:brexpiprazole	0	0	-0.1479	.	-0.1479	.	.	.
amisulpride:cariprazine	0	0	-0.1163	.	-0.1163	.	.	.
amisulpride:lurasidone	0	0	-0.1186	.	-0.1186	.	.	.
amisulpride:olanzapine	1	1.00	-0.0900	-0.0900	.	.	.	.
amisulpride:placebo	0	0	-0.0969	.	-0.0969	.	.	.
amisulpride:quetiapine	0	0	-0.1044	.	-0.1044	.	.	.
amisulpride:risp_pali	0	0	-0.1109	.	-0.1109	.	.	.
amisulpride:ziprasidone	0	0	-0.1442	.	-0.1442	.	.	.
aripiprazole:brexpiprazole	1	0.44	-0.0119	0.0022	-0.0233	0.0255	0.43	0.6668
aripiprazole:cariprazine	1	0.66	0.0196	0.0164	0.0257	-0.0092	-0.14	0.8908
aripiprazole:lurasidone	0	0	0.0173	.	0.0173	.	.	.
aripiprazole:olanzapine	1	0.13	0.0459	0.1968	0.0232	0.1736	2.27	0.0234
aripiprazole:placebo	4	0.87	0.0390	0.0231	0.1451	-0.1220	-2.03	0.0422
aripiprazole:quetiapine	0	0	0.0315	.	0.0315	.	.	.
aripiprazole:risp_pali	1	0.30	0.0250	0.0181	0.0279	-0.0098	-0.18	0.8550
aripiprazole:ziprasidone	0	0	-0.0083	.	-0.0083	.	.	.
brexpiprazole:cariprazine	0	0	0.0315	.	0.0315	.	.	.
brexpiprazole:lurasidone	0	0	0.0293	.	0.0293	.	.	.
brexpiprazole:olanzapine	0	0	0.0579	.	0.0579	.	.	.
brexpiprazole:placebo	2	0.88	0.0509	0.0533	0.0339	0.0194	0.25	0.8026
brexpiprazole:quetiapine	0	0	0.0435	.	0.0435	.	.	.
brexpiprazole:risp_pali	0	0	0.0369	.	0.0369	.	.	.
brexpiprazole:ziprasidone	0	0	0.0036	.	0.0036	.	.	.
cariprazine:lurasidone	0	0	-0.0023	.	-0.0023	.	.	.
cariprazine:olanzapine	0	0	0.0263	.	0.0263	.	.	.
cariprazine:placebo	1	0.75	0.0194	0.0198	0.0181	0.0018	0.03	0.9792
cariprazine:quetiapine	0	0	0.0119	.	0.0119	.	.	.
cariprazine:risp_pali	1	0.63	0.0054	0.0017	0.0118	-0.0101	-0.16	0.8747
cariprazine:ziprasidone	0	0	-0.0279	.	-0.0279	.	.	.
lurasidone:olanzapine	1	0.72	0.0286	0.0371	0.0064	0.0307	0.37	0.7115
lurasidone:placebo	1	0.75	0.0217	0.0137	0.0452	-0.0314	-0.37	0.7115
lurasidone:quetiapine	0	0	0.0142	.	0.0142	.	.	.
lurasidone:risp_pali	0	0	0.0077	.	0.0077	.	.	.
lurasidone:ziprasidone	0	0	-0.0256	.	-0.0256	.	.	.
olanzapine:placebo	6	0.83	-0.0069	-0.0025	-0.0280	0.0254	0.53	0.5985
olanzapine:quetiapine	0	0	-0.0144	.	-0.0144	.	.	.
olanzapine:risp_pali	4	0.70	-0.0209	-0.0219	-0.0186	-0.0033	-0.08	0.9372
olanzapine:ziprasidone	2	1.00	-0.0542	-0.0542	.	.	.	.
placebo:quetiapine	4	1.00	-0.0075	-0.0075	.	.	.	.
placebo:risp_pali	6	0.87	-0.0140	-0.0099	-0.0408	0.0309	0.64	0.5236
placebo:ziprasidone	0	0	-0.0473	.	-0.0473	.	.	.
quetiapine:risp_pali	0	0	-0.0065	.	-0.0065	.	.	.
quetiapine:ziprasidone	0	0	-0.0398	.	-0.0398	.	.	.
risp_pali:ziprasidone	0	0	-0.0333	.	-0.0333	.	.	.

eTable 24. 'Hot spots' of inconsistency: triglycerides

```
Random effects model
k          - Number of studies providing direct evidence
prop       - Direct evidence proportion
NMA        - Estimated treatment effect (MD) in network meta-analysis
direct     - Estimated treatment effect (MD) derived from direct evidence
indir.     - Estimated treatment effect (MD) derived from indirect evidence
Diff       - Difference between direct and indirect treatment estimates
z          - z-value of test for disagreement (direct versus indirect)
p-value    - p-value of test for disagreement (direct versus indirect)
```

Comparison	k	prop	NMA	direct	indir	Diff	z	p-value
amisulpride:aripiprazole	0	0	0.0722	.	0.0722	.	.	.
amisulpride:brexpiprazole	0	0	0.1054	.	0.1054	.	.	.
amisulpride:cariprazine	0	0	0.0887	.	0.0887	.	.	.
amisulpride:clozapine	0	0	-0.8898	.	-0.8898	.	.	.
amisulpride:haloperidol	0	0	-0.1722	.	-0.1722	.	.	.
amisulpride:iloperidone	0	0	-0.1770	.	-0.1770	.	.	.
amisulpride:lurasidone	0	0	0.0970	.	0.0970	.	.	.
amisulpride:olanzapine	1	1.00	-0.3700	-0.3700	.	.	.	.
amisulpride:placebo	0	0	0.0938	.	0.0938	.	.	.
amisulpride:quetiapine	0	0	-0.2289	.	-0.2289	.	.	.
amisulpride:risp_pali	0	0	0.0548	.	0.0548	.	.	.
amisulpride:sertindole	0	0	0.0910	.	0.0910	.	.	.
amisulpride:ziprasidone	0	0	0.0702	.	0.0702	.	.	.
amisulpride:zotepine	0	0	-0.8284	.	-0.8284	.	.	.
aripiprazole:brexpiprazole	1	0.25	0.0332	-0.0099	0.0477	-0.0575	-0.39	0.6933
aripiprazole:cariprazine	1	0.68	0.0165	-0.0843	0.2278	-0.3121	-1.96	0.0495
aripiprazole:clozapine	0	0	-0.9620	.	-0.9620	.	.	.
aripiprazole:haloperidol	0	0	-0.2444	.	-0.2444	.	.	.
aripiprazole:iloperidone	0	0	-0.2491	.	-0.2491	.	.	.
aripiprazole:lurasidone	0	0	0.0249	.	0.0249	.	.	.
aripiprazole:olanzapine	2	0.35	-0.4422	-0.2232	-0.5621	0.3389	2.85	0.0044
aripiprazole:placebo	4	0.71	0.0217	-0.0256	0.1373	-0.1628	-1.55	0.1203
aripiprazole:quetiapine	1	0.11	-0.3010	-0.0825	-0.3292	0.2467	1.07	0.2865
aripiprazole:risp_pali	2	0.31	-0.0173	-0.1936	0.0612	-0.2549	-2.06	0.0396
aripiprazole:sertindole	0	0	0.0189	.	0.0189	.	.	.
aripiprazole:ziprasidone	0	0	-0.0020	.	-0.0020	.	.	.
aripiprazole:zotepine	0	0	-0.9006	.	-0.9006	.	.	.
brexpiprazole:cariprazine	0	0	-0.0167	.	-0.0167	.	.	.
brexpiprazole:clozapine	0	0	-0.9952	.	-0.9952	.	.	.
brexpiprazole:haloperidol	0	0	-0.2777	.	-0.2777	.	.	.
brexpiprazole:iloperidone	0	0	-0.2824	.	-0.2824	.	.	.
brexpiprazole:lurasidone	0	0	-0.0084	.	-0.0084	.	.	.
brexpiprazole:olanzapine	0	0	-0.4754	.	-0.4754	.	.	.
brexpiprazole:placebo	3	0.92	-0.0116	-0.0143	0.0193	-0.0335	-0.19	0.8481
brexpiprazole:quetiapine	0	0	-0.3343	.	-0.3343	.	.	.
brexpiprazole:risp_pali	0	0	-0.0506	.	-0.0506	.	.	.
brexpiprazole:sertindole	0	0	-0.0144	.	-0.0144	.	.	.
brexpiprazole:ziprasidone	0	0	-0.0352	.	-0.0352	.	.	.
brexpiprazole:zotepine	0	0	-0.9338	.	-0.9338	.	.	.
cariprazine:clozapine	0	0	-0.9785	.	-0.9785	.	.	.
cariprazine:haloperidol	0	0	-0.2609	.	-0.2609	.	.	.
cariprazine:iloperidone	0	0	-0.2657	.	-0.2657	.	.	.
cariprazine:lurasidone	0	0	0.0084	.	0.0084	.	.	.
cariprazine:olanzapine	0	0	-0.4587	.	-0.4587	.	.	.
cariprazine:placebo	1	0.77	0.0052	-0.0412	0.1643	-0.2055	-1.23	0.2176
cariprazine:quetiapine	0	0	-0.3175	.	-0.3175	.	.	.
cariprazine:risp_pali	1	0.48	-0.0338	-0.0830	0.0122	-0.0951	-0.63	0.5296
cariprazine:sertindole	0	0	0.0024	.	0.0024	.	.	.
cariprazine:ziprasidone	0	0	-0.0185	.	-0.0185	.	.	.
cariprazine:zotepine	0	0	-0.9171	.	-0.9171	.	.	.
clozapine:haloperidol	1	1.00	0.7175	0.7176	.	.	.	.

clozapine:iloperidone	0	0	0.7128	.	0.7128	.	.	.
clozapine:lurasidone	0	0	0.9868	.	0.9868	.	.	.
clozapine:olanzapine	1	1.00	0.5198	0.5198	.	.	.	.
clozapine:placebo	0	0	0.9836	.	0.9836	.	.	.
clozapine:quetiapine	0	0	0.6609	.	0.6609	.	.	.
clozapine:risp_pali	0	0	0.9446	.	0.9446	.	.	.
clozapine:sertindole	0	0	0.9808	.	0.9808	.	.	.
clozapine:ziprasidone	0	0	0.9600	.	0.9600	.	.	.
clozapine:zotepine	1	1.00	0.0614	0.0614	.	.	.	.
haloperidol:iloperidone	1	1.00	-0.0047	-0.0047	.	.	.	.
haloperidol:lurasidone	0	0	0.2693	.	0.2693	.	.	.
haloperidol:olanzapine	1	1.00	-0.1977	-0.1978	.	.	.	.
haloperidol:placebo	0	0	0.2661	.	0.2661	.	.	.
haloperidol:quetiapine	0	0	-0.0566	.	-0.0566	.	.	.
haloperidol:risp_pali	0	0	0.2271	.	0.2271	.	.	.
haloperidol:sertindole	0	0	0.2633	.	0.2633	.	.	.
haloperidol:ziprasidone	0	0	0.2424	.	0.2424	.	.	.
haloperidol:zotepine	0	0	-0.6562	.	-0.6562	.	.	.
iloperidone:lurasidone	0	0	0.2740	.	0.2740	.	.	.
iloperidone:olanzapine	0	0	-0.1930	.	-0.1930	.	.	.
iloperidone:placebo	0	0	0.2708	.	0.2708	.	.	.
iloperidone:quetiapine	0	0	-0.0519	.	-0.0519	.	.	.
iloperidone:risp_pali	0	0	0.2318	.	0.2318	.	.	.
iloperidone:sertindole	0	0	0.2680	.	0.2680	.	.	.
iloperidone:ziprasidone	0	0	0.2472	.	0.2472	.	.	.
iloperidone:zotepine	0	0	-0.6514	.	-0.6514	.	.	.
lurasidone:olanzapine	1	0.20	-0.4670	-0.6416	-0.4233	-0.2183	-1.11	0.2685
lurasidone:placebo	3	0.91	-0.0032	0.0247	-0.2812	0.3060	1.30	0.1946
lurasidone:quetiapine	0	0	-0.3259	.	-0.3259	.	.	.
lurasidone:risp_pali	0	0	-0.0422	.	-0.0422	.	.	.
lurasidone:sertindole	0	0	-0.0060	.	-0.0060	.	.	.
lurasidone:ziprasidone	1	0.09	-0.0268	-0.2825	-0.0006	-0.2819	-0.87	0.3839
lurasidone:zotepine	0	0	-0.9255	.	-0.9255	.	.	.
olanzapine:placebo	5	0.49	0.4638	0.4505	0.4766	-0.0261	-0.29	0.7742
olanzapine:quetiapine	1	0.06	0.1411	0.1435	0.1410	0.0025	0.01	0.9932
olanzapine:risp_pali	5	0.71	0.4248	0.4479	0.3686	0.0793	0.81	0.4194
olanzapine:sertindole	0	0	0.4610	.	0.4610	.	.	.
olanzapine:ziprasidone	3	0.38	0.4402	0.5823	0.3512	0.2311	1.51	0.1305
olanzapine:zotepine	0	0	-0.4584	.	-0.4584	.	.	.
placebo:quetiapine	4	0.91	-0.3227	-0.3504	-0.0339	-0.3165	-1.53	0.1250
placebo:risp_pali	6	0.73	-0.0390	-0.0414	-0.0324	-0.0090	-0.09	0.9250
placebo:sertindole	0	0	-0.0028	.	-0.0028	.	.	.
placebo:ziprasidone	1	0.66	-0.0236	-0.0757	0.0795	-0.1552	-1.06	0.2904
placebo:zotepine	0	0	-0.9223	.	-0.9223	.	.	.
quetiapine:risp_pali	1	0.10	0.2837	-0.1853	0.3345	-0.5198	-2.18	0.0294
quetiapine:sertindole	0	0	0.3199	.	0.3199	.	.	.
quetiapine:ziprasidone	0	0	0.2991	.	0.2991	.	.	.
quetiapine:zotepine	0	0	-0.5996	.	-0.5996	.	.	.
risp_pali:sertindole	1	1.00	0.0362	0.0362	.	.	.	.
risp_pali:ziprasidone	0	0	0.0153	.	0.0153	.	.	.
risp_pali:zotepine	0	0	-0.8833	.	-0.8833	.	.	.
sertindole:ziprasidone	0	0	-0.0208	.	-0.0208	.	.	.
sertindole:zotepine	0	0	-0.9194	.	-0.9194	.	.	.
ziprasidone:zotepine	0	0	-0.8986	.	-0.8986	.	.	.

eTable 25. ‘Hot spots’ of inconsistency: glucose

```
Random effects model
k          - Number of studies providing direct evidence
prop       - Direct evidence proportion
NMA        - Estimated treatment effect (MD) in network meta-analysis
direct     - Estimated treatment effect (MD) derived from direct evidence
indir.     - Estimated treatment effect (MD) derived from indirect evidence
Diff       - Difference between direct and indirect treatment estimates
z          - z-value of test for disagreement (direct versus indirect)
p-value    - p-value of test for disagreement (direct versus indirect)
```

Comparison	k	prop	NMA	direct	indir	Diff	z	p-value
amisulpride:aripiprazole	0	0	-0.5888	.	-0.5888	.	.	.
amisulpride:asenapine	0	0	-0.3140	.	-0.3140	.	.	.
amisulpride:brexpiprazole	0	0	-0.4958	.	-0.4958	.	.	.
amisulpride:cariprazine	0	0	-0.7169	.	-0.7169	.	.	.
amisulpride:clozapine	0	0	-1.5091	.	-1.5091	.	.	.
amisulpride:haloperidol	0	0	-0.6407	.	-0.6407	.	.	.
amisulpride:iloperidone	0	0	-0.8111	.	-0.8111	.	.	.
amisulpride:lurasidone	0	0	-0.1677	.	-0.1677	.	.	.
amisulpride:olanzapine	1	1.00	-0.6593	-0.6593	.	.	.	.
amisulpride:placebo	0	0	-0.4556	.	-0.4556	.	.	.
amisulpride:quetiapine	0	0	-0.5448	.	-0.5448	.	.	.
amisulpride:risp_pali	0	0	-0.5368	.	-0.5368	.	.	.
amisulpride:sertindole	0	0	-0.4258	.	-0.4258	.	.	.
amisulpride:ziprasidone	0	0	-0.5032	.	-0.5032	.	.	.
amisulpride:zotepine	0	0	-1.4425	.	-1.4425	.	.	.
aripiprazole:asenapine	0	0	0.2747	.	0.2747	.	.	.
aripiprazole:brexpiprazole	1	0.41	0.0930	0.1175	0.0758	0.0416	0.14	0.8892
aripiprazole:cariprazine	1	0.74	-0.1281	-0.2666	0.2596	-0.5263	-1.40	0.1614
aripiprazole:clozapine	0	0	-0.9203	.	-0.9203	.	.	.
aripiprazole:haloperidol	0	0	-0.0519	.	-0.0519	.	.	.
aripiprazole:iloperidone	0	0	-0.2223	.	-0.2223	.	.	.
aripiprazole:lurasidone	0	0	0.4211	.	0.4211	.	.	.
aripiprazole:olanzapine	2	0.38	-0.0706	0.0311	-0.1321	0.1631	0.74	0.4567
aripiprazole:placebo	4	0.65	0.1331	0.0299	0.3267	-0.2968	-1.55	0.1220
aripiprazole:quetiapine	1	0.19	0.0439	0.5217	-0.0711	0.5928	1.76	0.0783
aripiprazole:risp_pali	3	0.40	0.0520	0.1207	0.0062	0.1145	0.55	0.5811
aripiprazole:sertindole	0	0	0.1630	.	0.1630	.	.	.
aripiprazole:ziprasidone	0	0	0.0856	.	0.0856	.	.	.
aripiprazole:zotepine	0	0	-0.8537	.	-0.8537	.	.	.
asenapine:brexpiprazole	0	0	-0.1817	.	-0.1817	.	.	.
asenapine:cariprazine	0	0	-0.4029	.	-0.4029	.	.	.
asenapine:clozapine	0	0	-1.1951	.	-1.1951	.	.	.
asenapine:haloperidol	0	0	-0.3267	.	-0.3267	.	.	.
asenapine:iloperidone	0	0	-0.4970	.	-0.4970	.	.	.
asenapine:lurasidone	0	0	0.1464	.	0.1464	.	.	.
asenapine:olanzapine	0	0	-0.3453	.	-0.3453	.	.	.
asenapine:placebo	1	1.00	-0.1416	-0.1416	.	.	.	.
asenapine:quetiapine	0	0	-0.2308	.	-0.2308	.	.	.
asenapine:risp_pali	0	0	-0.2227	.	-0.2227	.	.	.
asenapine:sertindole	0	0	-0.1117	.	-0.1117	.	.	.
asenapine:ziprasidone	0	0	-0.1892	.	-0.1892	.	.	.
asenapine:zotepine	0	0	-1.1285	.	-1.1285	.	.	.
brexpiprazole:cariprazine	0	0	-0.2211	.	-0.2211	.	.	.
brexpiprazole:clozapine	0	0	-1.0133	.	-1.0133	.	.	.
brexpiprazole:haloperidol	0	0	-0.1449	.	-0.1449	.	.	.
brexpiprazole:iloperidone	0	0	-0.3153	.	-0.3153	.	.	.
brexpiprazole:lurasidone	0	0	0.3281	.	0.3281	.	.	.
brexpiprazole:olanzapine	0	0	-0.1636	.	-0.1636	.	.	.
brexpiprazole:placebo	3	0.90	0.0401	0.0549	-0.0989	0.1538	0.35	0.7258
brexpiprazole:quetiapine	0	0	-0.0491	.	-0.0491	.	.	.
brexpiprazole:risp_pali	0	0	-0.0410	.	-0.0410	.	.	.
brexpiprazole:sertindole	0	0	0.0700	.	0.0700	.	.	.
brexpiprazole:ziprasidone	0	0	-0.0074	.	-0.0074	.	.	.

brexpiprazole:zotepine	0	0	-0.9467	.	-0.9467	.	.	.
cariprazine:clozapine	0	0	-0.7922	.	-0.7922	.	.	.
cariprazine:haloperidol	0	0	0.0762	.	0.0762	.	.	.
cariprazine:iloperidone	0	0	-0.0942	.	-0.0942	.	.	.
cariprazine:lurasidone	0	0	0.5492	.	0.5492	.	.	.
cariprazine:olanzapine	0	0	0.0576	.	0.0576	.	.	.
cariprazine:placebo	1	0.69	0.2613	-0.0053	0.8489	-0.8542	-2.47	0.0136
cariprazine:quetiapine	0	0	0.1721	.	0.1721	.	.	.
cariprazine:risp_pali	1	0.68	0.1801	0.3166	-0.1096	0.4262	1.22	0.2224
cariprazine:sertindole	0	0	0.2911	.	0.2911	.	.	.
cariprazine:ziprasidone	0	0	0.2137	.	0.2137	.	.	.
cariprazine:zotepine	0	0	-0.7256	.	-0.7256	.	.	.
clozapine:haloperidol	2	0.92	0.8684	0.6980	2.7408	-2.0428	-1.72	0.0852
clozapine:iloperidone	0	0	0.6980	.	0.6980	.	.	.
clozapine:lurasidone	0	0	1.3414	.	1.3414	.	.	.
clozapine:olanzapine	2	0.88	0.8498	0.9226	0.3263	0.5963	0.61	0.5444
clozapine:placebo	0	0	1.0535	.	1.0535	.	.	.
clozapine:quetiapine	0	0	0.9643	.	0.9643	.	.	.
clozapine:risp_pali	1	0.64	0.9723	1.0212	0.8870	0.1342	0.20	0.8428
clozapine:sertindole	0	0	1.0833	.	1.0833	.	.	.
clozapine:ziprasidone	0	0	1.0059	.	1.0059	.	.	.
clozapine:zotepine	1	1.00	0.0666	0.0666	.	.	.	.
haloperidol:iloperidone	1	1.00	-0.1704	-0.1704	.	.	.	.
haloperidol:lurasidone	0	0	0.4730	.	0.4730	.	.	.
haloperidol:olanzapine	3	0.92	-0.0186	-0.0554	0.3791	-0.4345	-0.73	0.4659
haloperidol:placebo	0	0	0.1851	.	0.1851	.	.	.
haloperidol:quetiapine	0	0	0.0959	.	0.0959	.	.	.
haloperidol:risp_pali	1	0.31	0.1039	0.5383	-0.0906	0.6289	1.63	0.1034
haloperidol:sertindole	0	0	0.2149	.	0.2149	.	.	.
haloperidol:ziprasidone	0	0	0.1375	.	0.1375	.	.	.
haloperidol:zotepine	0	0	-0.8018	.	-0.8018	.	.	.
iloperidone:lurasidone	0	0	0.6434	.	0.6434	.	.	.
iloperidone:olanzapine	0	0	0.1518	.	0.1518	.	.	.
iloperidone:placebo	0	0	0.3555	.	0.3555	.	.	.
iloperidone:quetiapine	0	0	0.2663	.	0.2663	.	.	.
iloperidone:risp_pali	0	0	0.2743	.	0.2743	.	.	.
iloperidone:sertindole	0	0	0.3853	.	0.3853	.	.	.
iloperidone:ziprasidone	0	0	0.3079	.	0.3079	.	.	.
iloperidone:zotepine	0	0	-0.6314	.	-0.6314	.	.	.
lurasidone:olanzapine	1	0.27	-0.4917	-0.5588	-0.4664	-0.0924	-0.28	0.7833
lurasidone:placebo	3	0.90	-0.2880	-0.2602	-0.5471	0.2869	0.64	0.5237
lurasidone:quetiapine	0	0	-0.3772	.	-0.3772	.	.	.
lurasidone:risp_pali	0	0	-0.3691	.	-0.3691	.	.	.
lurasidone:sertindole	0	0	-0.2581	.	-0.2581	.	.	.
lurasidone:ziprasidone	0	0	-0.3355	.	-0.3355	.	.	.
lurasidone:zotepine	0	0	-1.2748	.	-1.2748	.	.	.
olanzapine:placebo	6	0.58	0.2037	0.1924	0.2194	-0.0270	-0.16	0.8725
olanzapine:quetiapine	1	0.18	0.1145	0.1110	0.1153	-0.0043	-0.01	0.9896
olanzapine:risp_pali	6	0.67	0.1226	0.0677	0.2350	-0.1673	-0.94	0.3447
olanzapine:sertindole	0	0	0.2336	.	0.2336	.	.	.
olanzapine:ziprasidone	2	1.00	0.1561	0.1561	.	.	.	.
olanzapine:zotepine	0	0	-0.7832	.	-0.7832	.	.	.
placebo:quetiapine	4	0.84	-0.0892	-0.1420	0.1940	-0.3360	-1.18	0.2377
placebo:risp_pali	9	0.79	-0.0811	-0.0573	-0.1713	0.1140	0.65	0.5128
placebo:sertindole	0	0	0.0299	.	0.0299	.	.	.
placebo:ziprasidone	0	0	-0.0476	.	-0.0476	.	.	.
placebo:zotepine	0	0	-0.9869	.	-0.9869	.	.	.
quetiapine:risp_pali	1	0.14	0.0081	-0.3552	0.0683	-0.4235	-1.22	0.2242
quetiapine:sertindole	0	0	0.1191	.	0.1191	.	.	.
quetiapine:ziprasidone	0	0	0.0416	.	0.0416	.	.	.
quetiapine:zotepine	0	0	-0.8977	.	-0.8977	.	.	.
risp_pali:sertindole	1	1.00	0.1110	0.1110	.	.	.	.
risp_pali:ziprasidone	0	0	0.0336	.	0.0336	.	.	.
risp_pali:zotepine	0	0	-0.9057	.	-0.9057	.	.	.
sertindole:ziprasidone	0	0	-0.0774	.	-0.0774	.	.	.
sertindole:zotepine	0	0	-1.0167	.	-1.0167	.	.	.
ziprasidone:zotepine	0	0	-0.9393	.	-0.9393	.	.	.

eTable 26. CINeMA confidence rating for weight

Comparison	Number of studies	Within-study bias	Across-studies bias	Indirectness	Imprecision	Heterogeneity	Incoherence	Confidence rating
amisulpride:flupenthixol	1	Some concerns	Suspected	No concerns	Major concerns	No concerns	No concerns	Low
amisulpride:olanzapine	2	Some concerns	Suspected	No concerns	No concerns	No concerns	No concerns	Moderate
amisulpride:risp_pali	1	Some concerns	Suspected	No concerns	Major concerns	No concerns	No concerns	Low
aripiprazole:brexpiprazole	1	Major concerns	Suspected	No concerns	Major concerns	No concerns	No concerns	Very low
aripiprazole:cariprazine	1	Some concerns	Suspected	No concerns	Major concerns	No concerns	No concerns	Low
aripiprazole:olanzapine	2	Some concerns	Suspected	No concerns	No concerns	No concerns	No concerns	Moderate
aripiprazole:placebo	5	Some concerns	Suspected	No concerns	Major concerns	No concerns	No concerns	Low
aripiprazole:quetiapine	1	Some concerns	Suspected	No concerns	No concerns	Major concerns	No concerns	Low
aripiprazole:risp_pali	6	Some concerns	Suspected	No concerns	No concerns	Major concerns	No concerns	Low
asenapine:haloperidol	1	Some concerns	Suspected	No concerns	No concerns	Major concerns	Major concerns	Very low
asenapine:olanzapine	1	Some concerns	Suspected	No concerns	No concerns	No concerns	No concerns	Moderate
asenapine:placebo	3	Some concerns	Suspected	No concerns	No concerns	Major concerns	No concerns	Low
brexpiprazole:placebo	2	Major concerns	Suspected	No concerns	No concerns	Major concerns	No concerns	Very low
cariprazine:placebo	1	Some concerns	Suspected	No concerns	Major concerns	No concerns	No concerns	Low
cariprazine:risp_pali	1	Some concerns	Suspected	No concerns	Major concerns	No concerns	No concerns	Low
clozapine:haloperidol	3	Some concerns	Suspected	No concerns	No concerns	No concerns	Major concerns	Low
clozapine:olanzapine	2	Some concerns	Suspected	No concerns	Major concerns	No concerns	No concerns	Low
clozapine:risp_pali	1	Some concerns	Suspected	No concerns	No concerns	No concerns	No concerns	Moderate
clozapine:zotepine	1	Some concerns	Suspected	No concerns	Major concerns	No concerns	No concerns	Low
flupenthixol:olanzapine	1	Some concerns	Suspected	No concerns	No concerns	No concerns	Major concerns	Low

fluphenazine:quetiapine	1	Some concerns	Suspected	No concerns	Major concerns	No concerns	No concerns	Low
fluphenazine:risp_pali	1	Some concerns	Suspected	No concerns	Major concerns	No concerns	No concerns	Low
haloperidol:iloperidone	1	Some concerns	Suspected	No concerns	No concerns	No concerns	Major concerns	Low
haloperidol:lurasidone	1	Some concerns	Suspected	No concerns	Major concerns	No concerns	No concerns	Low
haloperidol:olanzapine	5	Some concerns	Suspected	No concerns	No concerns	No concerns	No concerns	Moderate
haloperidol:placebo	2	Some concerns	Suspected	No concerns	Major concerns	No concerns	No concerns	Low
haloperidol:quetiapine	1	Some concerns	Suspected	No concerns	No concerns	No concerns	No concerns	Moderate
haloperidol:risp_pali	2	Some concerns	Suspected	No concerns	No concerns	No concerns	No concerns	Moderate
haloperidol:zotepine	1	Some concerns	Suspected	No concerns	No concerns	No concerns	No concerns	Moderate
lurasidone:olanzapine	1	Some concerns	Suspected	No concerns	No concerns	No concerns	No concerns	Moderate
lurasidone:placebo	7	Some concerns	Suspected	No concerns	Major concerns	No concerns	No concerns	Low
lurasidone:quetiapine	1	Some concerns	Suspected	No concerns	No concerns	Major concerns	No concerns	Low
lurasidone:ziprasidone	1	Some concerns	Suspected	No concerns	Major concerns	No concerns	No concerns	Low
olanzapine:placebo	12	Some concerns	Suspected	No concerns	No concerns	No concerns	No concerns	Moderate
olanzapine:quetiapine	3	Some concerns	Suspected	No concerns	No concerns	Major concerns	Major concerns	Very low
olanzapine:risp_pali	16	Some concerns	Suspected	No concerns	No concerns	No concerns	No concerns	Moderate
olanzapine:ziprasidone	2	Some concerns	Suspected	No concerns	No concerns	No concerns	No concerns	Moderate
placebo:quetiapine	5	Some concerns	Suspected	No concerns	No concerns	No concerns	No concerns	Moderate
placebo:risp_pali	18	Some concerns	Undetected	No concerns	No concerns	No concerns	No concerns	High
placebo:ziprasidone	1	Some concerns	Suspected	No concerns	Major concerns	No concerns	Major concerns	Very low
quetiapine:risp_pali	5	Some concerns	Suspected	No concerns	Major concerns	No concerns	No concerns	Low
risp_pali:sertindole	2	Some concerns	Suspected	No concerns	Major concerns	No concerns	Major concerns	Very low
amisulpride:aripiprazole	0	Some concerns	Suspected	No concerns	Major concerns	No concerns	Major concerns	Very low

amisulpride:asenapine	0	Some concerns	Suspected	No concerns	Major concerns	No concerns	Major concerns	Very low
amisulpride:brexpiprazole	0	Some concerns	Suspected	No concerns	Major concerns	No concerns	Major concerns	Very low
amisulpride:cariprazine	0	Some concerns	Suspected	No concerns	Major concerns	No concerns	Major concerns	Very low
amisulpride:clozapine	0	Some concerns	Suspected	No concerns	No concerns	No concerns	Major concerns	Low
amisulpride:fluphenazine	0	Some concerns	Suspected	No concerns	Major concerns	No concerns	Major concerns	Very low
amisulpride:haloperidol	0	Some concerns	Suspected	No concerns	Major concerns	No concerns	Major concerns	Very low
amisulpride:iloperidone	0	Some concerns	Suspected	No concerns	Major concerns	No concerns	Major concerns	Very low
amisulpride:lurasidone	0	Some concerns	Suspected	No concerns	Major concerns	No concerns	Major concerns	Very low
amisulpride:placebo	0	Some concerns	Suspected	No concerns	Major concerns	No concerns	Major concerns	Very low
amisulpride:quetiapine	0	Some concerns	Suspected	No concerns	Major concerns	No concerns	Major concerns	Very low
amisulpride:sertindole	0	Some concerns	Suspected	No concerns	No concerns	Major concerns	Major concerns	Very low
amisulpride:ziprasidone	0	Some concerns	Suspected	No concerns	Major concerns	No concerns	Major concerns	Very low
amisulpride:zotepine	0	Some concerns	Suspected	No concerns	No concerns	Major concerns	Major concerns	Very low
aripiprazole:asenapine	0	Some concerns	Suspected	No concerns	Major concerns	No concerns	Major concerns	Very low
aripiprazole:clozapine	0	Some concerns	Suspected	No concerns	No concerns	No concerns	Major concerns	Low
aripiprazole:flupenthixol	0	Some concerns	Suspected	No concerns	Major concerns	No concerns	Major concerns	Very low
aripiprazole:fluphenazine	0	Some concerns	Suspected	No concerns	Major concerns	No concerns	Major concerns	Very low
aripiprazole:haloperidol	0	Some concerns	Suspected	No concerns	Major concerns	No concerns	Major concerns	Very low
aripiprazole:iloperidone	0	Some concerns	Suspected	No concerns	Major concerns	No concerns	Major concerns	Very low
aripiprazole:lurasidone	0	Some concerns	Suspected	No concerns	Major concerns	No concerns	Major concerns	Very low
aripiprazole:sertindole	0	Some concerns	Suspected	No concerns	No concerns	No concerns	Major concerns	Low
aripiprazole:ziprasidone	0	Some concerns	Suspected	No concerns	Major concerns	No concerns	Major concerns	Very low
aripiprazole:zotepine	0	Some concerns	Suspected	No concerns	No concerns	No concerns	Major concerns	Low

asenapine:brexpiprazole	0	Some concerns	Suspected	No concerns	Major concerns	No concerns	Major concerns	Very low
asenapine:cariprazine	0	Some concerns	Suspected	No concerns	Major concerns	No concerns	Major concerns	Very low
asenapine:clozapine	0	Some concerns	Suspected	No concerns	No concerns	No concerns	Major concerns	Low
asenapine:flupenthixol	0	Some concerns	Suspected	No concerns	Major concerns	No concerns	Major concerns	Very low
asenapine:fluphenazine	0	Some concerns	Suspected	No concerns	Major concerns	No concerns	Major concerns	Very low
asenapine:iloperidone	0	Some concerns	Suspected	No concerns	Major concerns	No concerns	Major concerns	Very low
asenapine:lurasidone	0	Some concerns	Suspected	No concerns	Major concerns	No concerns	Major concerns	Very low
asenapine:quetiapine	0	Some concerns	Suspected	No concerns	Major concerns	No concerns	Major concerns	Very low
asenapine:risp_pali	0	Some concerns	Suspected	No concerns	Major concerns	No concerns	Major concerns	Very low
asenapine:sertindole	0	Some concerns	Suspected	No concerns	Major concerns	No concerns	Major concerns	Very low
asenapine:ziprasidone	0	Some concerns	Suspected	No concerns	No concerns	Major concerns	Major concerns	Very low
asenapine:zotepine	0	Some concerns	Suspected	No concerns	Major concerns	No concerns	Major concerns	Very low
brexpiprazole:cariprazine	0	Some concerns	Suspected	No concerns	Major concerns	No concerns	Major concerns	Very low
brexpiprazole:clozapine	0	Some concerns	Suspected	No concerns	No concerns	No concerns	Major concerns	Low
brexpiprazole:flupenthixol	0	Some concerns	Suspected	No concerns	Major concerns	No concerns	Major concerns	Very low
brexpiprazole:fluphenazine	0	Some concerns	Suspected	No concerns	Major concerns	No concerns	Major concerns	Very low
brexpiprazole:haloperidol	0	Some concerns	Suspected	No concerns	No concerns	Major concerns	Major concerns	Very low
brexpiprazole:iloperidone	0	Some concerns	Suspected	No concerns	Major concerns	No concerns	Major concerns	Very low
brexpiprazole:lurasidone	0	Some concerns	Suspected	No concerns	Major concerns	No concerns	Major concerns	Very low
brexpiprazole:olanzapine	0	Some concerns	Suspected	No concerns	No concerns	No concerns	Major concerns	Low
brexpiprazole:quetiapine	0	Some concerns	Suspected	No concerns	Major concerns	No concerns	Major concerns	Very low
brexpiprazole:risp_pali	0	Some concerns	Suspected	No concerns	Major concerns	No concerns	Major concerns	Very low
brexpiprazole:sertindole	0	Some concerns	Suspected	No concerns	No concerns	Major concerns	Major concerns	Very low

brexpiprazole:ziprasidone	0	Major concerns	Suspected	No concerns	Major concerns	No concerns	Major concerns	Very low
brexpiprazole:zotepine	0	Some concerns	Suspected	No concerns	No concerns	Major concerns	Major concerns	Very low
cariprazine:clozapine	0	Some concerns	Suspected	No concerns	No concerns	No concerns	Major concerns	Low
cariprazine:flupenthixol	0	Some concerns	Suspected	No concerns	Major concerns	No concerns	Major concerns	Very low
cariprazine:fluphenazine	0	Some concerns	Suspected	No concerns	Major concerns	No concerns	Major concerns	Very low
cariprazine:haloperidol	0	Some concerns	Suspected	No concerns	Major concerns	No concerns	Major concerns	Very low
cariprazine:iloperidone	0	Some concerns	Suspected	No concerns	Major concerns	No concerns	Major concerns	Very low
cariprazine:lurasidone	0	Some concerns	Suspected	No concerns	Major concerns	No concerns	Major concerns	Very low
cariprazine:olanzapine	0	Some concerns	Suspected	No concerns	No concerns	No concerns	Major concerns	Low
cariprazine:quetiapine	0	Some concerns	Suspected	No concerns	Major concerns	No concerns	Major concerns	Very low
cariprazine:sertindole	0	Some concerns	Suspected	No concerns	No concerns	Major concerns	Major concerns	Very low
cariprazine:ziprasidone	0	Some concerns	Suspected	No concerns	Major concerns	No concerns	Major concerns	Very low
cariprazine:zotepine	0	Some concerns	Suspected	No concerns	No concerns	Major concerns	Major concerns	Very low
clozapine:flupenthixol	0	Some concerns	Suspected	No concerns	No concerns	Major concerns	Major concerns	Very low
clozapine:fluphenazine	0	Some concerns	Suspected	No concerns	Major concerns	No concerns	Major concerns	Very low
clozapine:iloperidone	0	Some concerns	Suspected	No concerns	Major concerns	No concerns	Major concerns	Very low
clozapine:lurasidone	0	Some concerns	Suspected	No concerns	No concerns	No concerns	Major concerns	Low
clozapine:placebo	0	Some concerns	Suspected	No concerns	No concerns	No concerns	Major concerns	Low
clozapine:quetiapine	0	Some concerns	Suspected	No concerns	No concerns	Major concerns	Major concerns	Very low
clozapine:sertindole	0	Some concerns	Suspected	No concerns	Major concerns	No concerns	Major concerns	Very low
clozapine:ziprasidone	0	Some concerns	Suspected	No concerns	No concerns	No concerns	Major concerns	Low
flupenthixol:fluphenazine	0	Some concerns	Suspected	No concerns	Major concerns	No concerns	Major concerns	Very low
flupenthixol:haloperidol	0	Some concerns	Suspected	No concerns	Major concerns	No concerns	Major concerns	Very low

flupenthixol:iloperidone	0	Some concerns	Suspected	No concerns	Major concerns	No concerns	Major concerns	Very low
flupenthixol:lurasidone	0	Some concerns	Suspected	No concerns	Major concerns	No concerns	Major concerns	Very low
flupenthixol:placebo	0	Some concerns	Suspected	No concerns	Major concerns	No concerns	Major concerns	Very low
flupenthixol:quetiapine	0	Some concerns	Suspected	No concerns	Major concerns	No concerns	Major concerns	Very low
flupenthixol:risp_pali	0	Some concerns	Suspected	No concerns	Major concerns	No concerns	Major concerns	Very low
flupenthixol:sertindole	0	Some concerns	Suspected	No concerns	Major concerns	No concerns	Major concerns	Very low
flupenthixol:ziprasidone	0	Some concerns	Suspected	No concerns	Major concerns	No concerns	Major concerns	Very low
flupenthixol:zotepine	0	Some concerns	Suspected	No concerns	Major concerns	No concerns	Major concerns	Very low
fluphenazine:haloperidol	0	Some concerns	Suspected	No concerns	Major concerns	No concerns	Major concerns	Very low
fluphenazine:iloperidone	0	Some concerns	Suspected	No concerns	Major concerns	No concerns	Major concerns	Very low
fluphenazine:lurasidone	0	Some concerns	Suspected	No concerns	Major concerns	No concerns	Major concerns	Very low
fluphenazine:olanzapine	0	Some concerns	Suspected	No concerns	Major concerns	No concerns	Major concerns	Very low
fluphenazine:placebo	0	Some concerns	Suspected	No concerns	Major concerns	No concerns	Major concerns	Very low
fluphenazine:sertindole	0	Some concerns	Suspected	No concerns	Major concerns	No concerns	Major concerns	Very low
fluphenazine:ziprasidone	0	Some concerns	Suspected	No concerns	Major concerns	No concerns	Major concerns	Very low
fluphenazine:zotepine	0	Some concerns	Suspected	No concerns	Major concerns	No concerns	Major concerns	Very low
haloperidol:sertindole	0	Some concerns	Suspected	No concerns	No concerns	No concerns	Major concerns	Low
haloperidol:ziprasidone	0	Some concerns	Suspected	No concerns	Major concerns	No concerns	Major concerns	Very low
iloperidone:lurasidone	0	Some concerns	Suspected	No concerns	Major concerns	No concerns	Major concerns	Very low
iloperidone:olanzapine	0	Some concerns	Suspected	No concerns	Major concerns	No concerns	Major concerns	Very low
iloperidone:placebo	0	Some concerns	Suspected	No concerns	No concerns	Major concerns	Major concerns	Very low
iloperidone:quetiapine	0	Some concerns	Suspected	No concerns	Major concerns	No concerns	Major concerns	Very low
iloperidone:risp_pali	0	Some concerns	Suspected	No concerns	Major concerns	No concerns	Major concerns	Very low

iloperidone:sertindole	0	Some concerns	Suspected	No concerns	Major concerns	No concerns	Major concerns	Very low
iloperidone:ziprasidone	0	Some concerns	Suspected	No concerns	No concerns	No concerns	Major concerns	Very low
iloperidone:zotepine	0	Some concerns	Suspected	No concerns	Major concerns	No concerns	Major concerns	Very low
lurasidone:risp_pali	0	Some concerns	Suspected	No concerns	No concerns	Major concerns	Major concerns	Very low
lurasidone:sertindole	0	Some concerns	Suspected	No concerns	No concerns	No concerns	Major concerns	Low
lurasidone:zotepine	0	Some concerns	Suspected	No concerns	No concerns	No concerns	Major concerns	Low
olanzapine:sertindole	0	Some concerns	Suspected	No concerns	Major concerns	No concerns	Major concerns	Very low
olanzapine:zotepine	0	Some concerns	Suspected	No concerns	Major concerns	No concerns	Major concerns	Very low
placebo:sertindole	0	Some concerns	Suspected	No concerns	No concerns	No concerns	Major concerns	Low
placebo:zotepine	0	Some concerns	Suspected	No concerns	No concerns	No concerns	Major concerns	Low
quetiapine:sertindole	0	Some concerns	Suspected	No concerns	Major concerns	No concerns	Major concerns	Very low
quetiapine:ziprasidone	0	Some concerns	Suspected	No concerns	No concerns	No concerns	Major concerns	Low
quetiapine:zotepine	0	Some concerns	Suspected	No concerns	Major concerns	No concerns	Major concerns	Very low
risp_pali:ziprasidone	0	Some concerns	Suspected	No concerns	No concerns	No concerns	Major concerns	Low
risp_pali:zotepine	0	Some concerns	Suspected	No concerns	Major concerns	No concerns	Major concerns	Very low
sertindole:ziprasidone	0	Some concerns	Suspected	No concerns	No concerns	No concerns	Major concerns	Low
sertindole:zotepine	0	Some concerns	Suspected	No concerns	Major concerns	No concerns	Major concerns	Very low
ziprasidone:zotepine	0	Some concerns	Suspected	No concerns	No concerns	No concerns	Major concerns	Low

eTable 27. CINeMA confidence rating for BMI

Comparison	Number of studies	Within-study bias	Across-studies bias	Indirectness	Imprecision	Heterogeneity	Incoherence	Confidence rating
aripiprazole:placebo	1	Some concerns	Suspected	No concerns	Major concerns	No concerns	No concerns	Low
clozapine:haloperidol	1	Some concerns	Suspected	No concerns	No concerns	No concerns	No concerns	Moderate
clozapine:olanzapine	1	Some concerns	Suspected	No concerns	Major concerns	No concerns	No concerns	Low
clozapine:risp_pali	1	Some concerns	Suspected	No concerns	Major concerns	No concerns	No concerns	Low
haloperidol:olanzapine	3	Some concerns	Suspected	No concerns	No concerns	No concerns	No concerns	Moderate
haloperidol:risp_pali	2	Some concerns	Suspected	No concerns	No concerns	No concerns	No concerns	Moderate
lurasidone:olanzapine	1	Some concerns	Suspected	No concerns	No concerns	No concerns	No concerns	Moderate
lurasidone:placebo	4	Some concerns	Suspected	No concerns	No concerns	Major concerns	No concerns	Low
lurasidone:quetiapine	1	No concerns	Suspected	No concerns	No concerns	No concerns	No concerns	Moderate
olanzapine:placebo	3	Some concerns	Suspected	No concerns	No concerns	No concerns	No concerns	Moderate
olanzapine:risp_pali	9	Some concerns	Suspected	No concerns	No concerns	No concerns	No concerns	Moderate
placebo:quetiapine	1	No concerns	Suspected	No concerns	No concerns	No concerns	No concerns	Moderate
placebo:risp_pali	7	Some concerns	Suspected	No concerns	No concerns	No concerns	No concerns	Moderate
quetiapine:risp_pali	1	Some concerns	Suspected	No concerns	Major concerns	No concerns	No concerns	Low
risp_pali:sertindole	1	Some concerns	Suspected	No concerns	Major concerns	No concerns	No concerns	Low
aripiprazole:clozapine	0	Some concerns	Suspected	No concerns	No concerns	No concerns	No concerns	Moderate
aripiprazole:haloperidol	0	Some concerns	Suspected	No concerns	Major concerns	No concerns	No concerns	Very low
aripiprazole:lurasidone	0	Some concerns	Suspected	No concerns	Major concerns	No concerns	No concerns	Very low
aripiprazole:olanzapine	0	Some concerns	Suspected	No concerns	No concerns	No concerns	No concerns	Moderate
aripiprazole:quetiapine	0	Some concerns	Suspected	No concerns	No concerns	No concerns	No concerns	Moderate

aripiprazole:risp_pali	0	Some concerns	Suspected	No concerns	No concerns	No concerns	No concerns	Moderate
aripiprazole:sertindole	0	Some concerns	Suspected	No concerns	No concerns	No concerns	No concerns	Moderate
clozapine:lurasidone	0	Some concerns	Suspected	No concerns	No concerns	Major concerns	No concerns	Low
clozapine:placebo	0	Some concerns	Suspected	No concerns	No concerns	No concerns	No concerns	Moderate
clozapine:quetiapine	0	Some concerns	Suspected	No concerns	Major concerns	No concerns	No concerns	Low
clozapine:sertindole	0	Some concerns	Suspected	No concerns	Major concerns	No concerns	No concerns	Low
haloperidol:lurasidone	0	Some concerns	Suspected	No concerns	No concerns	Major concerns	No concerns	Low
haloperidol:placebo	0	Some concerns	Suspected	No concerns	Major concerns	No concerns	No concerns	Low
haloperidol:quetiapine	0	Some concerns	Suspected	No concerns	No concerns	No concerns	No concerns	Moderate
haloperidol:sertindole	0	Some concerns	Suspected	No concerns	No concerns	No concerns	No concerns	Moderate
lurasidone:risp_pali	0	Some concerns	Suspected	No concerns	No concerns	Major concerns	No concerns	Low
lurasidone:sertindole	0	Some concerns	Suspected	No concerns	Major concerns	No concerns	No concerns	Low
olanzapine:quetiapine	0	Some concerns	Suspected	No concerns	No concerns	Major concerns	No concerns	Low
olanzapine:sertindole	0	Some concerns	Suspected	No concerns	Major concerns	No concerns	No concerns	Low
placebo:sertindole	0	Some concerns	Suspected	No concerns	No concerns	No concerns	No concerns	Moderate
quetiapine:sertindole	0	Some concerns	Suspected	No concerns	Major concerns	No concerns	No concerns	Low

eTable 28. CINeMA confidence rating for total cholesterol

Comparison	Number of studies	Within-study bias	Across-studies bias	Indirectness	Imprecision	Heterogeneity	Incoherence	Confidence rating
amisulpride:olanzapine	1	No concerns	Suspected	No concerns	Major concerns	No concerns	No concerns	Low
aripiprazole:brexpiprazole	1	Some concerns	Suspected	No concerns	Major concerns	No concerns	No concerns	Low
aripiprazole:cariprazine	1	Some concerns	Suspected	No concerns	Major concerns	No concerns	No concerns	Low
aripiprazole:olanzapine	2	Some concerns	Suspected	No concerns	No concerns	No concerns	Major concerns	Low
aripiprazole:placebo	4	Some concerns	Suspected	No concerns	Major concerns	No concerns	No concerns	Low
aripiprazole:quetiapine	1	Some concerns	Suspected	No concerns	No concerns	No concerns	No concerns	Moderate
aripiprazole:risp_pali	3	Some concerns	Suspected	No concerns	Major concerns	No concerns	No concerns	Low
brexpiprazole:placebo	3	Some concerns	Suspected	No concerns	Major concerns	No concerns	No concerns	Low
cariprazine:placebo	1	Some concerns	Suspected	No concerns	Major concerns	No concerns	No concerns	Low
cariprazine:risp_pali	1	Some concerns	Suspected	No concerns	Major concerns	No concerns	No concerns	Low
clozapine:haloperidol	2	Some concerns	Suspected	No concerns	No concerns	No concerns	No concerns	Moderate
clozapine:olanzapine	2	Some concerns	Suspected	No concerns	Major concerns	No concerns	No concerns	Low
clozapine:risp_pali	1	Some concerns	Suspected	No concerns	No concerns	No concerns	No concerns	Moderate
haloperidol:iloperidone	1	Some concerns	Suspected	No concerns	No concerns	Major concerns	No concerns	Low
haloperidol:olanzapine	3	Some concerns	Suspected	No concerns	No concerns	No concerns	No concerns	Moderate
haloperidol:risp_pali	1	Some concerns	Suspected	No concerns	Major concerns	No concerns	No concerns	Low
lurasidone:olanzapine	1	Some concerns	Suspected	No concerns	No concerns	No concerns	No concerns	Moderate
lurasidone:placebo	3	No concerns	Suspected	No concerns	Major concerns	No concerns	No concerns	Low
lurasidone:ziprasidone	1	Some concerns	Suspected	No concerns	Major concerns	No concerns	No concerns	Low
olanzapine:placebo	5	Some concerns	Suspected	No concerns	No concerns	No concerns	No concerns	Moderate
olanzapine:quetiapine	1	Some concerns	Suspected	No concerns	Major concerns	No concerns	No concerns	Low
olanzapine:risp_pali	6	Some concerns	Suspected	No concerns	No concerns	No concerns	No concerns	Low
olanzapine:ziprasidone	3	Some concerns	Suspected	No concerns	No concerns	No concerns	No concerns	Low
placebo:quetiapine	4	Some concerns	Suspected	No concerns	No concerns	No concerns	No concerns	Moderate
placebo:risp_pali	6	Some concerns	Suspected	No concerns	Major concerns	No concerns	No concerns	Low
placebo:ziprasidone	1	Some concerns	Suspected	No concerns	Major concerns	No concerns	No concerns	Low
quetiapine:risp_pali	1	Some concerns	Suspected	No concerns	No concerns	No concerns	No concerns	Moderate
risp_pali:sertindole	1	Some concerns	Suspected	No concerns	Major concerns	No concerns	No concerns	Low
amisulpride:aripiprazole	0	Some concerns	Suspected	No concerns	Major concerns	No concerns	No concerns	Low
amisulpride:brexpiprazole	0	Some concerns	Suspected	No concerns	Major concerns	No concerns	No concerns	Low

amisulpride:cariprazine	0	Some concerns	Suspected	No concerns	Major concerns	No concerns	No concerns	Low
amisulpride:clozapine	0	Some concerns	Suspected	No concerns	Major concerns	No concerns	No concerns	Low
amisulpride:haloperidol	0	Some concerns	Suspected	No concerns	Major concerns	No concerns	No concerns	Low
amisulpride:iloperidone	0	Some concerns	Suspected	No concerns	Major concerns	No concerns	No concerns	Low
amisulpride:lurasidone	0	No concerns	Suspected	No concerns	Major concerns	No concerns	No concerns	Low
amisulpride:placebo	0	Some concerns	Suspected	No concerns	Major concerns	No concerns	No concerns	Low
amisulpride:quetiapine	0	Some concerns	Suspected	No concerns	Major concerns	No concerns	No concerns	Low
amisulpride:risp_pali	0	Some concerns	Suspected	No concerns	Major concerns	No concerns	No concerns	Low
amisulpride:sertindole	0	Some concerns	Suspected	No concerns	Major concerns	No concerns	No concerns	Low
amisulpride:ziprasidone	0	Some concerns	Suspected	No concerns	Major concerns	No concerns	No concerns	Low
aripiprazole:clozapine	0	Some concerns	Suspected	No concerns	No concerns	No concerns	No concerns	Moderate
aripiprazole:haloperidol	0	Some concerns	Suspected	No concerns	Major concerns	No concerns	No concerns	Low
aripiprazole:iloperidone	0	Some concerns	Suspected	No concerns	Major concerns	No concerns	No concerns	Low
aripiprazole:lurasidone	0	Some concerns	Suspected	No concerns	Major concerns	No concerns	No concerns	Low
aripiprazole:sertindole	0	Some concerns	Suspected	No concerns	Major concerns	No concerns	No concerns	Low
aripiprazole:ziprasidone	0	Some concerns	Suspected	No concerns	Major concerns	No concerns	No concerns	Low
brexpiprazole:cariprazine	0	Some concerns	Suspected	No concerns	Major concerns	No concerns	No concerns	Low
brexpiprazole:clozapine	0	Some concerns	Suspected	No concerns	No concerns	No concerns	No concerns	Moderate
brexpiprazole:haloperidol	0	Some concerns	Suspected	No concerns	Major concerns	No concerns	No concerns	Low
brexpiprazole:iloperidone	0	Some concerns	Suspected	No concerns	Major concerns	No concerns	No concerns	Low
brexpiprazole:lurasidone	0	Some concerns	Suspected	No concerns	Major concerns	No concerns	No concerns	Low
brexpiprazole:olanzapine	0	Some concerns	Suspected	No concerns	No concerns	No concerns	No concerns	Moderate
brexpiprazole:quetiapine	0	Some concerns	Suspected	No concerns	No concerns	No concerns	No concerns	Moderate
brexpiprazole:risp_pali	0	Some concerns	Suspected	No concerns	Major concerns	No concerns	No concerns	Low
brexpiprazole:sertindole	0	Some concerns	Suspected	No concerns	Major concerns	No concerns	No concerns	Low
brexpiprazole:ziprasidone	0	Some concerns	Suspected	No concerns	Major concerns	No concerns	No concerns	Low
cariprazine:clozapine	0	Some concerns	Suspected	No concerns	No concerns	No concerns	No concerns	Moderate
cariprazine:haloperidol	0	Some concerns	Suspected	No concerns	Major concerns	No concerns	No concerns	Low
cariprazine:iloperidone	0	Some concerns	Suspected	No concerns	Major concerns	No concerns	No concerns	Low
cariprazine:lurasidone	0	Some concerns	Suspected	No concerns	Major concerns	No concerns	No concerns	Low
cariprazine:olanzapine	0	Some concerns	Suspected	No concerns	No concerns	No concerns	No concerns	Moderate
cariprazine:quetiapine	0	Some concerns	Suspected	No concerns	No concerns	No concerns	No concerns	Moderate
cariprazine:sertindole	0	Some concerns	Suspected	No concerns	Major concerns	No concerns	No concerns	Low
cariprazine:ziprasidone	0	Some concerns	Suspected	No concerns	Major concerns	No concerns	No concerns	Low

clozapine:iloperidone	0	Some concerns	Suspected	No concerns	No concerns	No concerns	No concerns	Moderate
clozapine:lurasidone	0	Some concerns	Suspected	No concerns	No concerns	No concerns	No concerns	Moderate
clozapine:placebo	0	Some concerns	Suspected	No concerns	No concerns	No concerns	No concerns	Moderate
clozapine:quetiapine	0	Some concerns	Suspected	No concerns	Major concerns	No concerns	No concerns	Low
clozapine:sertindole	0	Some concerns	Suspected	No concerns	No concerns	No concerns	No concerns	Moderate
clozapine:ziprasidone	0	Some concerns	Suspected	No concerns	No concerns	No concerns	No concerns	Moderate
haloperidol:lurasidone	0	Some concerns	Suspected	No concerns	Major concerns	No concerns	No concerns	Low
haloperidol:placebo	0	Some concerns	Suspected	No concerns	Major concerns	No concerns	No concerns	Low
haloperidol:quetiapine	0	Some concerns	Suspected	No concerns	Major concerns	No concerns	No concerns	Low
haloperidol:sertindole	0	Some concerns	Suspected	No concerns	Major concerns	No concerns	No concerns	Low
haloperidol:ziprasidone	0	Some concerns	Suspected	No concerns	Major concerns	No concerns	No concerns	Low
iloperidone:lurasidone	0	Some concerns	Suspected	No concerns	Major concerns	No concerns	No concerns	Low
iloperidone:olanzapine	0	Some concerns	Suspected	No concerns	No concerns	No concerns	No concerns	Moderate
iloperidone:placebo	0	Some concerns	Suspected	No concerns	Major concerns	No concerns	No concerns	Low
iloperidone:quetiapine	0	Some concerns	Suspected	No concerns	No concerns	No concerns	No concerns	Moderate
iloperidone:risp_pali	0	Some concerns	Suspected	No concerns	Major concerns	No concerns	No concerns	Low
iloperidone:sertindole	0	Some concerns	Suspected	No concerns	Major concerns	No concerns	No concerns	Low
iloperidone:ziprasidone	0	Some concerns	Suspected	No concerns	Major concerns	No concerns	No concerns	Low
lurasidone:quetiapine	0	Some concerns	Suspected	No concerns	No concerns	No concerns	No concerns	Moderate
lurasidone:risp_pali	0	Some concerns	Suspected	No concerns	Major concerns	No concerns	No concerns	Low
lurasidone:sertindole	0	Some concerns	Suspected	No concerns	Major concerns	No concerns	No concerns	Low
olanzapine:sertindole	0	Some concerns	Suspected	No concerns	No concerns	No concerns	No concerns	Moderate
placebo:sertindole	0	Some concerns	Suspected	No concerns	Major concerns	No concerns	No concerns	Low
quetiapine:sertindole	0	Some concerns	Suspected	No concerns	No concerns	No concerns	No concerns	Moderate
quetiapine:ziprasidone	0	Some concerns	Suspected	No concerns	No concerns	No concerns	No concerns	Moderate
risp_pali:ziprasidone	0	Some concerns	Suspected	No concerns	Major concerns	No concerns	No concerns	Low
sertindole:ziprasidone	0	Some concerns	Suspected	No concerns	Major concerns	No concerns	No concerns	Low

eTable 29. CINeMA confidence rating for LDL Cholesterol

Comparison	Number of studies	Within-study bias	Across-studies bias	Indirectness	Imprecision	Heterogeneity	Incoherence	Confidence rating
aripiprazole:brexpiprazole	1	Some concerns	Suspected	No concerns	Major concerns	No concerns	No concerns	Low
aripiprazole:cariprazine	1	Some concerns	Suspected	No concerns	No concerns	No concerns	No concerns	Moderate
aripiprazole:olanzapine	2	Some concerns	Suspected	No concerns	No concerns	No concerns	No concerns	Moderate
aripiprazole:placebo	3	Some concerns	Suspected	No concerns	Major concerns	No concerns	No concerns	Low
aripiprazole:risp_pali	1	Some concerns	Suspected	No concerns	Major concerns	No concerns	No concerns	Low
brexpiprazole:olanzapine	1	Some concerns	Suspected	No concerns	No concerns	No concerns	No concerns	Moderate
brexpiprazole:placebo	2	Some concerns	Suspected	No concerns	Major concerns	No concerns	No concerns	Low
cariprazine:placebo	1	Some concerns	Suspected	No concerns	No concerns	No concerns	No concerns	Moderate
cariprazine:risp_pali	1	Some concerns	Suspected	No concerns	No concerns	No concerns	No concerns	Moderate
lurasidone:olanzapine	1	Some concerns	Suspected	No concerns	No concerns	No concerns	No concerns	Moderate
lurasidone:placebo	3	No concerns	Suspected	No concerns	Major concerns	No concerns	No concerns	Low
olanzapine:placebo	6	Some concerns	Suspected	No concerns	No concerns	No concerns	No concerns	Moderate
olanzapine:risp_pali	5	Some concerns	Suspected	No concerns	No concerns	No concerns	No concerns	Moderate
olanzapine:ziprasidone	2	Some concerns	Suspected	No concerns	No concerns	No concerns	No concerns	Moderate
placebo:quetiapine	3	Some concerns	Suspected	No concerns	No concerns	No concerns	No concerns	Moderate
placebo:risp_pali	6	Some concerns	Suspected	No concerns	Major concerns	No concerns	No concerns	Low
aripiprazole:lurasidone	0	Some concerns	Suspected	No concerns	Major concerns	No concerns	No concerns	Low
aripiprazole:quetiapine	0	Some concerns	Suspected	No concerns	No concerns	No concerns	No concerns	Moderate
aripiprazole:ziprasidone	0	Some concerns	Suspected	No concerns	Major concerns	No concerns	No concerns	Low
brexpiprazole:cariprazine	0	Some concerns	Suspected	No concerns	No concerns	No concerns	No concerns	Moderate
brexpiprazole:lurasidone	0	Some concerns	Suspected	No concerns	Major concerns	No concerns	No concerns	Low
brexpiprazole:quetiapine	0	Some concerns	Suspected	No concerns	No concerns	Major concerns	No concerns	Low
brexpiprazole:risp_pali	0	Some concerns	Suspected	No concerns	Major concerns	No concerns	No concerns	Low
brexpiprazole:ziprasidone	0	Some concerns	Suspected	No concerns	Major concerns	No concerns	No concerns	Low
cariprazine:lurasidone	0	Some concerns	Suspected	No concerns	Major concerns	No concerns	No concerns	Low
cariprazine:olanzapine	0	Some concerns	Suspected	No concerns	No concerns	No concerns	No concerns	Moderate
cariprazine:quetiapine	0	Some concerns	Suspected	No concerns	No concerns	No concerns	No concerns	Moderate
cariprazine:ziprasidone	0	Some concerns	Suspected	No concerns	Major concerns	No concerns	No concerns	Low
lurasidone:quetiapine	0	Some concerns	Suspected	No concerns	No concerns	No concerns	No concerns	Moderate
lurasidone:risp_pali	0	Some concerns	Suspected	No concerns	Major concerns	No concerns	No concerns	Low

lurasidone:ziprasidone	0	Some concerns	Suspected	No concerns	Major concerns	No concerns	No concerns	Low
olanzapine:quetiapine	0	Some concerns	Suspected	No concerns	Major concerns	No concerns	No concerns	Low
placebo:ziprasidone	0	Some concerns	Suspected	No concerns	Major concerns	No concerns	No concerns	Low
quetiapine:risp_pali	0	Some concerns	Suspected	No concerns	No concerns	No concerns	No concerns	Moderate
quetiapine:ziprasidone	0	Some concerns	Suspected	No concerns	No concerns	No concerns	No concerns	Moderate
risp_pali:ziprasidone	0	Some concerns	Suspected	No concerns	Major concerns	No concerns	No concerns	Low

eTable 30. CINeMA confidence rating for HDL Cholesterol

Comparison	Number of studies	Within-study bias	Across-studies bias	Indirectness	Imprecision	Heterogeneity	Incoherence	Confidence rating
amisulpride:olanzapine	1	No concerns	Suspected	No concerns	Major concerns	No concerns	No concerns	Low
aripiprazole:brexpiprazole	1	Some concerns	Suspected	No concerns	Major concerns	No concerns	No concerns	Low
aripiprazole:cariprazine	1	Some concerns	Suspected	No concerns	Major concerns	No concerns	No concerns	Low
aripiprazole:olanzapine	1	Some concerns	Suspected	No concerns	Major concerns	No concerns	Major concerns	Very low
aripiprazole:placebo	4	Some concerns	Suspected	No concerns	Major concerns	No concerns	Major concerns	Very low
aripiprazole:risp_pali	1	Some concerns	Suspected	No concerns	Major concerns	No concerns	No concerns	Low
brexpiprazole:placebo	2	Some concerns	Suspected	No concerns	No concerns	Major concerns	No concerns	Low
cariprazine:placebo	1	Some concerns	Suspected	No concerns	Major concerns	No concerns	No concerns	Low
cariprazine:risp_pali	1	Some concerns	Suspected	No concerns	Major concerns	No concerns	No concerns	Low
lurasidone:olanzapine	1	Some concerns	Suspected	No concerns	Major concerns	No concerns	No concerns	Low
lurasidone:placebo	1	Some concerns	Suspected	No concerns	Major concerns	No concerns	No concerns	Low
olanzapine:placebo	6	Some concerns	Suspected	No concerns	Major concerns	No concerns	No concerns	Low
olanzapine:risp_pali	4	Some concerns	Suspected	No concerns	Major concerns	No concerns	No concerns	Low
olanzapine:ziprasidone	2	Some concerns	Suspected	No concerns	Major concerns	No concerns	No concerns	Low
placebo:quetiapine	4	Some concerns	Suspected	No concerns	Major concerns	No concerns	No concerns	Low

placebo:risp_pali	6	Some concerns	Suspected	No concerns	Major concerns	No concerns	No concerns	Low
amisulpride:aripiprazole	0	Some concerns	Suspected	No concerns	Major concerns	No concerns	No concerns	Low
amisulpride:brexpiprazole	0	Some concerns	Suspected	No concerns	Major concerns	No concerns	No concerns	Low
amisulpride:cariprazine	0	Some concerns	Suspected	No concerns	Major concerns	No concerns	No concerns	Low
amisulpride:lurasidone	0	Some concerns	Suspected	No concerns	Major concerns	No concerns	No concerns	Low
amisulpride:placebo	0	No concerns	Suspected	No concerns	Major concerns	No concerns	No concerns	Low
amisulpride:quetiapine	0	Some concerns	Suspected	No concerns	Major concerns	No concerns	No concerns	Low
amisulpride:risp_pali	0	No concerns	Suspected	No concerns	Major concerns	No concerns	No concerns	Low
amisulpride:ziprasidone	0	Some concerns	Suspected	No concerns	Major concerns	No concerns	No concerns	Low
aripiprazole:lurasidone	0	Some concerns	Suspected	No concerns	Major concerns	No concerns	No concerns	Low
aripiprazole:quetiapine	0	Some concerns	Suspected	No concerns	Major concerns	No concerns	No concerns	Low
aripiprazole:ziprasidone	0	Some concerns	Suspected	No concerns	Major concerns	No concerns	No concerns	Low
brexpiprazole:cariprazine	0	Some concerns	Suspected	No concerns	Major concerns	No concerns	No concerns	Low
brexpiprazole:lurasidone	0	Some concerns	Suspected	No concerns	Major concerns	No concerns	No concerns	Low
brexpiprazole:olanzapine	0	Some concerns	Suspected	No concerns	Major concerns	No concerns	No concerns	Low
brexpiprazole:quetiapine	0	Some concerns	Suspected	No concerns	Major concerns	No concerns	No concerns	Low
brexpiprazole:risp_pali	0	Some concerns	Suspected	No concerns	Major concerns	No concerns	No concerns	Low
brexpiprazole:ziprasidone	0	Some concerns	Suspected	No concerns	Major concerns	No concerns	No concerns	Low
cariprazine:lurasidone	0	Some concerns	Suspected	No concerns	Major concerns	No concerns	No concerns	Low
cariprazine:olanzapine	0	Some concerns	Suspected	No concerns	Major concerns	No concerns	No concerns	Low
cariprazine:quetiapine	0	Some concerns	Suspected	No concerns	Major concerns	No concerns	No concerns	Low
cariprazine:ziprasidone	0	Some concerns	Suspected	No concerns	Major concerns	No concerns	No concerns	Low
lurasidone:quetiapine	0	Some concerns	Suspected	No concerns	Major concerns	No concerns	No concerns	Low

lurasidone:risp_pali	0	Some concerns	Suspected	No concerns	Major concerns	No concerns	No concerns	Low
lurasidone:ziprasidone	0	Some concerns	Suspected	No concerns	Major concerns	No concerns	No concerns	Low
olanzapine:quetiapine	0	Some concerns	Suspected	No concerns	Major concerns	No concerns	No concerns	Low
placebo:ziprasidone	0	Some concerns	Suspected	No concerns	Major concerns	No concerns	No concerns	Low
quetiapine:risp_pali	0	Some concerns	Suspected	No concerns	Major concerns	No concerns	No concerns	Low
quetiapine:ziprasidone	0	Some concerns	Suspected	No concerns	Major concerns	No concerns	No concerns	Low
risp_pali:ziprasidone	0	Some concerns	Suspected	No concerns	Major concerns	No concerns	No concerns	Low

eTable 31. CINeMA confidence rating for triglycerides

Comparison	Number of studies	Within-study bias	Across-studies bias	Indirectness	Imprecision	Heterogeneity	Incoherence	Confidence rating
amisulpride:olanzapine	1	No concerns	Suspected	No concerns	Major concerns	No concerns	Major concerns	Very low
aripiprazole:brexpiprazole	1	Some concerns	Suspected	No concerns	Major concerns	No concerns	No concerns	Low
aripiprazole:cariprazine	1	Some concerns	Suspected	No concerns	Major concerns	No concerns	No concerns	Low
aripiprazole:olanzapine	2	Some concerns	Suspected	No concerns	No concerns	No concerns	No concerns	Moderate
aripiprazole:placebo	4	Some concerns	Suspected	No concerns	Major concerns	No concerns	No concerns	Low
aripiprazole:quetiapine	1	Some concerns	Suspected	No concerns	No concerns	No concerns	No concerns	Moderate
aripiprazole:risp_pali	2	Some concerns	Suspected	No concerns	Major concerns	No concerns	No concerns	Low
brexpiprazole:placebo	3	Some concerns	Suspected	No concerns	Major concerns	No concerns	No concerns	Low
cariprazine:placebo	1	Some concerns	Suspected	No concerns	Major concerns	No concerns	No concerns	Low
cariprazine:risp_pali	1	Some concerns	Suspected	No concerns	Major concerns	No concerns	No concerns	Low
clozapine:haloperidol	1	Some concerns	Suspected	No concerns	No concerns	No concerns	Major concerns	Moderate
clozapine:olanzapine	1	Some concerns	Suspected	No concerns	No concerns	Major concerns	Major concerns	Very low
clozapine:zotepine	1	Some concerns	Suspected	No concerns	Major concerns	No concerns	Major concerns	Very low

haloperidol:iloperidone	1	Some concerns	Suspected	No concerns	Major concerns	No concerns	Major concerns	Very low
haloperidol:olanzapine	1	Some concerns	Suspected	No concerns	Major concerns	No concerns	Major concerns	Very low
lurasidone:olanzapine	1	Some concerns	Suspected	No concerns	No concerns	No concerns	No concerns	Moderate
lurasidone:placebo	3	No concerns	Suspected	No concerns	Major concerns	No concerns	No concerns	Low
lurasidone:ziprasidone	1	Some concerns	Suspected	No concerns	Major concerns	No concerns	No concerns	Low
olanzapine:placebo	5	Some concerns	Suspected	No concerns	No concerns	No concerns	No concerns	Moderate
olanzapine:quetiapine	1	Some concerns	Suspected	No concerns	Major concerns	No concerns	No concerns	Low
olanzapine:risp_pali	5	Some concerns	Suspected	No concerns	No concerns	No concerns	No concerns	Moderate
olanzapine:ziprasidone	3	Some concerns	Suspected	No concerns	No concerns	No concerns	No concerns	Moderate
placebo:quetiapine	4	Some concerns	Suspected	No concerns	No concerns	No concerns	No concerns	Moderate
placebo:risp_pali	6	Some concerns	Suspected	No concerns	Major concerns	No concerns	No concerns	Low
placebo:ziprasidone	1	Major concerns	Suspected	No concerns	Major concerns	No concerns	No concerns	Very low
quetiapine:risp_pali	1	Some concerns	Suspected	No concerns	No concerns	No concerns	Major concerns	Low
risp_pali:sertindole	1	Some concerns	Suspected	No concerns	Major concerns	No concerns	Major concerns	Very low
amisulpride:aripiprazole	0	Some concerns	Suspected	No concerns	Major concerns	No concerns	Major concerns	Very low
amisulpride:brexpiprazole	0	Some concerns	Suspected	No concerns	Major concerns	No concerns	Major concerns	Very low
amisulpride:cariprazine	0	Some concerns	Suspected	No concerns	Major concerns	No concerns	Major concerns	Very low
amisulpride:clozapine	0	Some concerns	Suspected	No concerns	No concerns	No concerns	Major concerns	Low
amisulpride:haloperidol	0	Some concerns	Suspected	No concerns	Major concerns	No concerns	Major concerns	Very low
amisulpride:iloperidone	0	Some concerns	Suspected	No concerns	Major concerns	No concerns	Major concerns	Very low
amisulpride:lurasidone	0	No concerns	Suspected	No concerns	Major concerns	No concerns	Major concerns	Very low
amisulpride:placebo	0	Some concerns	Suspected	No concerns	Major concerns	No concerns	Major concerns	Very low
amisulpride:quetiapine	0	Some concerns	Suspected	No concerns	Major concerns	No concerns	Major concerns	Very low

amisulpride:risp_pali	0	Some concerns	Suspected	No concerns	Major concerns	No concerns	Major concerns	Very low
amisulpride:sertindole	0	Some concerns	Suspected	No concerns	Major concerns	No concerns	Major concerns	Very low
amisulpride:ziprasidone	0	Some concerns	Suspected	No concerns	Major concerns	No concerns	Major concerns	Very low
amisulpride:zotepine	0	Some concerns	Suspected	No concerns	Major concerns	No concerns	Major concerns	Very low
aripiprazole:clozapine	0	Some concerns	Suspected	No concerns	No concerns	No concerns	Major concerns	Low
aripiprazole:haloperidol	0	Some concerns	Suspected	No concerns	Major concerns	No concerns	Major concerns	Very low
aripiprazole:iloperidone	0	Some concerns	Suspected	No concerns	Major concerns	No concerns	Major concerns	Very low
aripiprazole:lurasidone	0	Some concerns	Suspected	No concerns	Major concerns	No concerns	Major concerns	Very low
aripiprazole:sertindole	0	Some concerns	Suspected	No concerns	Major concerns	No concerns	Major concerns	Very low
aripiprazole:ziprasidone	0	Some concerns	Suspected	No concerns	Major concerns	No concerns	Major concerns	Very low
aripiprazole:zotepine	0	Some concerns	Suspected	No concerns	No concerns	No concerns	Major concerns	Low
brexpiprazole:cariprazine	0	Some concerns	Suspected	No concerns	Major concerns	No concerns	Major concerns	Very low
brexpiprazole:clozapine	0	Some concerns	Suspected	No concerns	No concerns	No concerns	Major concerns	Low
brexpiprazole:haloperidol	0	Some concerns	Suspected	No concerns	Major concerns	No concerns	Major concerns	Very low
brexpiprazole:iloperidone	0	Some concerns	Suspected	No concerns	Major concerns	No concerns	Major concerns	Very low
brexpiprazole:lurasidone	0	Some concerns	Suspected	No concerns	Major concerns	No concerns	Major concerns	Very low
brexpiprazole:olanzapine	0	Some concerns	Suspected	No concerns	No concerns	No concerns	Major concerns	Low
brexpiprazole:quetiapine	0	Some concerns	Suspected	No concerns	No concerns	No concerns	Major concerns	Low
brexpiprazole:risp_pali	0	Some concerns	Suspected	No concerns	Major concerns	No concerns	Major concerns	Very low
brexpiprazole:sertindole	0	Some concerns	Suspected	No concerns	Major concerns	No concerns	Major concerns	Very low
brexpiprazole:ziprasidone	0	Some concerns	Suspected	No concerns	Major concerns	No concerns	Major concerns	Very low
brexpiprazole:zotepine	0	Some concerns	Suspected	No concerns	No concerns	No concerns	Major concerns	Low
cariprazine:clozapine	0	Some concerns	Suspected	No concerns	No concerns	No concerns	Major concerns	Low

cariprazine:haloperidol	0	Some concerns	Suspected	No concerns	Major concerns	No concerns	Major concerns	Very low
cariprazine:iloperidone	0	Some concerns	Suspected	No concerns	Major concerns	No concerns	Major concerns	Very low
cariprazine:lurasidone	0	Some concerns	Suspected	No concerns	Major concerns	No concerns	Major concerns	Very low
cariprazine:olanzapine	0	Some concerns	Suspected	No concerns	No concerns	No concerns	Major concerns	Low
cariprazine:quetiapine	0	Some concerns	Suspected	No concerns	No concerns	No concerns	Major concerns	Low
cariprazine:sertindole	0	Some concerns	Suspected	No concerns	Major concerns	No concerns	Major concerns	Very low
cariprazine:ziprasidone	0	Some concerns	Suspected	No concerns	Major concerns	No concerns	Major concerns	Very low
cariprazine:zotepine	0	Some concerns	Suspected	No concerns	No concerns	No concerns	Major concerns	Low
clozapine:iloperidone	0	Some concerns	Suspected	No concerns	No concerns	No concerns	Major concerns	Low
clozapine:lurasidone	0	Some concerns	Suspected	No concerns	No concerns	No concerns	Major concerns	Low
clozapine:placebo	0	Some concerns	Suspected	No concerns	No concerns	No concerns	Major concerns	Low
clozapine:quetiapine	0	Some concerns	Suspected	No concerns	No concerns	No concerns	Major concerns	Low
clozapine:risp_pali	0	Some concerns	Suspected	No concerns	No concerns	No concerns	Major concerns	Low
clozapine:sertindole	0	Some concerns	Suspected	No concerns	No concerns	No concerns	Major concerns	Low
clozapine:ziprasidone	0	Some concerns	Suspected	No concerns	No concerns	No concerns	Major concerns	Low
haloperidol:lurasidone	0	Some concerns	Suspected	No concerns	Major concerns	No concerns	Major concerns	Very low
haloperidol:placebo	0	Some concerns	Suspected	No concerns	Major concerns	No concerns	Major concerns	Very low
haloperidol:quetiapine	0	Some concerns	Suspected	No concerns	Major concerns	No concerns	Major concerns	Very low
haloperidol:risp_pali	0	Some concerns	Suspected	No concerns	Major concerns	No concerns	Major concerns	Very low
haloperidol:sertindole	0	Some concerns	Suspected	No concerns	Major concerns	No concerns	Major concerns	Very low
haloperidol:ziprasidone	0	Some concerns	Suspected	No concerns	Major concerns	No concerns	Major concerns	Very low
haloperidol:zotepine	0	Some concerns	Suspected	No concerns	Major concerns	No concerns	Major concerns	Very low
iloperidone:lurasidone	0	Some concerns	Suspected	No concerns	Major concerns	No concerns	Major concerns	Very low

iloperidone:olanzapine	0	Some concerns	Suspected	No concerns	Major concerns	No concerns	Major concerns	Very low
iloperidone:placebo	0	Some concerns	Suspected	No concerns	Major concerns	No concerns	Major concerns	Very low
iloperidone:quetiapine	0	Some concerns	Suspected	No concerns	Major concerns	No concerns	Major concerns	Very low
iloperidone:risp_pali	0	Some concerns	Suspected	No concerns	Major concerns	No concerns	Major concerns	Very low
iloperidone:sertindole	0	Some concerns	Suspected	No concerns	Major concerns	No concerns	Major concerns	Very low
iloperidone:ziprasidone	0	Some concerns	Suspected	No concerns	Major concerns	No concerns	Major concerns	Very low
iloperidone:zotepine	0	Some concerns	Suspected	No concerns	Major concerns	No concerns	Major concerns	Very low
lurasidone:quetiapine	0	Some concerns	Suspected	No concerns	No concerns	No concerns	Major concerns	Low
lurasidone:risp_pali	0	Some concerns	Suspected	No concerns	Major concerns	No concerns	Major concerns	Very low
lurasidone:sertindole	0	Some concerns	Suspected	No concerns	Major concerns	No concerns	Major concerns	Very low
lurasidone:zotepine	0	Some concerns	Suspected	No concerns	No concerns	No concerns	Major concerns	Low
olanzapine:sertindole	0	Some concerns	Suspected	No concerns	No concerns	No concerns	Major concerns	Low
olanzapine:zotepine	0	Some concerns	Suspected	No concerns	Major concerns	No concerns	Major concerns	Very low
placebo:sertindole	0	Some concerns	Suspected	No concerns	Major concerns	No concerns	Major concerns	Very low
placebo:zotepine	0	Some concerns	Suspected	No concerns	No concerns	No concerns	Major concerns	Low
quetiapine:sertindole	0	Some concerns	Suspected	No concerns	Major concerns	No concerns	Major concerns	Very low
quetiapine:ziprasidone	0	Some concerns	Suspected	No concerns	No concerns	No concerns	Major concerns	Low
quetiapine:zotepine	0	Some concerns	Suspected	No concerns	Major concerns	No concerns	Major concerns	Very low
risp_pali:ziprasidone	0	Some concerns	Suspected	No concerns	Major concerns	No concerns	Major concerns	Very low
risp_pali:zotepine	0	Some concerns	Suspected	No concerns	No concerns	No concerns	Major concerns	Low
sertindole:ziprasidone	0	Some concerns	Suspected	No concerns	Major concerns	No concerns	Major concerns	Very low
sertindole:zotepine	0	Some concerns	Suspected	No concerns	No concerns	No concerns	Major concerns	Low
ziprasidone:zotepine	0	Some concerns	Suspected	No concerns	No concerns	No concerns	Major concerns	Low

eTable 32. CINeMA confidence rating for glucose

Comparison	Number of studies	Within-study bias	Across-studies bias	Indirectness	Imprecision	Heterogeneity	Incoherence	Confidence rating
amisulpride:olanzapine	1	No concerns	Suspected	No concerns	Major concerns	No concerns	No concerns	Low
aripiprazole:brexpiprazole	1	Major concerns	Suspected	No concerns	Major concerns	No concerns	No concerns	Very low
aripiprazole:cariprazine	1	Some concerns	Suspected	No concerns	Major concerns	No concerns	No concerns	Low
aripiprazole:olanzapine	2	Some concerns	Suspected	No concerns	Major concerns	No concerns	No concerns	Low
aripiprazole:placebo	4	Some concerns	Suspected	No concerns	Major concerns	No concerns	No concerns	Low
aripiprazole:quetiapine	1	Some concerns	Suspected	No concerns	Major concerns	No concerns	No concerns	Low
aripiprazole:risp_pali	3	Some concerns	Suspected	No concerns	Major concerns	No concerns	No concerns	Low
asenapine:placebo	1	No concerns	Suspected	No concerns	Major concerns	No concerns	No concerns	Low
brexpiprazole:placebo	3	Major concerns	Suspected	No concerns	Major concerns	No concerns	No concerns	Very low
cariprazine:placebo	1	Some concerns	Suspected	No concerns	Major concerns	No concerns	Major concerns	Very low
cariprazine:risp_pali	1	Some concerns	Suspected	No concerns	Major concerns	No concerns	No concerns	Low
clozapine:haloperidol	2	Some concerns	Suspected	No concerns	No concerns	No concerns	No concerns	Moderate
clozapine:olanzapine	2	Some concerns	Suspected	No concerns	No concerns	No concerns	No concerns	Moderate
clozapine:risp_pali	1	Some concerns	Suspected	No concerns	No concerns	No concerns	No concerns	Moderate
clozapine:zotepine	1	Some concerns	Suspected	No concerns	Major concerns	No concerns	No concerns	Low
haloperidol:iloperidone	1	Some concerns	Suspected	No concerns	Major concerns	No concerns	No concerns	Low
haloperidol:olanzapine	3	Some concerns	Suspected	No concerns	Major concerns	No concerns	No concerns	Low
haloperidol:risp_pali	1	Some concerns	Suspected	No concerns	Major concerns	No concerns	No concerns	Low
lurasidone:olanzapine	1	Some concerns	Suspected	No concerns	No concerns	No concerns	No concerns	Moderate
lurasidone:placebo	3	No concerns	Suspected	No concerns	No concerns	Major concerns	No concerns	Low

olanzapine:placebo	6	Some concerns	Suspected	No concerns	No concerns	Major concerns	No concerns	Low
olanzapine:quetiapine	1	Some concerns	Suspected	No concerns	Major concerns	No concerns	No concerns	Low
olanzapine:risp_pali	6	Some concerns	Suspected	No concerns	Major concerns	No concerns	No concerns	Low
olanzapine:ziprasidone	2	Some concerns	Suspected	No concerns	Major concerns	No concerns	No concerns	Low
placebo:quetiapine	4	Some concerns	Suspected	No concerns	Major concerns	No concerns	No concerns	Low
placebo:risp_pali	9	Some concerns	Suspected	No concerns	Major concerns	No concerns	No concerns	Low
quetiapine:risp_pali	1	Some concerns	Suspected	No concerns	Major concerns	No concerns	No concerns	Low
risp_pali:sertindole	1	Some concerns	Suspected	No concerns	Major concerns	No concerns	No concerns	Low
amisulpride:aripiprazole	0	Some concerns	Suspected	No concerns	Major concerns	No concerns	No concerns	Low
amisulpride:asenapine	0	No concerns	Suspected	No concerns	Major concerns	No concerns	No concerns	Low
amisulpride:brexpiprazole	0	Some concerns	Suspected	No concerns	Major concerns	No concerns	No concerns	Low
amisulpride:cariprazine	0	Some concerns	Suspected	No concerns	Major concerns	No concerns	No concerns	Low
amisulpride:clozapine	0	Some concerns	Suspected	No concerns	No concerns	No concerns	No concerns	Moderate
amisulpride:haloperidol	0	No concerns	Suspected	No concerns	Major concerns	No concerns	No concerns	Low
amisulpride:iloperidone	0	Some concerns	Suspected	No concerns	Major concerns	No concerns	No concerns	Low
amisulpride:lurasidone	0	No concerns	Suspected	No concerns	Major concerns	No concerns	No concerns	Low
amisulpride:placebo	0	Some concerns	Suspected	No concerns	Major concerns	No concerns	No concerns	Low
amisulpride:quetiapine	0	Some concerns	Suspected	No concerns	Major concerns	No concerns	No concerns	Low
amisulpride:risp_pali	0	Some concerns	Suspected	No concerns	Major concerns	No concerns	No concerns	Low
amisulpride:sertindole	0	Some concerns	Suspected	No concerns	Major concerns	No concerns	No concerns	Low
amisulpride:ziprasidone	0	Some concerns	Suspected	No concerns	Major concerns	No concerns	No concerns	Low
amisulpride:zotepine	0	Some concerns	Suspected	No concerns	No concerns	No concerns	No concerns	Moderate
aripiprazole:asenapine	0	Some concerns	Suspected	No concerns	Major concerns	No concerns	No concerns	Low

aripiprazole:clozapine	0	Some concerns	Suspected	No concerns	No concerns	No concerns	No concerns	Moderate
aripiprazole:haloperidol	0	Some concerns	Suspected	No concerns	Major concerns	No concerns	No concerns	Low
aripiprazole:iloperidone	0	Some concerns	Suspected	No concerns	Major concerns	No concerns	No concerns	Low
aripiprazole:lurasidone	0	Some concerns	Suspected	No concerns	No concerns	Major concerns	No concerns	Low
aripiprazole:sertindole	0	Some concerns	Suspected	No concerns	Major concerns	No concerns	No concerns	Low
aripiprazole:ziprasidone	0	Some concerns	Suspected	No concerns	Major concerns	No concerns	No concerns	Low
aripiprazole:zotepine	0	Some concerns	Suspected	No concerns	No concerns	Major concerns	No concerns	Low
asenapine:brexpiprazole	0	Some concerns	Suspected	No concerns	Major concerns	No concerns	No concerns	Low
asenapine:cariprazine	0	Some concerns	Suspected	No concerns	Major concerns	No concerns	No concerns	Low
asenapine:clozapine	0	Some concerns	Suspected	No concerns	No concerns	No concerns	No concerns	Moderate
asenapine:haloperidol	0	Some concerns	Suspected	No concerns	Major concerns	No concerns	No concerns	Low
asenapine:iloperidone	0	Some concerns	Suspected	No concerns	Major concerns	No concerns	No concerns	Low
asenapine:lurasidone	0	No concerns	Suspected	No concerns	Major concerns	No concerns	No concerns	Low
asenapine:olanzapine	0	Some concerns	Suspected	No concerns	Major concerns	No concerns	No concerns	Low
asenapine:quetiapine	0	Some concerns	Suspected	No concerns	Major concerns	No concerns	No concerns	Low
asenapine:risp_pali	0	Some concerns	Suspected	No concerns	Major concerns	No concerns	No concerns	Low
asenapine:sertindole	0	Some concerns	Suspected	No concerns	Major concerns	No concerns	No concerns	Low
asenapine:ziprasidone	0	Some concerns	Suspected	No concerns	Major concerns	No concerns	No concerns	Low
asenapine:zotepine	0	Some concerns	Suspected	No concerns	No concerns	No concerns	No concerns	Moderate
brexpiprazole:cariprazine	0	Some concerns	Suspected	No concerns	Major concerns	No concerns	No concerns	Low
brexpiprazole:clozapine	0	Some concerns	Suspected	No concerns	No concerns	No concerns	No concerns	Moderate
brexpiprazole:haloperidol	0	Some concerns	Suspected	No concerns	Major concerns	No concerns	No concerns	Low
brexpiprazole:iloperidone	0	Some concerns	Suspected	No concerns	Major concerns	No concerns	No concerns	Low

brexpiprazole:lurasidone	0	Some concerns	Suspected	No concerns	Major concerns	No concerns	No concerns	Low
brexpiprazole:olanzapine	0	Some concerns	Suspected	No concerns	Major concerns	No concerns	No concerns	Low
brexpiprazole:quetiapine	0	Some concerns	Suspected	No concerns	Major concerns	No concerns	No concerns	Low
brexpiprazole:risp_pali	0	Some concerns	Suspected	No concerns	Major concerns	No concerns	No concerns	Low
brexpiprazole:sertindole	0	Some concerns	Suspected	No concerns	Major concerns	No concerns	No concerns	Low
brexpiprazole:ziprasidone	0	Some concerns	Suspected	No concerns	Major concerns	No concerns	No concerns	Low
brexpiprazole:zotepine	0	Some concerns	Suspected	No concerns	No concerns	Major concerns	No concerns	Low
cariprazine:clozapine	0	Some concerns	Suspected	No concerns	No concerns	Major concerns	No concerns	Low
cariprazine:haloperidol	0	Some concerns	Suspected	No concerns	Major concerns	No concerns	No concerns	Low
cariprazine:iloperidone	0	Some concerns	Suspected	No concerns	Major concerns	No concerns	No concerns	Low
cariprazine:lurasidone	0	Some concerns	Suspected	No concerns	No concerns	Major concerns	No concerns	Low
cariprazine:olanzapine	0	Some concerns	Suspected	No concerns	Major concerns	No concerns	No concerns	Low
cariprazine:quetiapine	0	Some concerns	Suspected	No concerns	Major concerns	No concerns	No concerns	Low
cariprazine:sertindole	0	Some concerns	Suspected	No concerns	Major concerns	No concerns	No concerns	Low
cariprazine:ziprasidone	0	Some concerns	Suspected	No concerns	Major concerns	No concerns	No concerns	Low
cariprazine:zotepine	0	Some concerns	Suspected	No concerns	Major concerns	No concerns	No concerns	Low
clozapine:iloperidone	0	Some concerns	Suspected	No concerns	Major concerns	No concerns	No concerns	Low
clozapine:lurasidone	0	Some concerns	Suspected	No concerns	No concerns	No concerns	No concerns	Moderate
clozapine:placebo	0	Some concerns	Suspected	No concerns	No concerns	No concerns	No concerns	Moderate
clozapine:quetiapine	0	Some concerns	Suspected	No concerns	No concerns	No concerns	No concerns	Moderate
clozapine:sertindole	0	Some concerns	Suspected	No concerns	No concerns	No concerns	No concerns	Moderate
clozapine:ziprasidone	0	Some concerns	Suspected	No concerns	No concerns	No concerns	No concerns	Moderate
haloperidol:lurasidone	0	Some concerns	Suspected	No concerns	No concerns	Major concerns	No concerns	Low

haloperidol:placebo	0	Some concerns	Suspected	No concerns	Major concerns	No concerns	No concerns	Low
haloperidol:quetiapine	0	Some concerns	Suspected	No concerns	Major concerns	No concerns	No concerns	Low
haloperidol:sertindole	0	Some concerns	Suspected	No concerns	Major concerns	No concerns	No concerns	Low
haloperidol:ziprasidone	0	Some concerns	Suspected	No concerns	Major concerns	No concerns	No concerns	Low
haloperidol:zotepine	0	Some concerns	Suspected	No concerns	Major concerns	No concerns	No concerns	Low
iloperidone:lurasidone	0	Some concerns	Suspected	No concerns	No concerns	Major concerns	No concerns	Low
iloperidone:olanzapine	0	Some concerns	Suspected	No concerns	Major concerns	No concerns	No concerns	Low
iloperidone:placebo	0	Some concerns	Suspected	No concerns	Major concerns	No concerns	No concerns	Low
iloperidone:quetiapine	0	Some concerns	Suspected	No concerns	Major concerns	No concerns	No concerns	Low
iloperidone:risp_pali	0	Some concerns	Suspected	No concerns	Major concerns	No concerns	No concerns	Low
iloperidone:sertindole	0	Some concerns	Suspected	No concerns	Major concerns	No concerns	No concerns	Low
iloperidone:ziprasidone	0	Some concerns	Suspected	No concerns	Major concerns	No concerns	No concerns	Low
iloperidone:zotepine	0	Some concerns	Suspected	No concerns	Major concerns	No concerns	No concerns	Low
lurasidone:quetiapine	0	Some concerns	Suspected	No concerns	No concerns	Major concerns	No concerns	Low
lurasidone:risp_pali	0	Some concerns	Suspected	No concerns	No concerns	Major concerns	No concerns	Low
lurasidone:sertindole	0	Some concerns	Suspected	No concerns	Major concerns	No concerns	No concerns	Low
lurasidone:ziprasidone	0	Some concerns	Suspected	No concerns	Major concerns	No concerns	No concerns	Low
lurasidone:zotepine	0	Some concerns	Suspected	No concerns	No concerns	No concerns	No concerns	Moderate
olanzapine:sertindole	0	Some concerns	Suspected	No concerns	Major concerns	No concerns	No concerns	Low
olanzapine:zotepine	0	Some concerns	Suspected	No concerns	Major concerns	No concerns	No concerns	Low
placebo:sertindole	0	Some concerns	Suspected	No concerns	Major concerns	No concerns	No concerns	Low
placebo:ziprasidone	0	Some concerns	Suspected	No concerns	Major concerns	No concerns	No concerns	Low
placebo:zotepine	0	Some concerns	Suspected	No concerns	No concerns	No concerns	No concerns	Moderate

quetiapine:sertindole	0	Some concerns	Suspected	No concerns	Major concerns	No concerns	No concerns	Low
quetiapine:ziprasidone	0	Some concerns	Suspected	No concerns	Major concerns	No concerns	No concerns	Low
quetiapine:zotepine	0	Some concerns	Suspected	No concerns	No concerns	Major concerns	No concerns	Low
risp_pali:ziprasidone	0	Some concerns	Suspected	No concerns	Major concerns	No concerns	No concerns	Low
risp_pali:zotepine	0	Some concerns	Suspected	No concerns	No concerns	Major concerns	No concerns	Low
sertindole:ziprasidone	0	Some concerns	Suspected	No concerns	Major concerns	No concerns	No concerns	Low
sertindole:zotepine	0	Some concerns	Suspected	No concerns	No concerns	Major concerns	No concerns	Low
ziprasidone:zotepine	0	Some concerns	Suspected	No concerns	No concerns	Major concerns	No concerns	Low

eAppendix 4. Post hoc sensitivity analysis for antipsychotic-induced weight gain (low quality studies removed)

In the context of increased heterogeneity and inconsistency in the NMA examining antipsychotic-induced weight change, we performed a post-hoc sensitivity analysis excluding studies at high risk of bias (identified in eTable 3). Results were broadly similar.

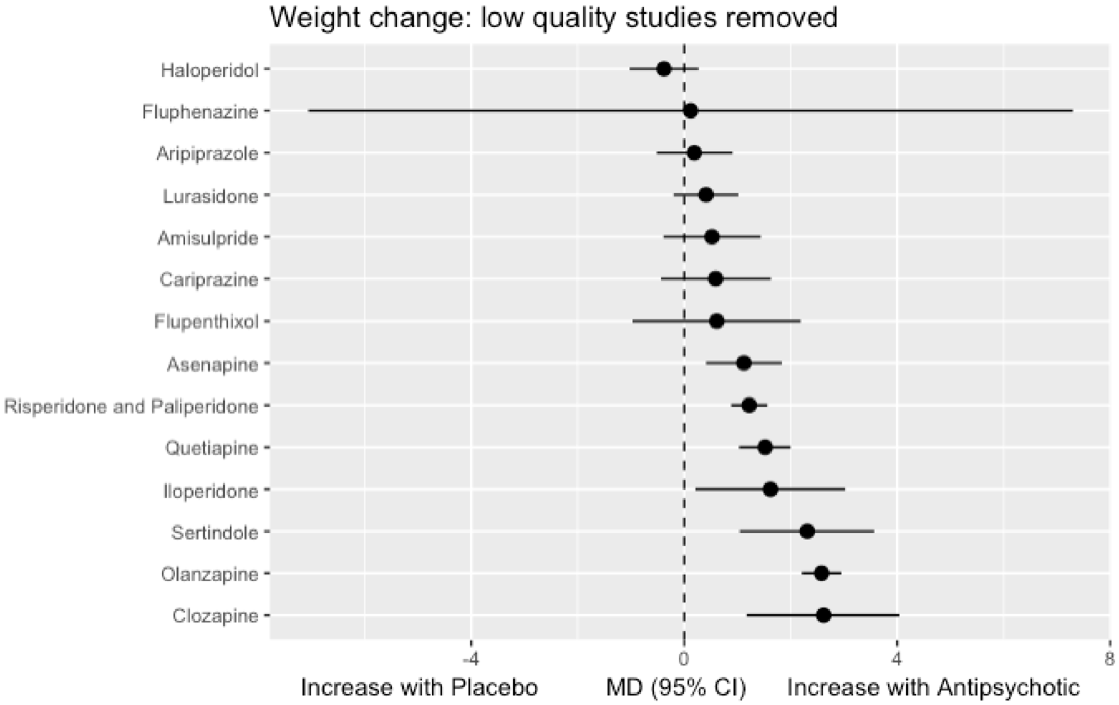

Tests of heterogeneity and inconsistency were as follows: τ was 0.61kg, and I^2 72%. The global Q score for inconsistency was 75.99 (p=0.0002), and significant hot-spots of inconsistency identified in 6 of 154 treatment comparisons including disagreement between direct and indirect evidence (see below).

```
Random effects model
k          - Number of studies providing direct evidence
prop       - Direct evidence proportion
NMA        - Estimated treatment effect (MD) in network meta-analysis
direct     - Estimated treatment effect (MD) derived from direct evidence
indir.     - Estimated treatment effect (MD) derived from indirect evidence
Diff       - Difference between direct and indirect treatment estimates
z          - z-value of test for disagreement (direct versus indirect)
p-value    - p-value of test for disagreement (direct versus indirect)
```

comparison	k	prop	nma	direct	indir.	Diff	z	p-value
amisulpride:aripiprazole	0	0	0.3305	.	0.3305	.	.	.
amisulpride:asenapine	0	0	-0.5931	.	-0.5931	.	.	.
amisulpride:cariprazine	0	0	-0.0685	.	-0.0685	.	.	.
amisulpride:clozapine	0	0	-2.1000	.	-2.1000	.	.	.
amisulpride:flupenthixol	1	0.60	-0.0842	-1.2000	1.5740	-2.7740	-1.76	0.0777
amisulpride:fluphenazine	0	0	0.4073	.	0.4073	.	.	.
amisulpride:haloperidol	0	0	0.9055	.	0.9055	.	.	.
amisulpride:iloperidone	0	0	-1.0945	.	-1.0945	.	.	.
amisulpride:lurasidone	0	0	0.1098	.	0.1098	.	.	.
amisulpride:olanzapine	2	0.76	-2.0562	-1.6096	-3.4801	1.8705	1.84	0.0660
amisulpride:placebo	0	0	0.5238	.	0.5238	.	.	.
amisulpride:quetiapine	0	0	-0.9927	.	-0.9927	.	.	.
amisulpride:risp_pali	1	0.18	-0.6961	-1.5000	-0.5201	-0.9799	-0.82	0.4127
amisulpride:sertindole	0	0	-1.7840	.	-1.7840	.	.	.
amisulpride:ziprasidone	0	0	2.1581	.	2.1581	.	.	.
amisulpride:zotepine	0	0	-2.0157	.	-2.0157	.	.	.
aripiprazole:asenapine	0	0	-0.9236	.	-0.9236	.	.	.
aripiprazole:cariprazine	1	0.75	-0.3990	-0.3723	-0.4804	0.1081	0.08	0.9341
aripiprazole:clozapine	0	0	-2.4304	.	-2.4304	.	.	.
aripiprazole:flupenthixol	0	0	-0.4147	.	-0.4147	.	.	.
aripiprazole:fluphenazine	0	0	0.0768	.	0.0768	.	.	.
aripiprazole:haloperidol	0	0	0.5750	.	0.5750	.	.	.
aripiprazole:iloperidone	0	0	-1.4250	.	-1.4250	.	.	.
aripiprazole:lurasidone	0	0	-0.2207	.	-0.2207	.	.	.
aripiprazole:olanzapine	2	0.24	-2.3867	-2.6352	-2.3073	-0.3279	-0.37	0.7091
aripiprazole:placebo	2	0.43	0.1934	0.1659	0.2144	-0.0486	-0.07	0.9476
aripiprazole:quetiapine	1	0.14	-1.3232	-1.7000	-1.2618	-0.4382	-0.36	0.7164
aripiprazole:risp_pali	4	0.64	-1.0266	-0.7352	-1.5441	0.8089	1.08	0.2804
aripiprazole:sertindole	0	0	-2.1145	.	-2.1145	.	.	.
aripiprazole:ziprasidone	0	0	1.8277	.	1.8277	.	.	.
aripiprazole:zotepine	0	0	-2.3461	.	-2.3461	.	.	.
asenapine:cariprazine	0	0	0.5246	.	0.5246	.	.	.
asenapine:clozapine	0	0	-1.5069	.	-1.5069	.	.	.
asenapine:flupenthixol	0	0	0.5089	.	0.5089	.	.	.
asenapine:fluphenazine	0	0	1.0004	.	1.0004	.	.	.
asenapine:haloperidol	1	0.44	1.4986	0.3509	2.4137	-2.0627	-2.34	0.0192

asenapine:iloperidone	0	0	-0.5014	.	-0.5014	.	.	.
asenapine:lurasidone	0	0	0.7028	.	0.7028	.	.	.
asenapine:olanzapine	1	0.24	-1.4631	-1.1000	-1.5776	0.4776	0.53	0.5961
asenapine:placebo	3	0.85	1.1169	1.2393	0.4093	0.8300	0.81	0.4161
asenapine:quetiapine	0	0	-0.3996	.	-0.3996	.	.	.
asenapine:risp_pali	0	0	-0.1030	.	-0.1030	.	.	.
asenapine:sertindole	0	0	-1.1909	.	-1.1909	.	.	.
asenapine:ziprasidone	0	0	2.7512	.	2.7512	.	.	.
asenapine:zotepine	0	0	-1.4226	.	-1.4226	.	.	.
cariprazine:clozapine	0	0	-2.0314	.	-2.0314	.	.	.
cariprazine:flupenthixol	0	0	-0.0157	.	-0.0157	.	.	.
cariprazine:fluphenazine	0	0	0.4758	.	0.4758	.	.	.
cariprazine:haloperidol	0	0	0.9740	.	0.9740	.	.	.
cariprazine:iloperidone	0	0	-1.0260	.	-1.0260	.	.	.
cariprazine:lurasidone	0	0	0.1783	.	0.1783	.	.	.
cariprazine:olanzapine	0	0	-1.9877	.	-1.9877	.	.	.
cariprazine:placebo	1	0.73	0.5923	0.7723	0.1042	0.6681	0.56	0.5752
cariprazine:quetiapine	0	0	-0.9242	.	-0.9242	.	.	.
cariprazine:risp_pali	1	0.64	-0.6276	-0.8277	-0.2662	-0.5615	-0.51	0.6117
cariprazine:sertindole	0	0	-1.7155	.	-1.7155	.	.	.
cariprazine:ziprasidone	0	0	2.2267	.	2.2267	.	.	.
cariprazine:zotepine	0	0	-1.9471	.	-1.9471	.	.	.
clozapine:flupenthixol	0	0	2.0157	.	2.0157	.	.	.
clozapine:fluphenazine	0	0	2.5072	.	2.5072	.	.	.
clozapine:haloperidol	2	0.73	3.0054	4.0952	0.0732	4.0220	2.51	0.0122
clozapine:iloperidone	0	0	1.0054	.	1.0054	.	.	.
clozapine:lurasidone	0	0	2.2097	.	2.2097	.	.	.
clozapine:olanzapine	2	0.58	0.0437	-1.2115	1.7794	-2.9909	-2.01	0.0442
clozapine:placebo	0	0	2.6238	.	2.6238	.	.	.
clozapine:quetiapine	0	0	1.1072	.	1.1072	.	.	.
clozapine:risp_pali	1	0.48	1.4038	1.9000	0.9544	0.9456	0.64	0.5226
clozapine:sertindole	0	0	0.3159	.	0.3159	.	.	.
clozapine:ziprasidone	0	0	4.2581	.	4.2581	.	.	.
clozapine:zotepine	1	0.58	0.0843	-0.6500	1.1068	-1.7568	-0.99	0.3243
flupenthixol:fluphenazine	0	0	0.4915	.	0.4915	.	.	.
flupenthixol:haloperidol	0	0	0.9897	.	0.9897	.	.	.
flupenthixol:iloperidone	0	0	-1.0103	.	-1.0103	.	.	.

flupenthixol:lurasidone	0	0	0.1940	.	0.1940	.	.	.
flupenthixol:olanzapine	1	0.49	-1.9720	-3.4000	-0.6260	-2.7740	-1.76	0.0777
flupenthixol:placebo	0	0	0.6081	.	0.6081	.	.	.
flupenthixol:quetiapine	0	0	-0.9085	.	-0.9085	.	.	.
flupenthixol:risp_pali	0	0	-0.6119	.	-0.6119	.	.	.
flupenthixol:sertindole	0	0	-1.6998	.	-1.6998	.	.	.
flupenthixol:ziprasidone	0	0	2.2424	.	2.2424	.	.	.
flupenthixol:zotepine	0	0	-1.9314	.	-1.9314	.	.	.
fluphenazine:haloperidol	0	0	0.4982	.	0.4982	.	.	.
fluphenazine:iloperidone	0	0	-1.5018	.	-1.5018	.	.	.
fluphenazine:lurasidone	0	0	-0.2975	.	-0.2975	.	.	.
fluphenazine:olanzapine	0	0	-2.4635	.	-2.4635	.	.	.
fluphenazine:placebo	0	0	0.1165	.	0.1165	.	.	.
fluphenazine:quetiapine	1	1.00	-1.4000	-1.4000	.	.	.	.
fluphenazine:risp_pali	0	0	-1.1034	.	-1.1034	.	.	.
fluphenazine:sertindole	0	0	-2.1913	.	-2.1913	.	.	.
fluphenazine:ziprasidone	0	0	1.7508	.	1.7508	.	.	.
fluphenazine:zotepine	0	0	-2.4229	.	-2.4229	.	.	.
haloperidol:iloperidone	1	1.00	-2.0000	-2.0000	.	.	.	.
haloperidol:lurasidone	0	0	-0.7957	.	-0.7957	.	.	.
haloperidol:olanzapine	5	0.59	-2.9617	-3.3612	-2.3916	-0.9696	-1.50	0.1340
haloperidol:placebo	1	0.24	-0.3816	0.0000	-0.5005	0.5005	0.64	0.5212
haloperidol:quetiapine	1	0.09	-1.8982	-1.9100	-1.8970	-0.0130	-0.01	0.9924
haloperidol:risp_pali	2	0.28	-1.6016	-1.9145	-1.4789	-0.4356	-0.59	0.5555
haloperidol:sertindole	0	0	-2.6895	.	-2.6895	.	.	.
haloperidol:ziprasidone	0	0	1.2527	.	1.2527	.	.	.
haloperidol:zotepine	1	0.62	-2.9211	-2.2500	-4.0068	1.7568	0.99	0.3243
iloperidone:lurasidone	0	0	1.2043	.	1.2043	.	.	.
iloperidone:olanzapine	0	0	-0.9617	.	-0.9617	.	.	.
iloperidone:placebo	0	0	1.6184	.	1.6184	.	.	.
iloperidone:quetiapine	0	0	0.1018	.	0.1018	.	.	.
iloperidone:risp_pali	0	0	0.3984	.	0.3984	.	.	.
iloperidone:sertindole	0	0	-0.6895	.	-0.6895	.	.	.
iloperidone:ziprasidone	0	0	3.2527	.	3.2527	.	.	.
iloperidone:zotepine	0	0	-0.9211	.	-0.9211	.	.	.
lurasidone:olanzapine	1	0.19	-2.1660	-3.1000	-1.9448	-1.1552	-1.32	0.1860
lurasidone:placebo	4	0.83	0.4141	0.7235	-1.0558	1.7793	2.17	0.0304

lurasidone:quetiapine	1	0.28	-1.1025	-0.4854	-1.3371	0.8516	1.02	0.3075
lurasidone:risp_pali	0	0	-0.8059	.	-0.8059	.	.	.
lurasidone:sertindole	0	0	-1.8938	.	-1.8938	.	.	.
lurasidone:ziprasidone	1	0.40	2.0484	0.3000	3.2180	-2.9180	-2.30	0.0216
lurasidone:zotepine	0	0	-2.1254	.	-2.1254	.	.	.
olanzapine:placebo	11	0.53	2.5801	2.4032	2.7830	-0.3798	-1.00	0.3157
olanzapine:quetiapine	3	0.25	1.0635	0.3726	1.2979	-0.9252	-1.46	0.1452
olanzapine:risp_pali	16	0.66	1.3601	1.3378	1.4037	-0.0659	-0.17	0.8644
olanzapine:sertindole	0	0	0.2722	.	0.2722	.	.	.
olanzapine:ziprasidone	2	0.68	4.2144	5.1518	2.2338	2.9180	2.30	0.0216
olanzapine:zotepine	0	0	0.0406	.	0.0406	.	.	.
placebo:quetiapine	5	0.57	-1.5165	-1.3575	-1.7279	0.3704	0.74	0.4584
placebo:risp_pali	15	0.66	-1.2200	-1.2901	-1.0821	-0.2079	-0.57	0.5668
placebo:sertindole	0	0	-2.3078	.	-2.3078	.	.	.
placebo:ziprasidone	0	0	1.6343	.	1.6343	.	.	.
placebo:zotepine	0	0	-2.5395	.	-2.5395	.	.	.
quetiapine:risp_pali	4	0.40	0.2966	0.5514	0.1261	0.4253	0.79	0.4267
quetiapine:sertindole	0	0	-0.7913	.	-0.7913	.	.	.
quetiapine:ziprasidone	0	0	3.1508	.	3.1508	.	.	.
quetiapine:zotepine	0	0	-1.0229	.	-1.0229	.	.	.
risp_pali:sertindole	2	1.00	-1.0879	-1.0879	.	.	.	.
risp_pali:ziprasidone	0	0	2.8543	.	2.8543	.	.	.
risp_pali:zotepine	0	0	-1.3195	.	-1.3195	.	.	.
sertindole:ziprasidone	0	0	3.9422	.	3.9422	.	.	.
sertindole:zotepine	0	0	-0.2316	.	-0.2316	.	.	.
ziprasidone:zotepine	0	0	-4.1738	.	-4.1738	.	.	.

eAppendix 5. Sensitivity Analyses

The sensitivity of our findings for all 7 outcomes were evaluated by repeating analyses following exclusion of studies examining patients with first episode psychosis (4 studies), treatment resistant schizophrenia (5 studies), and older adults (2 studies). After exclusion of these trials the hierarchy did not change considerably (see eFigures 11-17). Tests of heterogeneity and inconsistency were as follows:

For change in weight, τ was 0.61kg, and I^2 72.1%. The global Q score for inconsistency was 83.97 ($p<0.0001$), and significant hot-spots of inconsistency identified in 5 of 154 treatment comparisons.

For change in BMI, τ was 0.10kg/m^2, and I^2 26.1%. The global Q score for inconsistency was 12.48 ($p=0.13$), and no hot-spots of inconsistency identified in treatment comparisons.

For change in total cholesterol, τ was 0.08mmol/L, and I^2 46.5%. The global Q score for inconsistency was 26.37 ($p=0.02$), and a significant hot-spot of inconsistency identified in 1 of 91 treatment comparisons.

For change in LDL cholesterol, τ was 0.04mmol/L, and I^2 19.5%. The global Q score for inconsistency was 22.67 ($p=0.03$), and no hot-spots of inconsistency identified in treatment comparisons.

For change in HDL cholesterol, τ was 0.03mmol/L, and I^2 54.5%. The global Q score for inconsistency was 22.74 ($p=0.01$), and a significant hot-spot of inconsistency identified in 1 of 45 treatment comparisons.

For change in triglycerides, τ was 0.07mmol/L, and I^2 43.8%. The global Q score for inconsistency was 4.53 ($p=0.98$), and significant inconsistency hot-spots identified in 2 of 105 treatment comparisons.

For change in glucose, τ was 0.18mmol/L, and I^2 statistic 63.1%. The global Q score for inconsistency was 31.32 ($p<0.01$), and a significant hot-spot of inconsistency identified in 1 of the 103 treatment comparisons.

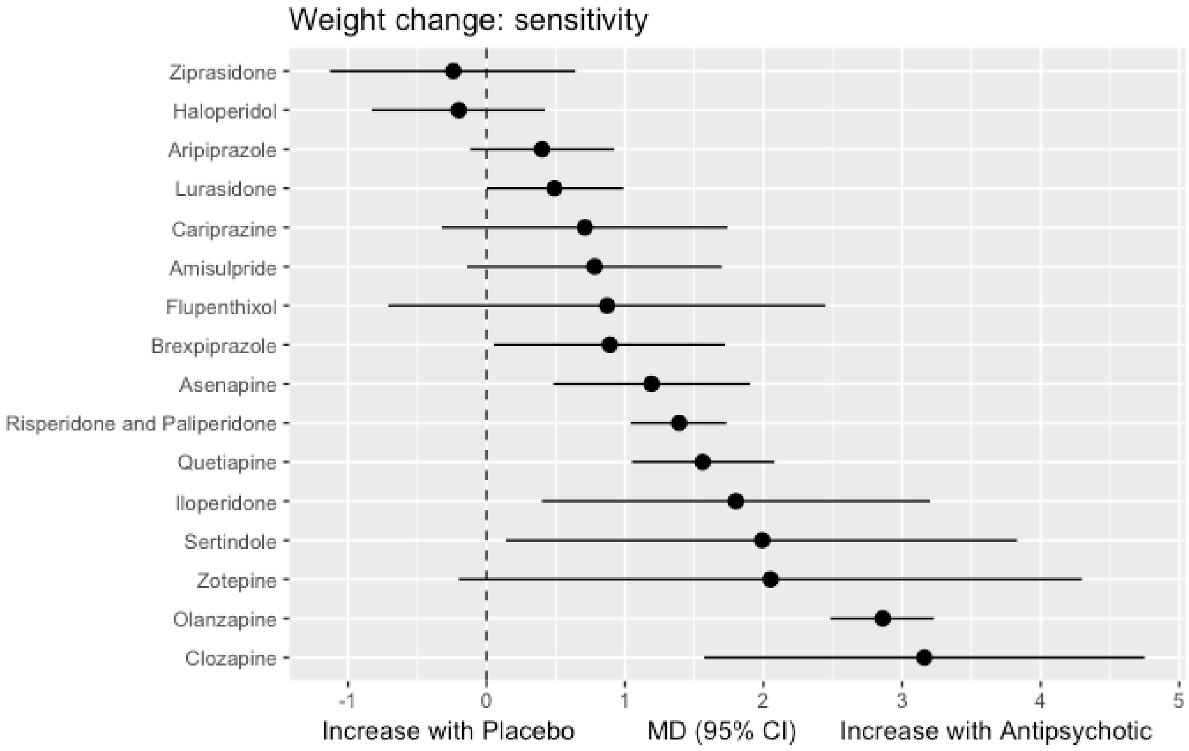

eFigure 11. Weight change, exclusion of studies examining first episode psychosis, treatment resistant schizophrenia, and older adults. Treatments are ranked according to mean difference (kg) compared to placebo. Mean differences are presented alongside 95% confidence intervals.

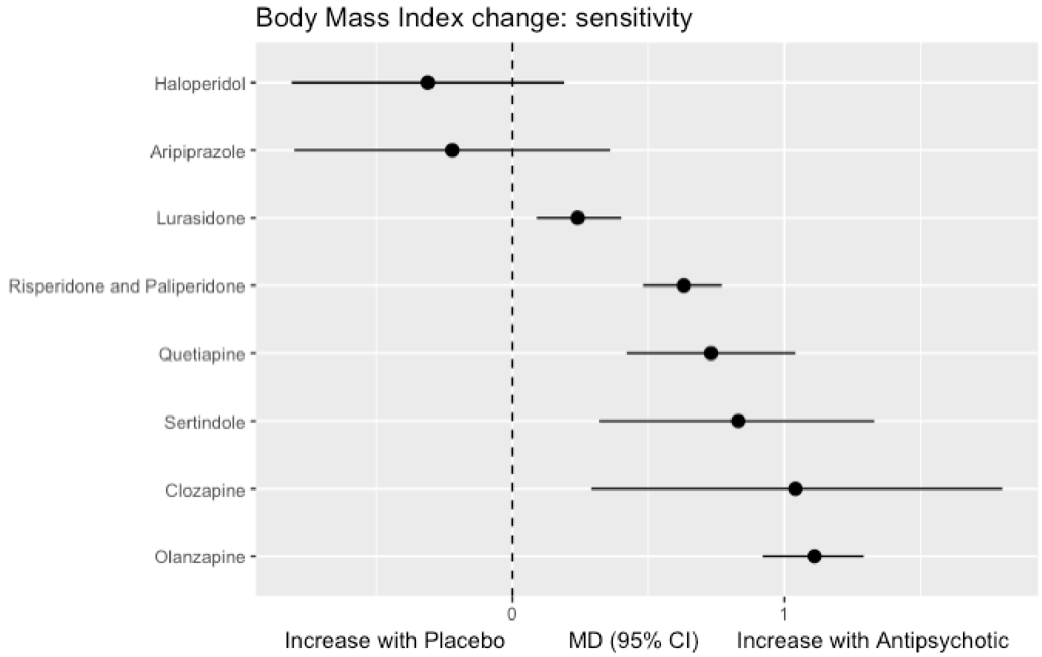

eFigure 12. Body Mass Index (BMI) change, exclusion of studies examining first episode psychosis, treatment resistant schizophrenia, and older adults. Treatments are ranked according to mean difference (kg/m^2) compared to placebo. Mean differences are presented alongside 95% confidence intervals.

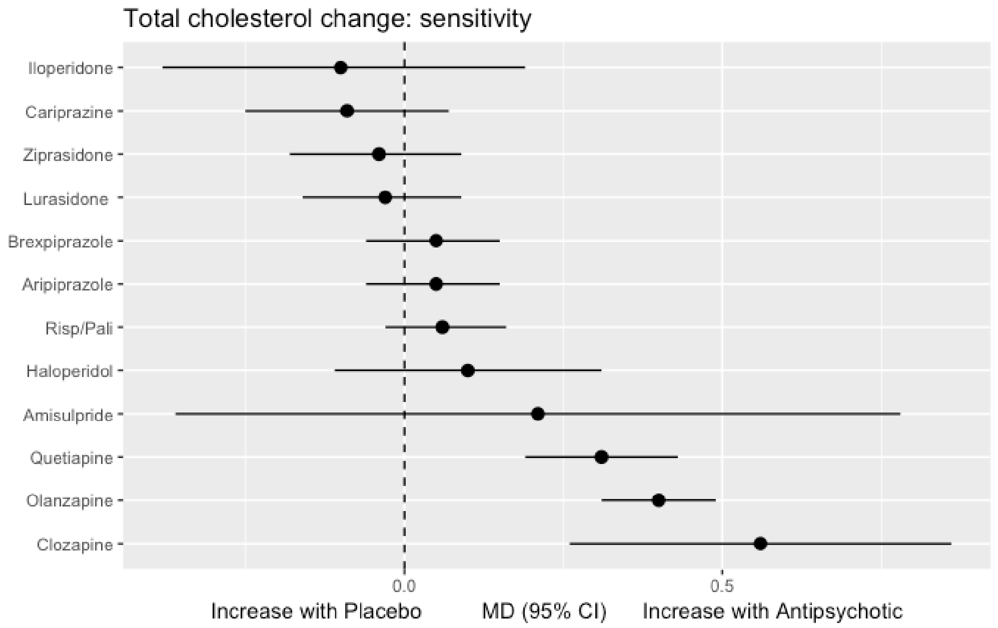

eFigure 13. Total cholesterol change, exclusion of studies examining first episode psychosis, treatment resistant schizophrenia, and older adults. Treatments are ranked according to mean difference (mmol/L) compared to placebo. Mean differences are presented alongside 95% confidence intervals.

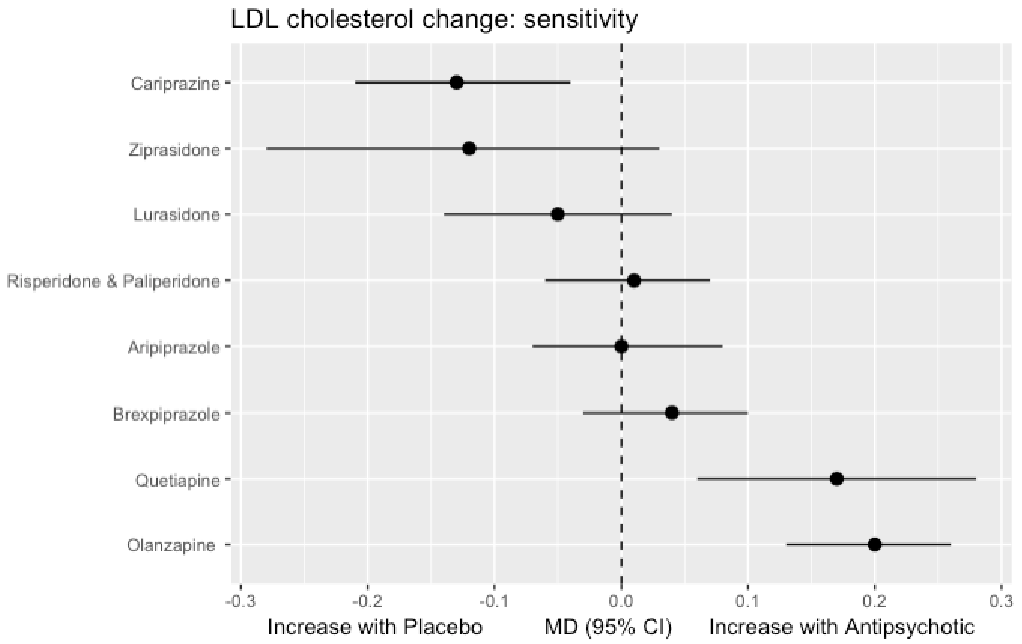

eFigure 14. Low Density Lipoprotein (LDL) cholesterol change, exclusion of studies examining first episode psychosis, treatment resistant schizophrenia, and older adults. Treatments are ranked according to mean difference (mmol/L) compared to placebo. Mean differences are presented alongside 95% confidence intervals.

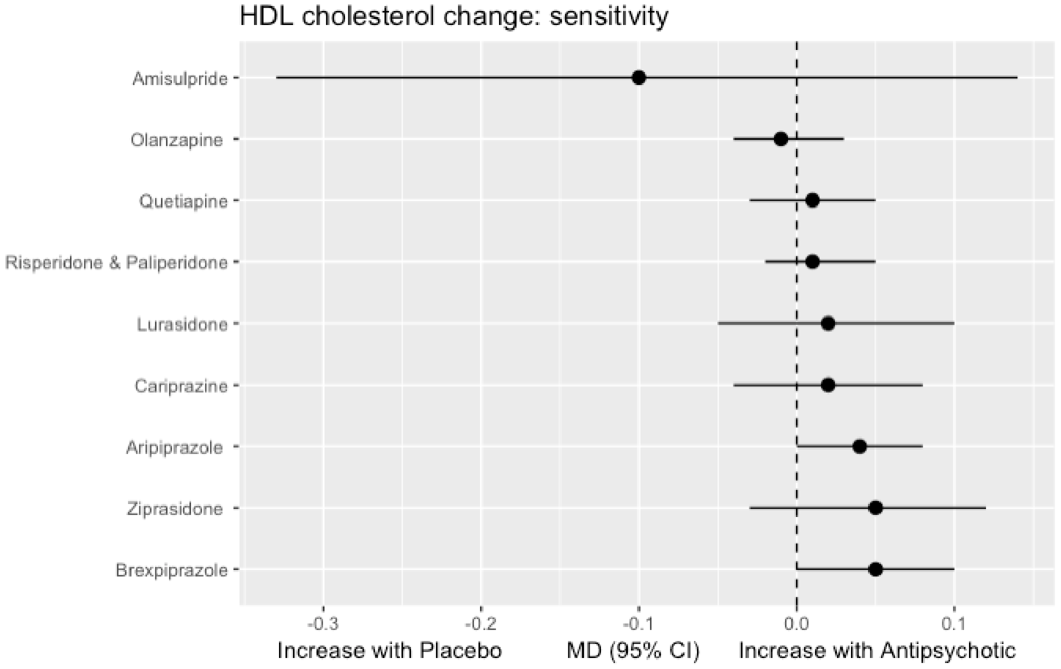

eFigure 15. High Density Lipoprotein (HDL) cholesterol change, exclusion of studies examining first episode psychosis, treatment resistant schizophrenia, and older adults. Treatments are ranked according to mean difference (mmol/L) compared to placebo. Mean differences are presented alongside 95% confidence intervals.

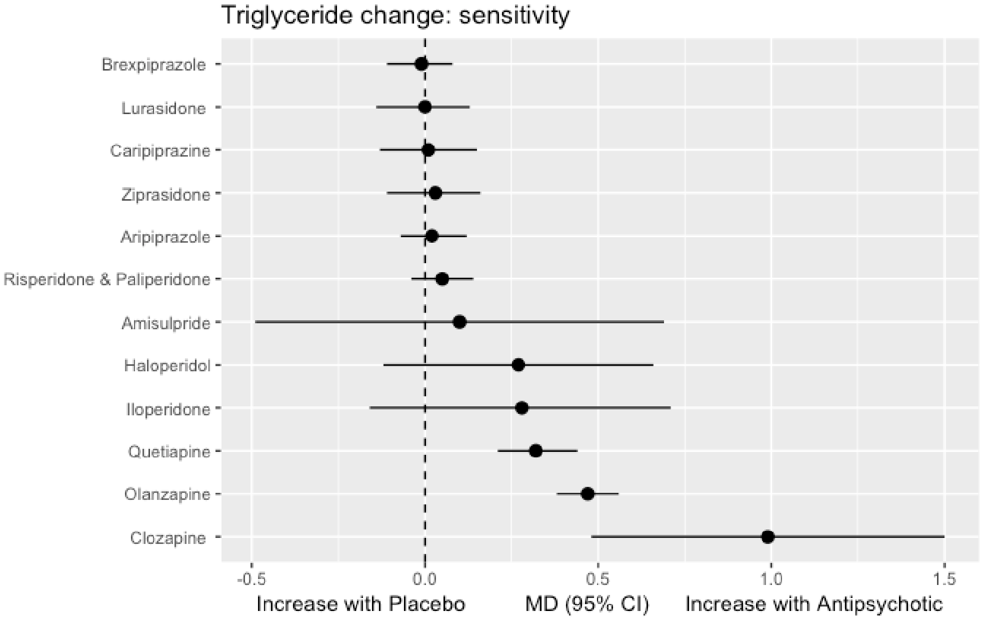

eFigure 16. Triglyceride change, exclusion of studies examining first episode psychosis, treatment resistant schizophrenia, and older adults. Treatments are ranked according to mean difference (mmol/L) compared to placebo. Mean differences are presented alongside 95% confidence intervals.

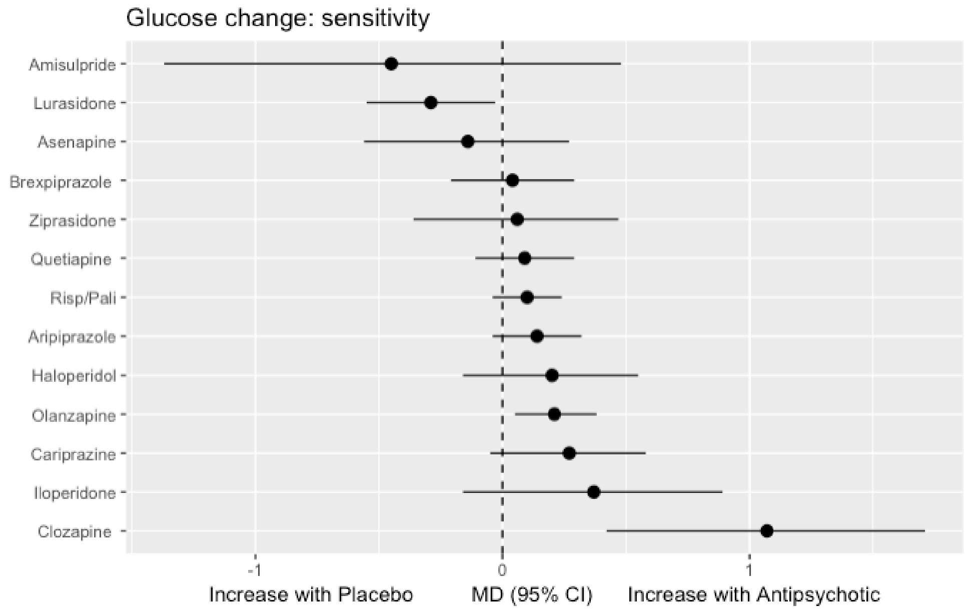

eFigure 17. Glucose change, exclusion of studies examining first episode psychosis, treatment resistant schizophrenia, and older adults. Treatments are ranked according to mean difference (mmol/L) compared to placebo. Mean differences are presented alongside 95% confidence intervals.

Supplementary References

1. Simon V, van Winkel R, De Hert M. Are weight gain and metabolic side effects of atypical antipsychotics dose dependent? A literature review. *J Clin Psychiatry.* 2009;70(7):1041-1050.
2. Howes OD, Bhatnagar A, Gaughran FP, Amiel SA, Murray RM, Pilowsky LS. A prospective study of impairment in glucose control caused by clozapine without changes in insulin resistance. *Am J Psychiat.* 2004;161(2):361-363.
3. Moher D, Liberati A, Tetzlaff J, Altman DG, Grp P. Preferred Reporting Items for Systematic Reviews and Meta-Analyses: The PRISMA Statement. *Journal of Clinical Epidemiology.* 2009;62(10):1006-1012.
4. Leucht S, Cipriani A, Spineli L, et al. Comparative efficacy and tolerability of 15 antipsychotic drugs in schizophrenia: a multiple-treatments meta-analysis. *Lancet.* 2013;382(9896):951-962.
5. Higgins PT, Green S. Cochrane Handbook for Systematic Reviews of Interventions Version 5.1.0 (updated March 2011). 2011.
6. Meltzer HY, Massey BW. The role of serotonin receptors in the action of atypical antipsychotic drugs. *Current Opinion in Pharmacology.* 2011;11(1):59-67.
7. R Core Team. R: A language and environment for statistical computing. 2017; https://www.R-project.org/. Accessed February 19, 2018.
8. Egger M, Davey Smith G, Schneider M, Minder C. Bias in meta-analysis detected by a simple, graphical test. *BMJ.* 1997;315(7109):629-634.
9. Efthimiou O, Debray TP, van Valkenhoef G, et al. GetReal in network meta-analysis: a review of the methodology. *Res Synth Methods.* 2016;7(3):236-263.
10. Hildrum B, Mykletun A, Hole T, Midthjell K, Dahl AA. Age-specific prevalence of the metabolic syndrome defined by the International Diabetes Federation and the National Cholesterol Education Program: the Norwegian HUNT 2 study. *Bmc Public Health.* 2007;7:220.
11. Pradhan AD. Sex differences in the metabolic syndrome: implications for cardiovascular health in women. *Clin Chem.* 2014;60(1):44-52.
12. Liu J, Hanley AJG, Young TK, Harris SB, Zinman B. Characteristics and prevalence of the metabolic syndrome among three ethnic groups in Canada. *Int J Obesity.* 2006;30(4):669-676.
13. Rucker G. Network meta-analysis, electrical networks and graph theory. *Res Synth Methods.* 2012;3(4):312-324.
14. Rücker G, Krahn U, König J, Efthimiou O, Schwarzer G. netmeta: Network Meta-Analysis using Frequentist Methods. https://github.com/guido-s/netmeta. 2019.
15. Rummel-Kluge C, Komossa K, Schwarz S, et al. Head-to-head comparisons of metabolic side effects of second generation antipsychotics in the treatment of schizophrenia: A systematic review and meta-analysis. *Schizophrenia Research.* 2010;123(2-3):225-233.
16. Zhang YY, Liu YY, Su YY, et al. The metabolic side effects of 12 antipsychotic drugs used for the treatment of schizophrenia on glucose: a network meta-analysis. *Bmc Psychiatry.* 2017;17.
17. Salanti G, Del Giovane C, Chaimani A, Caldwell DM, Higgins JPT. Evaluating the Quality of Evidence from a Network Meta-Analysis. *Plos One.* 2014;9(7).
18. Dias S, Welton NJ, Caldwell DM, Ades AE. Checking consistency in mixed treatment comparison meta-analysis. *Stat Med.* 2010;29(7-8):932-944.
19. Eckel RH, Kahn SE, Ferrannini E, et al. Obesity and Type 2 Diabetes: What Can Be Unified and What Needs to Be Individualized? *Diabetes Care.* 2011;34(6):1424-1430.
20. Leucht S, Samara M, Heres S, Davis JM. Dose Equivalents for Antipsychotic Drugs: The DDD Method. *Schizophr Bull.* 2016;42 Suppl 1:S90-94.

21. Inada T, Inagaki A. Psychotropic dose equivalence in Japan. *Psychiatry Clin Neurosci.* 2015;69(8):440-447.
22. Riley RD, Thompson JR, Abrams KR. An alternative model for bivariate random-effects meta-analysis when the within-study correlations are unknown. *Biostatistics.* 2008;9(1):172-186.
23. Higgins JPT, Altman DG, Gotzsche PC, et al. The Cochrane Collaboration's tool for assessing risk of bias in randomised trials. *Bmj-Brit Med J.* 2011;343.
24. Salanti G, Del Giovane C, Chaimani A, Caldwell DM, Higgins JP. Evaluating the quality of evidence from a network meta-analysis. *PLoS One.* 2014;9(7):e99682.
25. Nikolakopoulou A, Higgins JP, Papakonstantinou T, et al. Assessing Confidence in the Results of Network Meta-Analysis (Cinema). *bioRxiv: pre-print.* 2019.
26. Ascher-Svanum H, Stensland MD, Kinon BJ, Tollefson GD. Weight gain as a prognostic indicator of therapeutic improvement during acute treatment of schizophrenia with placebo or active antipsychotic. *Journal of Psychopharmacology.* 2005;19(6 SUPPL.):110-117.
27. Beasley CM, Jr., Sanger T, Satterlee W, Tollefson G, Tran P, Hamilton S. Olanzapine versus placebo: results of a double-blind, fixed-dose olanzapine trial. *Psychopharmacology (Berl).* 1996;124(1-2):159-167.
28. Azorin JM, Strub N, Loft H. A double-blind, controlled study of sertindole versus risperidone in the treatment of moderate-to-severe schizophrenia. *International Clinical Psychopharmacology.* 2006;21(1):49-56.
29. Baymiller SP, Ball P, McMahon RP, Buchanan RW. Weight and blood pressure change during clozapine treatment. *Clinical Neuropharmacology.* 2002;25(4):202-206.
30. Bhowmick S, Hazra A, Ghosh M. Amisulpride versus olanzapine in the treatment of schizophrenia in Indian patients: randomized controlled trial. *Aust N Z J Psychiatry.* 2010;44(3):237-242.
31. Beasley CM, Jr., Tollefson G, Tran P, Satterlee W, Sanger T, Hamilton S. Olanzapine versus placebo and haloperidol: acute phase results of the North American double-blind olanzapine trial. *Neuropsychopharmacology.* 1996;14(2):111-123.
32. Bossie CA, Fu DJ, Sliwa JK, Alphs L, Ma YW. Tolerability of initiation doses of once-monthly paliperidone palmitate in patients with recently diagnosed schizophrenia in an acute treatment trial. *Therapeutic Advances in Psychopharmacology.* 2011;1(4):111-124.
33. Breier A, Berg PH, Thakore JH, et al. Olanzapine versus ziprasidone: results of a 28-week double-blind study in patients with schizophrenia. *American Journal of Psychiatry.* 2005;162(10):1879-1887.
34. Buckley PF, Goldstein JM, Emsley RA. Efficacy and tolerability of quetiapine in poorly responsive, chronic schizophrenia. *Schizophrenia Research.* 2004;66(2-3):143-150.
35. Cantillon M, Prakash A, Alexander A, Ings R, Sweitzer D, Bhat L. Dopamine serotonin stabilizer RP5063: A randomized, double-blind, placebo-controlled multicenter trial of safety and efficacy in exacerbation of schizophrenia or schizoaffective disorder. *Schizophrenia Research.* 2017;189:126-133.
36. Canuso CM, Dirks B, Carothers J, et al. Randomized, double-blind, placebo-controlled study of paliperidone extended-release and quetiapine in inpatients with recently exacerbated schizophrenia. *The American Journal of Psychiatry.* 2009;166(6):691-701.
37. Canuso CM, Lindenmayer JP, Kosik-Gonzalez C, et al. A Randomized, Double-Blind, Placebo-Controlled Study of 2 Dose Ranges of Paliperidone Extended-Release in the Treatment of Subjects With Schizoaffective Disorder. *J Clin Psychiat.* 2010;71(5):587-598.
38. Canuso CM, Schooler N, Carothers J, et al. Paliperidone extended-release in schizoaffective disorder: a randomized, controlled study comparing a flexible dose with placebo in patients treated with and without antidepressants and/or mood stabilizers. *J Clin Psychopharmacol.* 2010;30(5):487-495.

39. Canuso CM, Bossie CA, Amatniek J, Turkoz I, Pandina G, Cornblatt B. Paliperidone extended-release tablets in patients with recently diagnosed schizophrenia. *Early Interv Psychia.* 2010;4(1):64-78.
40. Conley RR, Mahmoud R. A randomized double-blind study of risperidone and olanzapine in the treatment of schizophrenia or schizoaffective disorder. *American Journal of Psychiatry.* 2001;158(5):765-774.
41. Conley RR, Kelly DL, Nelson MW, et al. Risperidone, quetiapine, and fluphenazine in the treatment of patients with therapy-refractory schizophrenia. *Clinical Neuropharmacology.* 2005;28(4):163-168.
42. Coppola D, Melkote R, Lannie C, et al. Efficacy and Safety of Paliperidone Extended Release 1.5 mg/day-A Double-blind, Placebo- and Active-Controlled, Study in the Treatment of Patients with Schizophrenia. *Psychopharmacol Bull.* 2011;44(2):54-72.
43. Correll CU, Skuban A, Hobart M, et al. Efficacy of brexpiprazole in patients with acute schizophrenia: Review of three randomized, double-blind, placebo-controlled studies. *Schizophrenia Research.* 2016;174(1-3):82-92.
44. Correll CU, Skuban A, Ouyang J, et al. Efficacy and Safety of Brexpiprazole for the Treatment of Acute Schizophrenia: A 6-Week Randomized, Double-Blind, Placebo-Controlled Trial. *Am J Psychiat.* 2015;172(9):870-880.
45. Cutler AJ, Tran-Johnson T, Kalali A, Astrom M, Brecher M, Meulien D. A failed 6-week, randomized, double-blind, placebo-controlled study of once-daily extended release quetiapine fumarate in patients with acute schizophrenia: Lessons learned. *Psychopharmacol Bull.* 2011;44(4):37-69.
46. Chan HY, Lin WW, Lin SK, et al. Efficacy and safety of aripiprazole in the acute treatment of schizophrenia in Chinese patients with risperidone as an active control: A randomized trial. *Journal of Clinical Psychiatry.* 2007;68(1):29-36.
47. Chan HY, Chang CJ, Chiang SC, et al. A randomised controlled study of risperidone and olanzapine for schizophrenic patients with neuroleptic-induced acute dystonia or parkinsonism. *J Psychopharmacol.* 2010;24(1):91-98.
48. Chen JJ, Chan HY, Chen CH, Gau SS, Hwu HG. Risperidone and olanzapine versus another first generation antipsychotic in patients with schizophrenia inadequately responsive to first generation antipsychotics. *Pharmacopsychiatry.* 2011;45(2):64-71.
49. Czobor P, Volavka J, Sheitman B, et al. Antipsychotic-induced weight gain and therapeutic response: a differential association. *J Clin Psychopharmacol.* 2002;22(3):244-251.
50. Davidson M, Emsley R, Kramer M, et al. Efficacy, safety and early response of paliperidone extended-release tablets (paliperidone ER): Results of a 6-week, randomized, placebo-controlled study(vol 93, pg 117, 2007). *Schizophrenia Research.* 2007;96(1-3):273-274.
51. Del Valle MC, Loebel AD, Murray S, Yang R, Harrison DJ, Cuffel BJ. Change in framingham risk score in patients with schizophrenia: A post hoc analysis of a randomized, double-blind, 6-week trial of ziprasidone and olanzapine. *Primary Care Companion to the Journal of Clinical Psychiatry.* 2006;8(6):329-333.
52. Downing ACM, Kinon BJ, Millen BA, et al. A double-blind, placebo-controlled comparator study of LY2140023 monohydrate in patients with schizophrenia. *Bmc Psychiatry.* 2014;14 (1) (no pagination)(351).
53. Durgam S, Starace A, Li D, et al. An evaluation of the safety and efficacy of cariprazine in patients with acute exacerbation of schizophrenia: A phase II, randomized clinical trial. *Schizophrenia Research.* 2014;152(2-3):450-457.
54. Durgam S, Cutler AJ, Lu K, et al. Cariprazine in Acute Exacerbation of Schizophrenia: A Fixed-Dose, Phase 3, Randomized, Double-Blind, Placebo- and Active-Controlled Trial. *J Clin Psychiat.* 2015;76(12):E1574-+.

55. Durgam S, Litman RE, Papadakis K, Li DY, Nemeth G, Laszlovszky I. Cariprazine in the treatment of schizophrenia: a proof-of-concept trial. *Int Clin Psychopharm.* 2016;31(2):61-68.
56. Earley W, Durgam S, Lu K, Laszlovszky I, Debelle M, Kane JM. Safety and tolerability of cariprazine in patients with acute exacerbation of schizophrenia: A pooled analysis of four phase II/III randomized, double-blind, placebo-controlled studies. *Int Clin Psychopharm.* 2017;32(6):319-328.
57. Kane JM, Zukin S, Wang Y, et al. Efficacy and Safety of Cariprazine in Acute Exacerbation of Schizophrenia: Results from an International, Phase III Clinical Trial. *J Clin Psychopharm.* 2015;35(4):367-373.
58. Fu DJ, Bossie CA, Sliwa JK, Ma YW, Alphs L. Paliperidone palmitate versus oral risperidone and risperidone long-acting injection in patients with recently diagnosed schizophrenia: a tolerability and efficacy comparison. *International Clinical Psychopharmacology.* 2014;29(1):45-55.
59. Gattaz W, Diehl A, Geuppert M, et al. Olanzapine versus Flupenthixol in the Treatment of Inpatients with Schizophrenia: A Randomized Double-Blind Trial. *Pharmacopsychiatry.* 2004;37(6):279-285.
60. Gopal S, Hough DW, Xu H, et al. Efficacy and safety of paliperidone palmitate in adult patients with acutely symptomatic schizophrenia: A randomized, double-blind, placebo-controlled, dose-response study. *International Clinical Psychopharmacology.* 2010;25(5):247-256.
61. Grootens KP, van Veelen NM, Peuskens J, et al. Ziprasidone vs olanzapine in recent-onset schizophrenia and schizoaffective disorder: results of an 8-week double-blind randomized controlled trial. *Schizophrenia Bulletin.* 2011;37(2):352-361.
62. Hardy TA, Henry RR, Forrester TD, et al. Impact of olanzapine or risperidone treatment on insulin sensitivity in schizophrenia or schizoaffective disorder. *Diabetes, Obesity and Metabolism.* 2011;13(8):726-735.
63. Hatta K, Sato K, Hamakawa H, et al. Effectiveness of second-generation antipsychotics with acute-phase schizophrenia. *Schizophr Res.* 2009;113(1):49-55.
64. Higuchi T, Ishigooka J, Iyo M, et al. Lurasidone in the treatment of schizophrenia: Results of a double-blind, placebo-controlled trial in Asian patients. *Asia Pac Psychiatry.* 2019;11(2):e12352.
65. Hwang TJ, Lin SK, Lin HN. Efficacy and safety of zotepine for the treatment of Taiwanese schizophrenic patients: A double-blind comparison with haloperidol. *Journal of the Formosan Medical Association.* 2001;100(12):811-816.
66. Hwang TJ, Lee SM, Sun HJ, et al. Amisulpride versus risperidone in the treatment of schizophrenic patients: A double-blind pilot study in Taiwan. *Journal of the Formosan Medical Association.* 2003;102(1):30-36.
67. Ishigooka J, Iwashita S, Tadori Y. Efficacy and safety of brexpiprazole for the treatment of acute schizophrenia in Japan: A 6-week, randomized, double-blind, placebo-controlled study. *Psychiatry & Clinical Neurosciences.* 2018;72(9):692-700.
68. Jeste DV, Barak Y, Madhusoodanan S, Grossman F, Gharabawi G. International Multisite Double-Blind Trial of the Atypical Antipsychotics Risperidone and Olanzapine in 175 Elderly Patients with Chronic Schizophrenia. *American Journal of Geriatric Psychiatry.* 2003;11(6):638-647.
69. Kahn RS, Schulz SC, Palazov VD, et al. Efficacy and tolerability of once-daily extended release quetiapine fumarate in acute schizophrenia: A randomized, double-blind, placebo-controlled study. *J Clin Psychiat.* 2007;68(6):832-842.
70. Kane J, Canas F, Kramer M, et al. Treatment of schizophrenia with paliperidone extended-release tablets: A 6-week placebo-controlled trial. *Schizophrenia Research.* 2007;90(1-3):147-161.

71. Kane JM, Lauriello J, Laska E, Di Marino M, Wolfgang CD. Long-term efficacy and safety of iloperidone: Results from 3 clinical trials for the treatment of schizophrenia. *Journal of Clinical Psychopharmacology.* 2008;28(7 SUPPL. 1):S29-S35.
72. Kane JM, Cohen M, Zhao J, Alphs L, Panagides J. Efficacy and safety of asenapine in a placebo- and haloperidol-controlled trial in patients with acute exacerbation of schizophrenia. *J Clin Psychopharm.* 2010;30(2):106-115.
73. Kane JM, Potkin SG, Daniel DG, Buckley PF. A double-blind, randomized study comparing the efficacy and safety of sertindole and risperidone in patients with treatment-resistant schizophrenia. *Journal of Clinical Psychiatry.* 2011;72(2):194-204.
74. Kane JM, Peters-Strickland T, Baker RA, et al. Aripiprazole once-monthly in the acute treatment of schizophrenia: Findings from a 12-week, randomized, double-blind, placebo-controlled study. *J Clin Psychiat.* 2014;75(11):1254-1260.
75. Kelly DL, Conley RR, Love RC, Morrison JA, McMahon RP. Metabolic risk with second-generation antipsychotic treatment: A double-blind randomized 8-week trial of risperidone and olanzapine. *Annals of Clinical Psychiatry.* 2008;20(2):71-78.
76. Kinon BJ, Zhang L, Millen BA, et al. A Multicenter, Inpatient, Phase 2, Double-Blind, Placebo-Controlled Dose-Ranging Study of LY2140023 Monohydrate in Patients With DSM-IV Schizophrenia. *J Clin Psychopharm.* 2011;31(3):349-355.
77. Kinoshita T, Bai YM, Kim JH, Miyake M, Oshima N. Efficacy and safety of asenapine in Asian patients with an acute exacerbation of schizophrenia: a multicentre, randomized, double-blind, 6-week, placebo-controlled study. *Psychopharmacology.* 2016;233(14):2663-2674.
78. Kramer M, Litman R, Hough D, et al. Paliperidone palmitate, a potential long-acting treatment for patients with schizophrenia. Results of a randomized, double-blind, placebo-controlled efficacy and safety study. *Int J Neuropsychopharmacol.* 2010;13(5):635-647.
79. Krakowski M, Czobor P, Citrome L. Weight gain, metabolic parameters, and the impact of race in aggressive inpatients randomized to double-blind clozapine, olanzapine or haloperidol. *Schizophrenia Research.* 2009;110(1-3):95-102.
80. Landbloom R, MacKle M, Wu X, et al. Asenapine for the treatment of adults with an acute exacerbation of schizophrenia: Results from a randomized, double-blind, fixed-dose, placebo-controlled trial with olanzapine as an active control. *Cns Spectrums.* 2017;22(4):333-341.
81. Lauriello J, Lambert T, Andersen S, Lin D, Taylor CC, McDonnell D. An 8-week, double-blind, randomized, placebo-controlled study of olanzapine long-acting injection in acutely ill patients with schizophrenia. *J Clin Psychiat.* 2008;69(5):790-799.
82. Lasser RA, Mao L, Gharabawi G. Smokers and nonsmokers equally affected by olanzapine-induced weight gain: metabolic implications. *Schizophrenia Research.* 2004;66(2-3):163-167.
83. Lawson WB, Herman BK, Loebel A, Lazariciu I, Malik M. Ziprasidone in Black patients with schizophrenia: analysis of four short-term, double-blind studies. *Cns Spectrums.* 2009;14(9):478-486.
84. Li H, Luo J, Wang C, et al. Efficacy and safety of aripiprazole in Chinese Han schizophrenia subjects: A randomized, double-blind, active parallel-controlled, multicenter clinical trial. *Schizophrenia Research.* 2014;157(1-3):112-119.
85. Lin CC, Chiu HJ, Chen JY, et al. Switching from clozapine to zotepine in patients with schizophrenia: a 12-week prospective, randomized, rater blind, and parallel study. *J Clin Psychopharmacol.* 2013;33(2):211-214.
86. Lieberman JA, Tollefson G, Tohen M, et al. Comparative efficacy and safety of atypical and conventional antipsychotic drugs in first-episode psychosis: A randomized, double-blind trial of olanzapine versus haloperidol. *American Journal of Psychiatry.* 2003;160(8):1396-1404.
87. Lieberman JA, Davis RE, Correll CU, et al. ITI-007 for the Treatment of Schizophrenia: A 4-Week Randomized, Double-Blind, Controlled Trial. *Biol Psychiatry.* 2016;79(12):952-961.

88. Lindenmayer JP, Czobor P, Volavka J, et al. Changes in glucose and cholesterol levels in patients with schizophrenia treated with typical or atypical antipsychotics. *American Journal of Psychiatry.* 2003;160(2):290-296.
89. Lindenmayer JP, Brown D, Liu S, Brecher M, Meulien D. The efficacy and tolerability of once-daily extended release quetiapine fumarate in hospitalized patients with acute schizophrenia: a 6-week randomized, double-blind, placebo-controlled study. *Psychopharmacol Bull.* 2008;41(3):11-35.
90. Litman RE, Smith MA, Desai DG, Simpson T, Sweitzer D, Kanes SJ. The Selective Neurokinin 3 Antagonist AZD2624 Does Not Improve Symptoms or Cognition in Schizophrenia A Proof-of-Principle Study. *J Clin Psychopharm.* 2014;34(2):199-204.
91. Litman RE, Smith MA, Doherty JJ, et al. AZD8529, a positive allosteric modulator at the mGluR2 receptor, does not improve symptoms in schizophrenia: A proof of principle study. *Schizophrenia Research.* 2016;172(1-3):152-157.
92. Loebel A, Cucchiaro J, Sarma K, et al. Efficacy and safety of lurasidone 80mg/day and 160mg/day in the treatment of schizophrenia: A randomized, double-blind, placebo- and active-controlled trial. *Schizophrenia Research.* 2013;145(1-3):101-109.
93. Loebel A, Silva R, Goldman R, et al. Lurasidone dose escalation in early nonresponding patients with schizophrenia: A randomized, placebo-controlled study. *J Clin Psychiat.* 2016;77(12):1672-1680.
94. Marder SR, Kramer M, Ford L, et al. Efficacy and Safety of Paliperidone Extended-Release Tablets: Results of a 6-Week, Randomized, Placebo-Controlled Study. *Biol Psychiat.* 2007;62(12):1363-1370.
95. Martin S, Ljo H, Peuskens J, et al. A double-blind, randomised comparative trial of amisulpride versus olanzapine in the treatment of schizophrenia: short-term results at two months. *Current Medical Research & Opinion.* 2002;18(6):355-362.
96. Meltzer HY, Bobo WV, Nuamah IF, et al. Efficacy and tolerability of oral paliperidone extended-release tablets in the treatment of acute schizophrenia: Pooled data from three 6-week, placebo-controlled studies. *Journal of Clinical Psychiatry.* 2008;69(5):817-829.
97. Meltzer HY, Cucchiaro J, Silva R, et al. Lurasidone in the treatment of schizophrenia: a randomized, double-blind, placebo- and olanzapine-controlled study. *Am J Psychiatry.* 2011;168(9):957-967.
98. Meulien D, Huizar K, Brecher M. Safety and tolerability of once-daily extended release quetiapine fumarate in acute schizophrenia: Pooled data from randomised, double-blind, placebo-controlled studies. *Human Psychopharmacology.* 2010;25(2):103-115.
99. Nasrallah HA, Gopal S, Gassmann-Mayer C, et al. A controlled, evidence-based trial of paliperidone palmitate, a Long-acting injectable antipsychotic, in schizophrenia. *Neuropsychopharmacology.* 2010;35(10):2072-2082.
100. Nasrallah HA, Silva R, Phillips D, et al. Lurasidone for the treatment of acutely psychotic patients with schizophrenia: a 6-week, randomized, placebo-controlled study. *J Psychiatr Res.* 2013;47(5):670-677.
101. Nasrallah HA, Newcomer JW, Risinger R, et al. Effect of aripiprazole lauroxil on metabolic and endocrine profiles and related safety considerations among patients with acute schizophrenia. *Journal of Clinical Psychiatry.* 2016;77(11):1519-1525.
102. Nasser AF, Henderson DC, Fava M, et al. Efficacy, Safety, and Tolerability of RBP-7000 Once-Monthly Risperidone for the Treatment of Acute Schizophrenia: An 8-Week, Randomized, Double-Blind, Placebo-Controlled, Multicenter Phase 3 Study. *J Clin Psychopharmacol.* 2016;36(2):130-140.
103. Newcomer JW, Meyer JM, Baker RA, et al. Changes in non-high-density lipoprotein cholesterol levels and triglyceride/high-density lipoprotein cholesterol ratios among patients randomized to aripiprazole versus olanzapine. *Schizophrenia Research.* 2008;106(2-3):300-307.

104. Newcomer JW, Campos JA, Marcus RN, et al. A multicenter, randomized, double-blind study of the effects of aripiprazole in overweight subjects with schizophrenia or schizoaffective disorder switched from olanzapine. *Journal of Clinical Psychiatry.* 2008;69(7):1046-1056.
105. Ogasa M, Kimura T, Nakamura M, Guarino J. Lurasidone in the treatment of schizophrenia: a 6-week, placebo-controlled study. *Psychopharmacology (Berl).* 2013;225(3):519-530.
106. Patil ST, Zhang L, Martenyi F, et al. Activation of mGlu2/3 receptors as a new approach to treat schizophrenia: A randomized Phase 2 clinical trial. *Nature Medicine.* 2007;13(9):1102-1107.
107. Patel JK, Buckley PF, Woolson S, et al. Metabolic profiles of second-generation antipsychotics in early psychosis: findings from the CAFE study. *Schizophrenia Research.* 2009;111(1-3):9-16.
108. Potkin SG, Saha AR, Kujawa MJ, et al. Aripiprazole, an antipsychotic with a novel mechanism of action, and risperidone vs placebo in patients with schizophrenia and schizoaffective disorder. *Arch Gen Psychiat.* 2003;60(7):681-690.
109. Potkin SG, Gharabawi GM, Greenspan AJ, et al. A double-blind comparison of risperidone, quetiapine and placebo in patients with schizophrenia experiencing an acute exacerbation requiring hospitalization. *Schizophrenia Research.* 2006;85(1-3):254-265.
110. Potkin SG, Ogasa M, Cucchiaro J, Loebel A. Double-blind comparison of the safety and efficacy of lurasidone and ziprasidone in clinically stable outpatients with schizophrenia or schizoaffective disorder. *Schizophrenia Research.* 2011;132(2-3):101-107.
111. Potkin SG, Kimura T, Guarino J. A 6-week, double-blind, placebo- and haloperidol-controlled, phase II study of lurasidone in patients with acute schizophrenia. *Therapeutic Advances in Psychopharmacology.* 2015;5(6):322-331.
112. Riedel M, Muller N, Strassnig M, et al. Quetiapine has equivalent efficacy and superior tolerability to risperidone in the treatment of schizophrenia with predominantly negative symptoms. *European Archives of Psychiatry and Clinical Neuroscience.* 2005;255(6):432-437.
113. Riedel M, Muller N, Spellmann I, et al. Efficacy of olanzapine versus quetiapine on cognitive dysfunctions in patients with an acute episode of schizophrenia. *European archives of psychiatry and clinical neuroscience.* 2007;257(7):402-412.
114. Robinson DG, Gallego JA, John M, et al. A Randomized Comparison of Aripiprazole and Risperidone for the Acute Treatment of First-Episode Schizophrenia and Related Disorders: 3-Month Outcomes. *Schizophrenia Bulletin.* 2015;41(6):1227-1236.
115. Saddichha S, Manjunatha N, Ameen S, Akhtar S. Effect of olanzapine, risperidone, and haloperidol treatment on weight and body mass index in first-episode schizophrenia patients in India: A randomized, double-blind, controlled, prospective study. *Journal of Clinical Psychiatry.* 2007;68(11):1793-1798.
116. Schmidt ME, Kent JM, Daly E, et al. A double-blind, randomized, placebo-controlled study with JNJ-37822681, a novel, highly selective, fast dissociating D2 receptor antagonist in the treatment of acute exacerbation of schizophrenia. *European Neuropsychopharmacology.* 2012;22(10):721-733.
117. Shen JH, Zhao Y, Rosenzweig-Lipson S, et al. A 6-week randomized, double-blind, placebo-controlled, comparator referenced trial of vabicaserin in acute schizophrenia. *J Psychiatr Res.* 2014;53:14-22.
118. Sliwa JK, Bossie CA, Ma YW, Alphs L. Effects of acute paliperidone palmitate treatment in subjects with schizophrenia recently treated with oral risperidone. *Schizophrenia Research.* 2011;132(1):28-34.
119. Tollefson GD, Beasley CM, Jr., Tran PV, et al. Olanzapine versus haloperidol in the treatment of schizophrenia and schizoaffective and schizophreniform disorders: results of an international collaborative trial. *American Journal of Psychiatry.* 1997;154(4):457-465.
120. Tzimos A, Samokhvalov V, Kramer M, et al. Safety and tolerability of oral paliperidone extended-release tablets in elderly patients with schizophrenia: A double-blind, placebo-

controlled study with six-month open-label extension. *Am J Geriat Psychiat.* 2008;16(1):31-43.

121. Vanelle JM, Douki S. A double-blind randomised comparative trial of amisulpride versus olanzapine for 2 months in the treatment of subjects with schizophrenia and comorbid depression. *European Psychiatry.* 2006;21(8):523-530.

122. Wang X, Savage R, Borisov A, et al. Efficacy of risperidone versus olanzapine in patients with schizophrenia previously on chronic conventional antipsychotic therapy: A switch study. *Journal of Psychiatric Research.* 2006;40(7):669-676.

123. Wetzel H, Grunder G, Hillert A, et al. Amisulpride versus flupentixol in schizophrenia with predominantly positive symptomatology: A double-blind controlled study comparing a selective D2-like antagonist to a mixed D1-/D2-like antagonist. *Psychopharmacology.* 1998;137(3):223-232.

124. Zhong KX, Sweitzer DE, Hamer RM, Lieberman JA. Comparison of quetiapine and risperidone in the treatment of schizophrenia: A randomized, double-blind, flexible-dose, 8-week study. *Journal of Clinical Psychiatry.* 2006;67(7):1093-1103.

125. Daniel DG, Zimbroff DL, Potkin SG, Reeves KR, Harrigan EP, Lakshminarayanan M. Ziprasidone 80 mg/day and 160 mg/day in the acute exacerbation of schizophrenia and schizoaffective disorde: A 6-week placebo-controlled trial. *Neuropsychopharmacology.* 1999;20(5):491-505.

126. Keck P, Jr., Buffenstein A, Ferguson J, et al. Ziprasidone 40 and 120 mg/day in the acute exacerbation of schizophrenia and schizoaffective disorder: a 4-week placebo-controlled trial. *Psychopharmacology.* 1998;140(2):173-184.

127. Pandina GJ, Lindenmayer JP, Lull J, et al. A randomized, placebo-controlled study to assess the efficacy and safety of 3 doses of paliperidone palmitate in adults with acutely exacerbated schizophrenia. *Journal of Clinical Psychopharmacology.* 2010;30(3):235-244.
